# Accessible, robust ultrahigh-throughput microbiome analysis via integration of uniform droplet-templated emulsification and FACS

**DOI:** 10.1371/journal.pone.0356887

**Published:** 2026-09-09

**Authors:** Wannes Nauwynck, Karoline Faust, Nico Boon

**Affiliations:** 1 Department of Biotechnology, Center for Microbial Ecology and Technology (CMET), Ghent University, Ghent, Belgium; 2 Department of Microbiology, Immunology and Transplantation, Laboratory of Molecular Bacteriology (Rega Institute), KU Leuven, Leuven, Belgium; 3 Centre for Advanced Process Technology for Urban Resource recovery (CAPTURE), Ghent, Belgium; University of Waterloo, CANADA

## Abstract

Microbial phenotypes vary at the single-cell level, shaping key community traits like resilience and adaptability. Yet, current methods either lack resolution (e.g., culturing, sequencing), are too costly, or technically complex, limiting widespread use. To address this gap, we introduce and validate a workflow called DE-SWIRL (Double Emulsion–Sorting Workflow with sImple, Rapid emuLsification), an accessible, low-cost workflow enabling ultrahigh-throughput (~10^7^ microcultures/experiment) screening of individual microbial cells. DE-SWIRL integrates a published droplet-templated emulsification protocol producing uniform double emulsions from monodisperse single emulsions with Fluorescence-Activated Cell Sorting (FACS). We validated that double emulsions of 6 and 24 pL can be reliably formed, with ~45% droplet survival. To address persistent large-particle contaminants, we validated a gating strategy and show it enables accurate screening and sorting. When starting from a monodisperse single emulsion population, oil layer variability is higher for droplet-templated emulsification than for on-chip microfluidics, but maintains a comparably uniform core emulsion while offering substantial time savings. We demonstrate DE-SWIRL's utility by isolating viable strains from a synthetic community with up to 99% sorting purity and isolating droplet cocultures from a mixed community. This workflow provides a fast, accessible, and affordable workflow for screening entire microbiomes at a single-cell level using a fluorescent assay of interest.

## Introduction

Microbial communities are complex and dynamic, comprising up to thousands of species whose composition can change rapidly over time [1–3]. These communities display functional heterogeneity on the level of a single cell, with cells belonging to the same strain able to exert different functionalities [4]. Understanding these single-cell functionalities and their heterogeneity is vital for advancing microbial ecology, as they drive community-level properties such as microbiome composition and function (e.g., photosynthesis, fiber degradation, nutrient cycling). However, traditional methods fail to capture this fine-grained dynamic functional heterogeneity. Current cultivation-based methods, while allowing functionality assessment, are low-throughput, slow and often fail to reflect *in situ* phenotypes [5]. High-throughput sequencing, though rapid, cannot directly measure functionality and aggregates data from all microbiome members, limiting interpretability [6]. To understand the true functional capacity and dynamics of microbiomes, innovative approaches are needed that are rapid, high-throughput and high resolution.

Single-cell functionality screening is emerging as a powerful method that fills this gap [7]. Single cells are encapsulated in tens of millions of microdroplets together with fluorescent reporter strains or dyes after which the droplets are analyzed and sorted. A range of single-cell functionalities have been measured this way, from metabolite production (e.g., tryptophan production in members of a *Saccharomyces cerevisiae* library [8]) and enzyme activity (e.g., methane monooxygenase activity of methanotrophs, [9]) to bacterial interactions (e.g., killing interactions of oral bear microbiome members on *Staphylococcus aureus,* [10]). Since a myriad of fluorescent reporters can be used, custom assays for desired functionalities can be rapidly developed and implemented as demonstrated by Bowman *et al.* (2021) [11].

Despite its potential to reveal functional heterogeneity within microbiomes, single-cell functional screening remains limited to a small number of studies from specialized laboratories [12]. A typical workflow involves three steps: encapsulating single cells in water-in-oil droplets (single emulsions), converting these into FACS-compatible water-in-oil-in-water emulsions (double emulsions), and analysing them by FACS. Because each droplet acts as an individual assay well, variation in droplet volume introduces variability in final readouts. Producing uniform double emulsions is therefore essential to minimize technical variability and to reveal true biological heterogeneity.

The aforementioned three steps differ substantially in their technical demands. Single-cell encapsulation can be performed using a commercial chip, syringe pumps and a stereomicroscope, requiring limited investment and expertise. In contrast, double-emulsion production is more complex, requires chips with patterned wettability [13], and needs to be monitored using a high-speed camera mounted on an inverted microscope, representing an additional investment of approximately US$20,000 (see Table A in S1 File). FACS analysis is generally easier to implement because suitable instruments are often available through institutional flow cytometry facilities. Most gains in accessibility can be achieved by replacing chip-based double emulsion production with a simpler step.

Droplet-templated emulsification (DTE) methods are a collection of bulk emulsification techniques that create relatively uniform double emulsions starting from a uniform single emulsion template population. Unlike on-chip methods, DTE methods rely on simple means of generating shear, such as pipetting, flicking or vortexing [14,15]. During DTE, single emulsions are transferred into an aqueous continuous phase and subjected to shear. This shear emulsifies the oil phase around the single emulsions, progressively emulsifying large oil droplets containing multiple single emulsions into smaller double emulsions until they ideally contain one inner droplet (core emulsion) [16]. When performed well, the core emulsions remain intact and act as size templates, resulting in a double-emulsion population that retains much of the uniformity of the parent single-emulsion population.

DTE methods differ primarily in how shear is applied. Sukovich et al. (2017) first demonstrated the generation of FACS-compatible double emulsions using bulk manipulations such as repeated pipetting, or a combination of vortexing and flicking [14]. This work established the feasibility of integrating DTE with FACS. Lin et al. (2022) applied an adapted version of the multi-step Sukovich protocol (flicking and vortexing [15]). Although these studies established the feasibility of DTE, their protocols involved several operations, likely reducing repeatability and robustness, and produced core emulsions with broader size distributions than the parent single-emulsion populations, limiting their usefulness for functionality screening workflows [14,15]. More recently, Wang et al. (2022) [16] reported a DTE protocol based on a single vortex manipulation that appeared to yield highly uniform core emulsions. Among the limited protocols available, this therefore represented the most suitable starting point for developing a rapid and robust workflow that preserves high droplet monodispersity. However, its performance at droplet sizes relevant to single-cell screening, its added variability and its compatibility with FACS were not established.

Here, we first evaluate the performance of the DTE protocol to convert chip-produced single emulsions to double emulsions, across relevant droplet sizes, we then identify FACS parameters that mitigate DTE-introduced artefacts, and subsequently compare the uniformity of the resulting cores inside the DTE-produced double emulsions against chip-produced double emulsions. Finally, we validate the complete workflow – which we call DE-SWIRL (Double Emulsion–Sorting Workflow with sImple, Rapid emulsification) – using a synthetic microbial community and a mixed microbial community which we cultivate in droplets and sort based on biomass-associated fluorescence, demonstrating its compatibility with microbiological systems. To allow rapid implementation, a detailed DTE protocol can be found in Supplementary Data and a video protocol can be found in the data repository associated with this paper.

## Materials and methods

For extended materials and methods and a detailed DE-SWIRL protocol, see S1 File.

### Single emulsion production

Water-in-oil emulsions were generated using two syringe pumps (TSE systems type 540060, Berlin, Germany). Syringes were connected to the PTFE tubing (1/16” OD, 0.3 mm ID, Deutsch & Neumann, Hennigsdorf, Germany) using Luer lock-HPLC fittings (IDEX Health and Science, Oak Harbor, USA). The aqueous phase varied depending on the population made (Table B in S1 File). The standard flow rates used were 250 µL/hr for the aqueous phase and 300 µL/hr for the oil phase; to decrease droplet size the oil flow rate was increased. Microfluidic operations were monitored using an inverted microscope (Zeiss Axiovert 135) and footage was recorded using a high-speed camera (S-Motion Mono 2.6 GB High Speed Camera, AOS Technologies AG, Dätwill, Switzerland). All droplet populations around 4–10 pL in volume were made using channels of 10 µm height. For droplets around 20–35 pL in size, channels were used that are 20 µm in height.

For all droplet samples except the synthetic community sample, the oil phase consisted of 2.5 w% Ionic PEG-Krytox (Krytox 157 FSH, Chemours, Wilmington, USA) in HFE-7500 (3M, Zwijndrecht, Belgium). The oil phase of the caproic acid reactor sample consisted of 1 w% Pico-Surf (Sphere Fluidics, Cambridge, UK) in HFE-7500 to avoid biocompatibility issues that might arise with the ionic Krytox surfactant. [17,18]

### Monodisperse double emulsion production through droplet-templated emulsification

Depending on the application, a volume ranging from 1.5 mL to 4 mL of double emulsion buffer was added to a standard 50 mL conical-bottom centrifuge tube (Greiner Bio-One, Vilvoorde, Belgium). For sterile applications the buffer was filter sterilized using a sterile 0.2 µm regenerated cellulose membrane filter. The desired amount of creamed single emulsions was aspirated and the pipette tip was wiped off with a dust-free tissue. The single emulsions were then added into the aqueous buffer by submerging the micropipette tip to the bottom of the centrifuge tube and pushing the plunger down slowly. The centrifuge tube was vortexed using an Analog Vortex Mixer (VWR, Leuven, Belgium) at the desired speed for 15 seconds. For successful emulsification, a thin film needs to be formed on the wall of the tube. This thin film needs to remain stable for the entire emulsification process. For a detailed protocol see S1 File; for an accompanying video protocol see data repository. For vortex parameters and population examples see Table B and C in S1 File.

### General double emulsion flow cytometry

Depending on the amount of single emulsions and the amount of double emulsion buffer used during emulsification, the double emulsions were diluted using double emulsion buffer (1x PBS + 2 w% Tween20) or were loaded directly into the flow cytometer. Before introducing the sample into the flow cytometer the double emulsions were shaken gently to resuspend them. Independent of the type of flow cytometer (Vulcan or FACSMelody, BD, Erembodegem, Belgium) samples were run at the lowest sample flow rate to keep the emulsions intact, and the samples were agitated at 300 rpm to counteract sedimentation during acquisition.

### DE-SWIRL of synthetic community on FACSMelody™ (BD)

After DTE, double emulsions were stained by adding SYTO17 (5 mM in DMSO) to the double emulsion buffer to a final concentration of 5 µM. This staining solution was incubated at 37 °C for 30 minutes under gentle rocking to allow homogeneous staining. The double emulsions were washed three times with sterile double emulsion buffer by repeatedly letting double emulsions sediment and removing supernatant.

Stained double emulsions were loaded and analysed on a BD FACSMelody™ (Cell sorter with fixed 100 µm nozzle 2-laser, 6-colour (2–4) configuration). Voltages were initially adjusted so that the majority of the analysed population was visible on the scatterplots and were kept the same between experiments. Single-core double emulsions (SCDE) were gated and gating was checked by sorting 200 events into 100 µL 1x PBS + 2 w% Tween20 in a round bottom 96-well plate. An inverted microscope was used to count the sorted double emulsions. After gating was confirmed to be accurate, the platform of the cell sorter was thoroughly decontaminated using 70% ethanol after which a sterile flat-bottom 96-well plate was filled with 50 µL sterile 1x PBS. Single biomass-positive (top 5% of biomass-containing double emulsions) droplets were sorted into the wells using the purity sort mode. After sorting, 200 µL sterile LB was added to the wells. Plates were incubated at 37 °C for two weeks, after which growth was evaluated by eye and species identity was determined using a blue transilluminator. This was possible since there were two strains in our synthetic community, one YFP-fluorescent *Escherichia coli* MG1655 Y3 and one non-fluorescent *Comamonas nitrativorans* 23310.

### Statistical data analysis

All statistical analyses were conducted in R (version 4.0.3) using functions from base R and the betareg package [19].

#### Bootstrap analysis.

To compare the core radius between the DTE method and the chip method, we used bootstrap resampling with replacement since the data exhibited non-normality. A bootstrap function was defined to calculate the difference in means between these two conditions. This function was applied to 10,000 bootstrap resamples to generate a distribution of the mean differences. To formally test the hypothesis of no difference between the conditions, we created a pooled dataset under the null hypothesis. Both datasets were shifted to have the same mean. These pooled and shifted data were then bootstrapped 10,000 times to generate a distribution under the null hypothesis. The observed difference was compared to this distribution to calculate the p-value, providing a formal test of significance. This was performed in the same way to compare the oil layer thickness between the DTE method and the chip method.

#### Correlation analysis.

To determine whether the Cy5-A signal is correlated with oil width, we conducted a Spearman's rank correlation analysis. We computed the Spearman's rank correlation coefficient between the Cy5-A signal and oil width. The correlation coefficient was calculated along with its significance level.

#### Beta regression analysis.

To model the relationship between our response variables (conversion efficiency and uniformity) and the experimental variables (buffer volume, vortex speed, and droplet size), we employed beta regression models [20]. Beta regression is appropriate given the nature of our dependent variables, both bounded between 0 and 1 and displaying non-normality. The beta regression model was specified with a logit link function. The assumptions of the beta regression model, including link function appropriateness, and normality of residuals were checked using diagnostic plots, indicating that the residuals were generally well-behaved, with a few outliers noted (Figs A-D in S1 File).

## Results

### 1. Emulsification parameters directly control DTE compatibility with FACS

On-chip emulsification produces highly uniform double emulsions populations (i.e., a uniform core emulsion and a uniform oil layer), but it is a relatively slow process. This is due to its serial mode of emulsification, where single emulsions are emulsified one by one. DTE, on the other hand, emulsifies all emulsions simultaneously, meaning that 5 µL can be emulsified in the same time as 200 µL of single emulsions. In addition, this process only takes 30 seconds in total, making it compatible with quick optimization (Table 1). This rapid process makes DTE an attractive alternative for high-throughput workflows and facilitates the testing of different emulsification conditions.

**Table 1:** State-of-the-art comparison between on-chip and droplet-templated emulsification based on previously published literature. The present work addresses several knowledge gaps indicated as “Unknown”.

	On-chip emulsification	Droplet-templated emulsification (protocol of Wang et al. (2022) [16])
*Processing time needed to emulsify 100 µL single emulsions*	30 minutes	30 seconds
*Emulsification mode*	Sequential	Simultaneous
*Equipment*	Double emulsification chip	Benchtop vortex
*Extra variability introduced in core emulsions*	Low	Unknown
*Variability of oil layer*	Low	Unknown
*Compatibility with FACS*	Good	Unknown

To determine whether the DTE protocol of Wang et al. [16] is compatible with the high-throughput requirements of FACS-based functionality screening, we evaluated its ability to produce double emulsions of FACS-sortable size with minimal artefacts and a sufficiently high yield. Specifically, we investigated if this DTE protocol can generate double emulsions with (i) high conversion efficiency (i.e., percentage of single emulsions that were successfully converted to desired double emulsions; see Fig E in S1 File) and (ii) high uniformity (i.e., percentage of single-core double emulsions (SCDEs) over the total number of double emulsion objects counted; see Fig E in S1 File), characterized by a minimized fraction of multi-core double emulsions (MCDEs).

**Fig 1:**
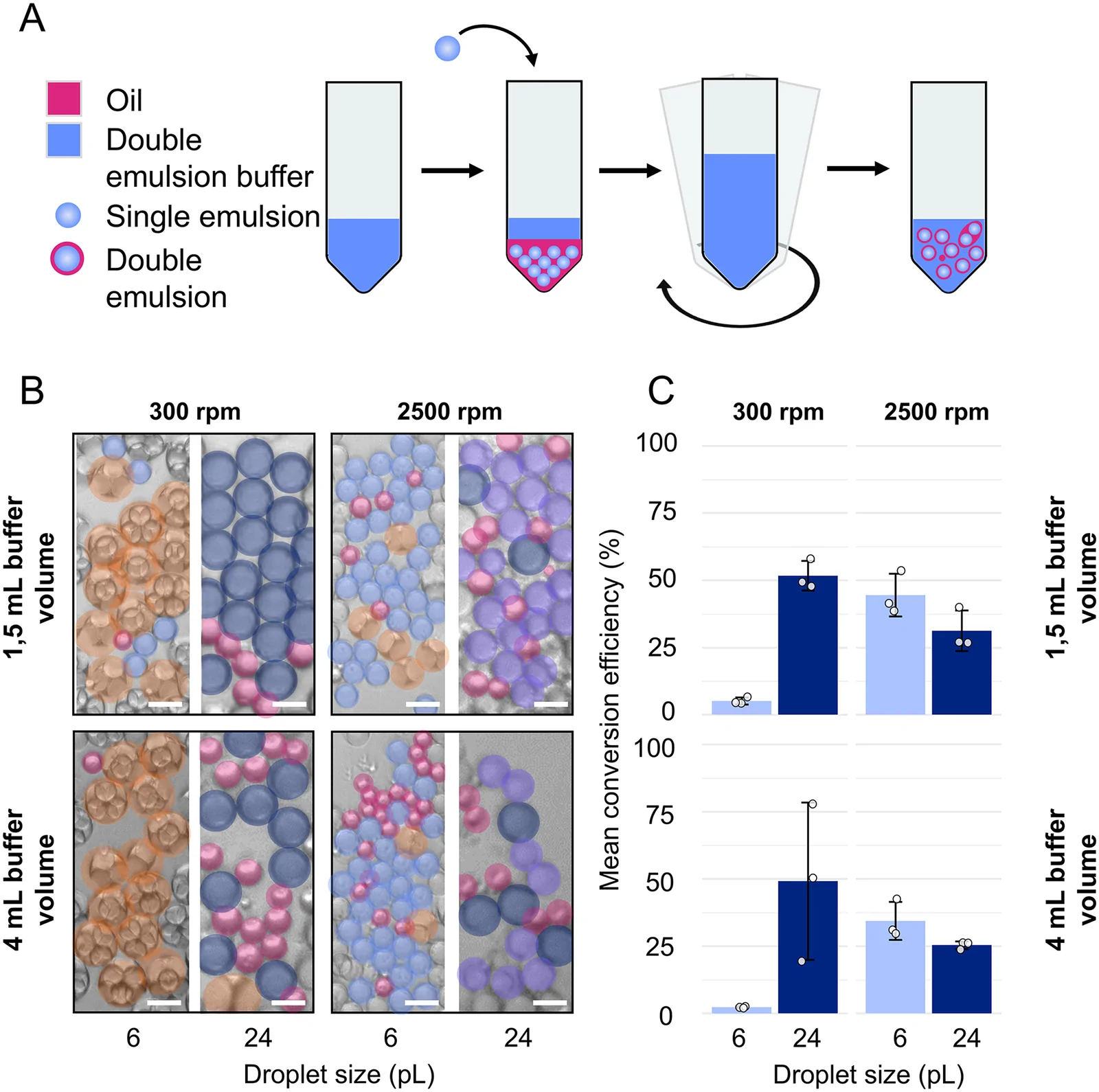
Droplet-templated emulsification (DTE) workflow and optimization of conversion efficiency. A Schematic of DTE protocol (see S1 File for detailed protocol). From left to right: Single emulsions are added to the vortexing container into double emulsion buffer, after which they are vortexed and double emulsions are formed. Colours used: double emulsion buffer: dark blue, single emulsions: light blue gradient, oil: red. B Brightfield images of double emulsion populations of different DTE protocols. Blue: single-core double emulsions, orange: multi-core double emulsions, purple: broken core double emulsions, red: oil droplets. Layout for both B and C: rows indicate 1.5 mL and 4 mL buffer volume, columns from left to right, indicate vortex speeds of 300 rpm and 2500 rpm. Maximum conversion efficiency is seen at different DTE protocols depending on droplet size. The scale bars are 40 µm. Full-size microscopy pictures can be found in S1 File (Tables B and C in S1 File for overview). C Barplots of mean conversion efficiency of different DTE regimes. Error bars indicate standard deviation (n = 3). The white points are the values obtained for each replicate. Conversion efficiency is defined as the fraction of single emulsions that are added to the DTE process that make it into intact single-core double emulsions.

To do this, we generated two monodisperse single emulsion populations using a microfluidic chip. Each population had a different average droplet size that fell within the FACS-sortable range (6 pL single emulsions with diameter 23 µm and 24 pL single emulsions with diameter 36 µm). We subjected these single emulsions to extreme DTE regimes where we varied the parameters between a minimal and a maximal value. For DTE, the single-emulsion suspension was added to an aqueous buffer and vortexed for 15 s, converting the water-in-oil droplets into water-in-oil-in-water double emulsions (Fig 1A). Two vortex speeds were evaluated: 300 rpm and 2500 rpm, the minimum and maximum vortex speeds of our benchtop vortex. Two buffer volumes were evaluated: 1.5 mL and 4 mL. We repeated the DTE protocol three times. Buffer volume and vortexing speed were selected since they are easily changed and therefore allow rapid optimization. After emulsification, we determined the conversion efficiency and uniformity with microscopy and imaging flow cytometry (Fig 1A, Table C in S1 File). This approach allows us to deduce how the emulsification parameters impact DTE outcomes, allowing an optimization rationale for DTE.

Fig 1B shows the make-up of the different DTE populations. Four types of emulsions can be seen: single-core double emulsion (SCDE), MCDE, broken core double emulsions and oil droplets. SCDE are double emulsions with a single core; they are the target double emulsion for ultrahigh-throughput assays. MCDE are double emulsions that contain two or more cores and are undesirable. They are large and potentially introduce problems for FACS analysis by destabilizing the FACS stream [21]. Broken core double emulsions are double emulsions that are formed by breaking the template single emulsion. They are undesirable in the context of functionality screens since they increase signal variability. The last type of emulsion consists of oil droplets (oil-in-water emulsion). Oil droplets originate from the interstitial oil present between the single emulsions and are unavoidable regardless of the emulsification method.

At 1.5 mL emulsification volume, increasing vortex speed to 2500 rpm significantly enhances conversion efficiency for 6 pL droplets, from 5.11 ± 1.29% at 300 rpm to 44.49 ± 7.93%. A similar trend is seen with 4 mL buffer (from 2.26 ± 0.28% at 300 rpm to 34.38 ± 7.09% at 2500 rpm, Fig 1B and C). Microscopy images show an increase in SCDE fraction with higher vortex speed. At 300 rpm, most cores remain within MCDE, whereas at 2500 rpm, the MCDE fraction visibly decreases as they convert to SCDE (Fig 1B).

Conversely, 24 pL droplets show reduced conversion efficiency with increased vortex speed, decreasing from 51.67 ± 5.51% to 31.21 ± 7.56% for 1.5 mL buffer and from 49.16 ± 29.27% to 25.38 ± 1.42% for 4 mL buffer, due to core breakup (Fig 1C). Supporting these observations, the fitted beta regression model (Materials and methods, Fig A and Fig B, in S1 File) indicates significant effects of vortex speed (p < 0.005) and droplet radius (p < 0.005) on DTE conversion efficiency, while buffer volume shows no significant effect (p = 0.49). The strong interaction between vortex speed and droplet radius (p < 0.005) underscores the droplet size-dependent outcomes of DTE at different vortex speeds.

Besides conversion efficiency, we investigated the influence of the DTE parameters on population uniformity (Fig F in S1 File). This is an important parameter for FACS-compatibility since the presence of large particles such as MCDE can interfere with FACS sorting performance and data quality [21]. We express population uniformity as the percentage of double emulsions that are SCDE (see Materials and Methods). Uniformity follows similar trends to conversion efficiency. For 6 pL droplets, increasing the vortex speed from 300 rpm to 2500 rpm increases uniformity. At a buffer volume of 1.5 mL, the percentage of SCDE increases from 29.85 ± 9.43% to 85.06 ± 3.02%. At a 4 mL buffer volume, SCDE rises from 22.33 ± 5.82% at 300 rpm to 85.00 ± 3.38% at 2500 rpm, indicating that higher speeds significantly improve uniformity.

In contrast, 24 pL droplets show decreased uniformity at higher speeds. At 1.5 mL, SCDE drops from 98.03 ± 1.03% at 300 rpm to 28.01 ± 1.42% at 2500 rpm. At 4 mL, the decrease is from 88.01 ± 3.95% to 27.13 ± 6.05%. Beta regression confirms significant effects of vortex speed, droplet radius and buffer volume on uniformity (Fig C and Fig D, in S1 File).

Our results indicate that DTE protocols can achieve high conversion efficiency and uniformity for both ends of the sortable droplet size range. The optimal vortex speed is dependent on droplet size, however, with smaller droplets benefitting from high speeds and larger droplets from low speeds. Despite achieving near-uniformity through DTE, MCDE were present in every one of our trials. Although we did not perform fine-grained optimization to obtain perfectly uniform populations (i.e., 100% SCDE), we assume, based on our trials, that the persistent presence of MCDE is inherent to DTE. This might be due to the random break-up processes or simply due to variations in performance. Since MCDE interfere with FACS-analysis, we focused on validating analysis and sorting of near-uniform populations.

### 2. Side scatter-based gating allows exclusion of artefacts from analysis

Previously we saw that none of the tested DTE conditions showed perfect conversion to 100% SCDE; there were always MCDE present (Figs M-U in S1 File). To enable FACS analysis of populations that are near-uniform, we first aimed to validate a gating approach that selects only SCDE. This gating approach was validated using imaging flow cytometry, which combines imaging data and flow cytometric data, allowing the association of flow cytometric parameters with the morphology of each event. To assess the ability of flow cytometry to distinguish between SCDE and MCDE, we investigated two ends of the DTE optimization spectrum: a perfectly uniform population comprising only SCDE and oil droplets (Uniformity = 100.0 ± 0.0%, made using chip-based emulsification) and a non-uniform population characterized by a high prevalence of MCDE (Uniformity = 61.8 ± 1.3%). We rated the performance of all flow cytometry parameter pairs by their capability to specifically gate the desired SCDE population without including artefacts (i.e., oil droplets and MCDE). The performance metric used was the false positivity rate, defined here as the percentage of artefacts included when we applied an automated gating algorithm on the SCDE population (see Materials and methods).

In a perfectly uniform population, there are only oil droplets and SCDE to discern. The best distinguishing scatter-based parameter pairs were FSC-A – SSC-A, FSC-A – FSC-W, and FSC-W – FSC-H (see Fig X in S1 File for explanation) with 1.60 ± 0.73%, 1.62 ± 0.75% and 1.79 ± 0.54% false positives in their gate, respectively. Looking further, most parameter pairs performed similarly well, including only about 2% false positives when gating for SCDE (Fig Y in S1 File).

**Fig 2:**
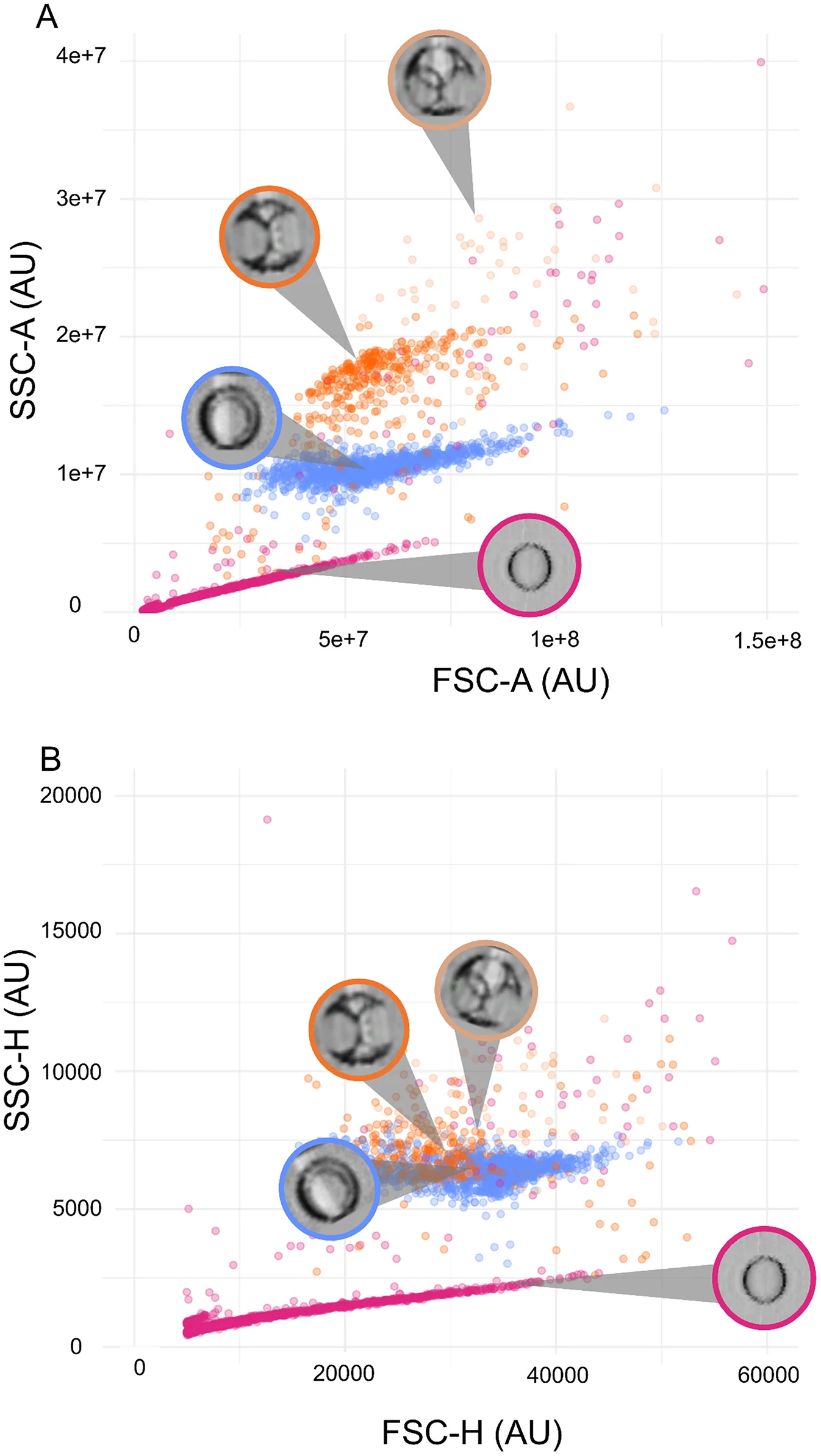
Flow cytometric discrimination of single-core double emulsions from artefacts. A Two scatterplots showing the same flow cytometric data of a non-uniform DTE population. The data is plotted using two different parameter pairs that exhibit good separation (A) and bad separation (B) of single-core double emulsion (SCDE, blue) and artefacts (magenta: oil droplets, orange: double-core double emulsion and peach: triple-core double emulsion). A allows high-specificity gating of SCDE, B does not. A x-axis: FSC-A (Arbitrary Units (AU)), y-axis: SSC-A (AU). B x-axis: FSC-H (AU), y-axis: SSC-H (AU). The images are obtained using imaging flow cytometry. They show morphology of the events associated with the data point.

In the non-uniform population, MCDE represent a substantial 31.8% of the population. Here, parameter pairs widely differed in their resolving power (Fig Z in S1 File). The three best-performing pairs were SSC-A – FSC-W, SSC-A – SSC-W, and SSC-W – SSC-H including 1.42 ± 0.95%, 1.59 ± 0.87% and 1.60 ± 0.73% false-positive events, respectively (Fig 2 A and B). The worst performing pair was FSC-A – FSC-H, including 33.2 ± 6.46% false-positive events.

In general, we could see that all parameter pairs that included SSC-A unequivocally allowed specific gating in a high multi-core artefact scenario (on average 2.34 ± 0.75% false positives over all pairs, Fig AA in S1 File). The amount of side scatter increases with an object’s internal complexity [22]. Since the main morphological difference between oil droplets, SCDE and MCDE can be attributed to internal complexity of the objects, it is logical that this parameter has a high resolving power.

In conclusion, SCDE can be robustly identified and gated with high precision, independent of MCDE presence. We saw that SSC-A-based gating performed best of all scatter-based parameters. Integration of DTE with FACS therefore mitigates the impact of non-uniform DTE populations by allowing exclusion of DTE-induced artefacts.

### 3. Droplet-templated emulsification maintains a high core emulsion monodispersity, comparable to chip-based emulsification

To further assess the feasibility of replacing chip-based emulsification with DTE, we compared SCDE produced by these two methods. We hypothesized that the dominant source of extra variability between on-chip methods and DTE stems from a potentially more variable morphology of SCDE (i.e., more variable core emulsions and more variable oil layer thickness) due to less control during emulsification in DTE compared to on-chip methods. To study this, we compared morphology and the influence of morphological differences on fluorescent signal using imaging flow cytometry.

**Fig 3:**
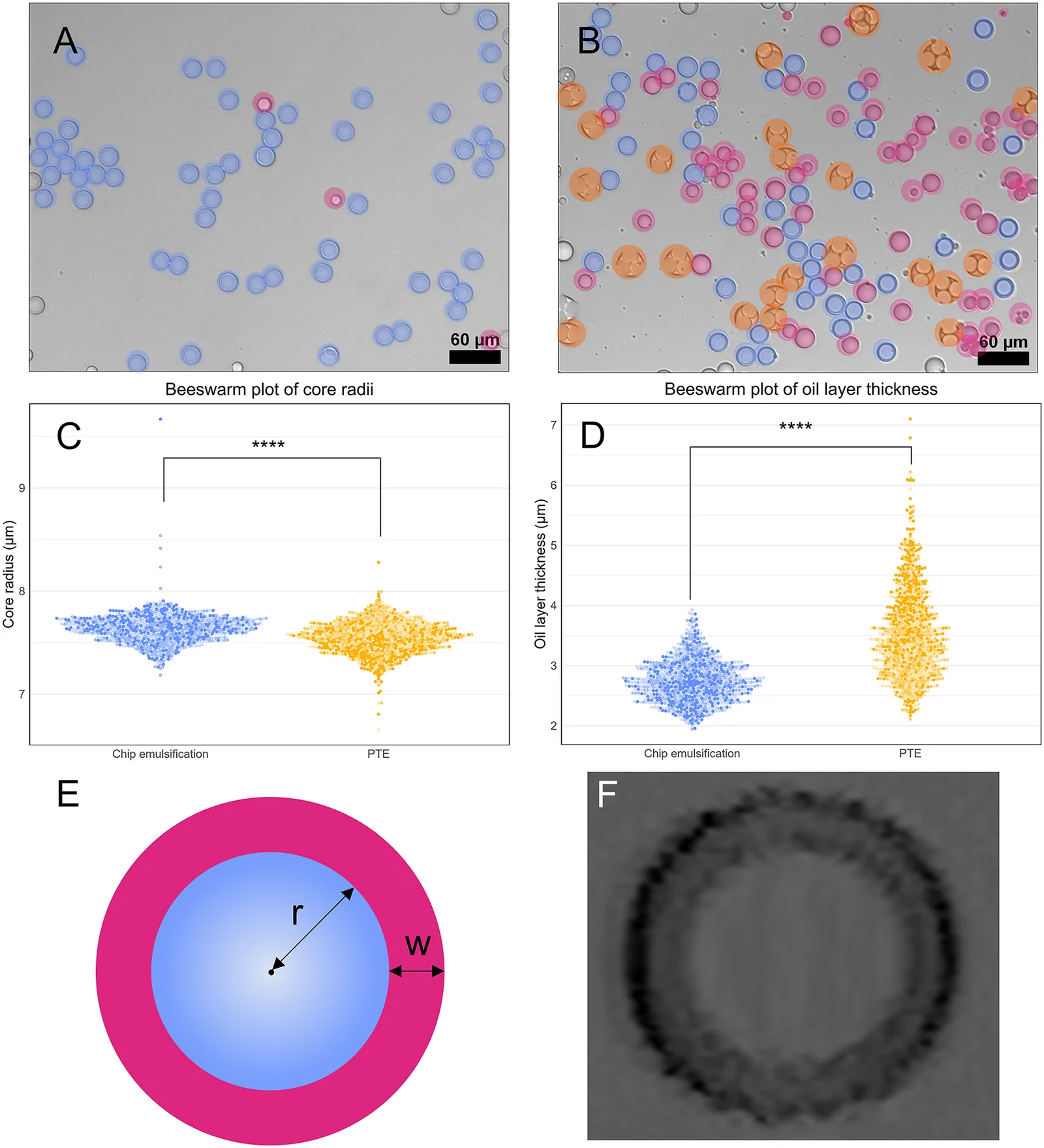
Comparison of chip-made and droplet-templated emulsification–generated single-core double emulsions. A Bright-field microscopy image of a chip-made double emulsion population. Objects are coloured to better distinguish different emulsion morphologies. Blue objects are single-core double emulsions (SCDE); red objects are oil droplets. The white scale bar is 60 µm. B Bright-field microscopy image of a DTE-made double emulsion population. Objects are coloured to better distinguish different emulsion morphologies. Blue objects are SCDE; orange objects are multi-core double emulsions; red objects are oil droplets. The white scale bar is 60 µm. C Beeswarm plot of the core radii of chip-made and DTE-made SCDE (each condition consists of three technical replicates, with n = 306 droplets). D Beeswarm plot of the oil layer thickness of chip-made and DTE-made SCDE (n = 306). E schematic figure of a SCDE, the central blue circle represents the aqueous core emulsion; the surrounding orange layer represents the oil layer. r is the radius of the core emulsion; w is the width of the oil layer. F imaging flow cytometry image of a SCDE. The light region in the center is the core emulsion. The darker layer around it is the oil layer.

Looking only at the desired emulsions (SCDE), we could see that DTE yielded SCDE with core emulsions that presented a similar radius distribution to chip-based emulsification (average r_chip_ = 7.6 ± 0.26 µm and r_DTE_ = 7.6 ± 0.28 µm, 3 technical replicates, n = 306 each, Fig 3C). The core emulsion radii were slightly more variable as shown by the coefficient of variation (CV) (CV_chip_ = 3.40%, CV_DTE_ = 3.74%). A bootstrap analysis revealed a 95% confidence interval from 0.075 µm to 0.104 µm when looking at the difference in means of core emulsion radii between the two methods (p < 10^−4^, two-sided).

The oil shell thickness of SCDE was larger and more variable in DTE (average w_chip_ = 2.7 ± 0.35 µm and w_DTE_ = 3.5 ± 0.76 µm, Fig 3D). The bootstrap analysis of the difference between means between the two methods revealed a 95% confidence interval from 0.74 µm to 0.85 µm (p < 10^−4^, two-sided).

In the context of fluorescence-based assays, the higher oil layer variability in DTE could increase the variability of the fluorescent signal through refraction or other optical phenomena. To test this, we performed a correlation analysis to see if there was a significant correlation between the fluorescent signal coming from the core emulsion (Cy5-A fluorescent signal intensity) and oil layer thickness in DTE. The analysis revealed a Spearman’s ρ of 0.08, indicating a weak positive correlation between the oil layer thickness and fluorescent signal (p = 0.01). Despite the statistical significance, the weak correlation suggests that the practical relevance of this correlation is limited in the range considered (Fig GG in S1 File). Calibration data for size measurements obtained with this technique are provided in S1 File (R² = 0.9997, Fig JJ).

In conclusion, SCDE formed by DTE are uniform, albeit with increased variability compared to chip-based emulsification, predominantly in the oil layer. Although differences in core radii and oil shell thickness are observed, our data suggest that they do not materially influence assay readout under the conditions studied. This supports the use of DTE as a practical bulk alternative to microfluidic methods for a range of screening applications.

### 4. DE-SWIRL successfully sorts and isolates bacteria from microdroplets

**Fig 4:**
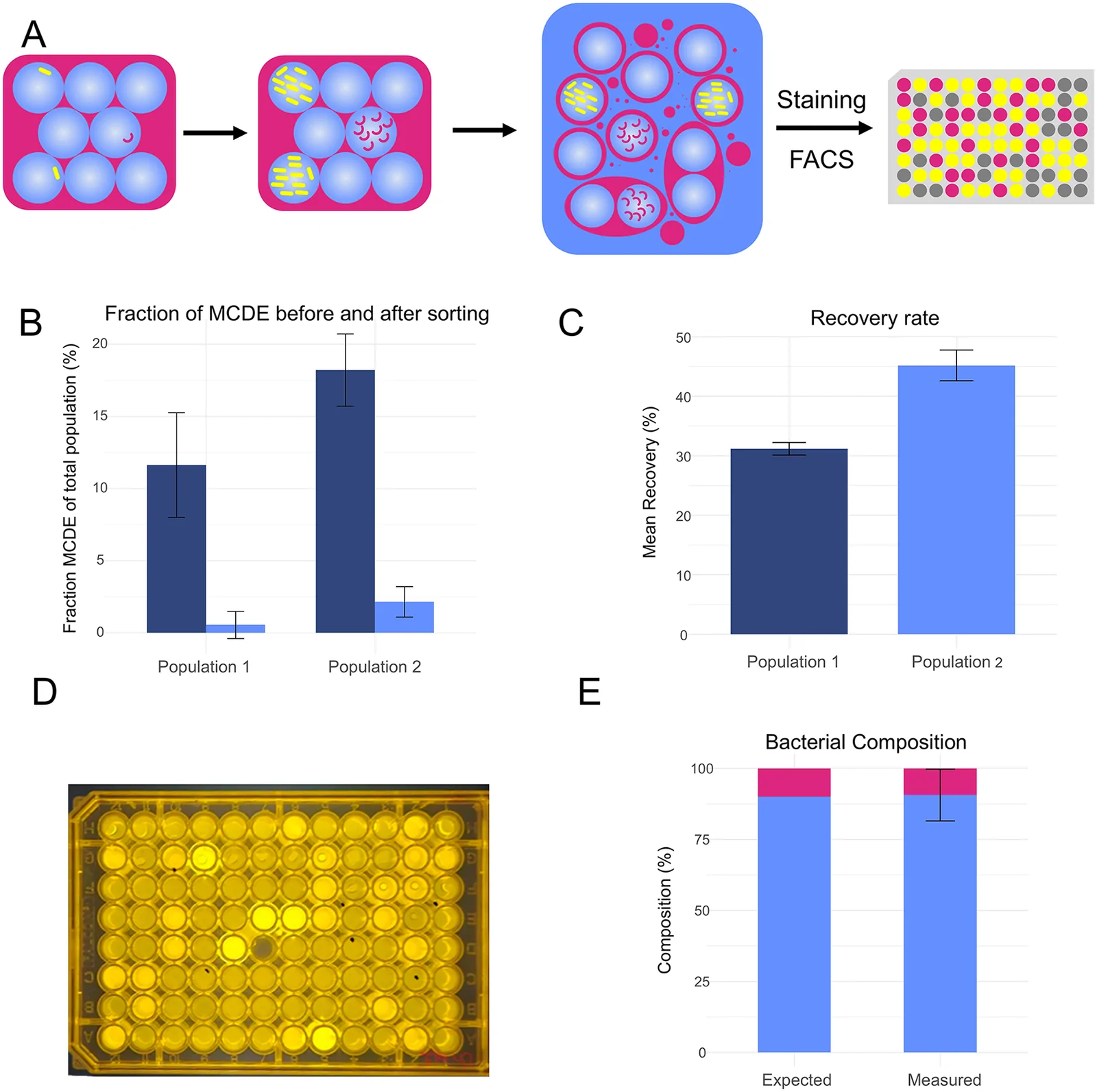
Isolation and recovery of bacterial microcolonies using droplet-templated emulsification and FACS. A Schematic representation of the workflow: two species of bacteria (here Comamonas nitrativorans 23310 (magenta crescent) and Escherichia coli MG1655 Y3 (yellow rod)) are encapsulated in single emulsions at a single-cell level. They are incubated until microcolonies are formed. The single emulsions are emulsified into double emulsions, after which their biomass is stained with SYTO17 (see Materials and methods). Biomass-positive single-core double emulsions are sorted into a 96-well plate and grown. Double emulsion buffer: dark blue, single emulsions: light blue gradient, oil: red. B Barplots showing the mean fraction of multi-core double emulsions before and after sorting of single-core double emulsions. The workflow was performed in two biological replicates (Population 1 and 2). Error bars represent standard deviation (n = 3 technical replicates). C Barplots showing the mean recovery of SCDE after sorting. Error bars represent standard deviation (n = 3). D A photograph of a 96-well plate on a blue transilluminator plate showing grown colonies. The 96-well plate displays three distinct fluorescence intensity levels: bright yellow indicates *E. coli* Y3 expressing YFP, intermediate levels correspond to *C. nitrativorans* 23310 (non-fluorescently tagged), and the lowest fluorescence levels represent wells containing only medium. E Barplots showing the composition of the original bacterial suspension that was encapsulated in single emulsions and the mean species composition of the sorted 96-well plates (n = 2, population 1 and 2). Blue colour represents the fraction of *C. nitrativorans* 23310; red represents the fraction of *E. coli*.

In the previous sections, we demonstrated that despite introducing MCDE, DTE produces high-quality SCDE (monodisperse core emulsions) that can be easily gated with FACS. Here, we performed a full DE-SWIRL workflow. We prepared a synthetic community consisting of 90% *C. nitrativorans* 23310 [23] – a slow-growing bacterium – and 10% *E. coli* MG1655 Y3 [24] – a fast-growing bacterium – and incubated them in single emulsions in biological duplicate (Fig 4A). In short, single cells were encapsulated in single emulsions. We did this by making a cell suspension of 10^7^ cells/mL and flowing it, together with an immiscible oil into a microfluidic chip that makes ~10 pL single emulsions, which yields on average 0.1 cells/single emulsion (=10^7^ cells/mL*(10 pL/single emulsion*10^−9^ mL/pL)). This single emulsion population is then collected and incubated. During incubation, the cells clonally expand into microcolonies within single emulsions. After incubation, we performed DTE followed by FACS. We determined sorting purity (i.e., the ratio of the observed number of SCDE over the total number of sorted emulsions) and sorting recovery (i.e., the observed number of SCDE over the theoretically sorted number of SCDE) (see S1 File: Detailed DE-SWIRL protocol) by sorting SCDE and determining the number of sorted events and their composition (i.e., SCDE or MCDE). To extract microcolonies, we stained biomass inside of the double emulsions with a fluorescent dye by adding SYTO17 to the double emulsion buffer (final concentration 5 µM) and incubating at 37 °C for 30 minutes. Following this staining step, double emulsions were washed three times to remove excess dye. We sorted the droplets with the top 5% biomass signal into growth medium. This threshold was used to sort double emulsions containing fully grown microcolonies; oil droplets were sorted into growth medium as a negative control.

The two DTE populations were non-uniform, with the MCDE fraction being 11.63 ± 3.63% and 18.20 ± 2.51%, respectively. We used the best-performing gating pair of our previously validated gating approach (SSC-A/FSC-W) to select only SCDE and sorted 200 events. MCDE were almost completely eliminated in the sorted fraction (SCDE fraction: 99.45 ± 0.95% and 97.85 ± 1.06%, Fig KK and Fig LL in S1 File), showing high specificity for the gated population. These values are in the same range as purity values observed after conventional cell or particle sorting [25–27]. Non-stringent gating leads to less pure sorts (Fig MM in S1 File). Sorting recovery, which quantifies the efficiency with which desired events are collected post-sorting, was measured for SCDE at 31.17 ± 1.04% and 45.17 ± 2.57%.

FACS revealed clearly discernible populations of biomass-containing and empty droplets, with 7.38% and 4.99% positive droplets (Fig NN and Fig OO, in S1 File). These results are supported by microscopy, which yielded slightly higher values of 10.05% and 7.16%, respectively (Fig J and Fig K, in S1 File). The microscopy values, which were approximately 1.4-fold higher, suggest that our biomass staining assay is not as sensitive in detecting droplets with low biomass content. The top 5% biomass-positive droplets were sorted to select fully grown microcolonies. After incubation, 19.8% and 35.4% of the wells had grown (i.e., isolation efficiency). The negative control sort did not show any wells with growth. An inherent property of droplet microfluidic workflows is the compartmentalization of nutrients. This allows slow-growing and fast-growing organisms to be isolated with similar efficiency, in contrast to bulk enrichments in which fast-growing species outcompete slower-growing individuals [28]. Here, we see this property reflected after sorting: on average 91% ± 9% of the slow-growing *C. nitrativorans* was found and 9% ± 9% of the fast-growing *E. coli* was found (Fig 4E), reflecting the ratios seen in the initial population (90% and 10% respectively, for *C. nitrativorans* and *E. coli*), meaning no bias was introduced due to growth rate.

To further validate the applicability of DE-SWIRL beyond synthetic communities, we performed an additional biomass-based screen using a mixed microbial community (hydrogen-oxidizing enrichment). Using SYBR Green staining, we observed clear separation between stained and unstained SCDE during FACS analysis (Fig PP in S1 File). In this experiment, droplets of approximately 20 pL were used. Sorted stained SCDE were successfully recovered as grown cultures in 96-well plates, confirming downstream viability and compatibility with standard cultivation workflows (Fig QQ in S1 File). Together with the preceding workflow, the data demonstrate the flexibility of DE-SWIRL across different droplet-size regimes and different biomass stains.

## Discussion

### DTE produces SCDE of high quality and is rapidly optimized to produce near-uniform (>90% SCDE) populations

Core emulsions are the assay wells of high-throughput functionality screens; variation in the core emulsion volume introduces variability in final readouts. Retaining uniformity of the template single emulsion population is important for minimizing technical variation and distinguishing true biological heterogeneity. The DTE protocol used here largely maintains the uniformity of the template single emulsion population, although with more variable oil layers than chip-based emulsification (Fig 3).

To provide context for the performance of the protocol, we compared the relative increase in CV of core-emulsion diameter with values estimated from previously published studies (Table 2). The present protocol, adapted from Wang et al. (2022), increased the CV by a factor of 1.1 relative to the template single emulsion population, compared with estimated increases of 2.23 for Sukovich et al. (2017) and 3.7 for Lin et al. (2022) (Table 2) [14–16]. These comparisons should be interpreted as semi-quantitative rather than as a direct performance benchmark. Some values were derived from measurements of published images, and only a single image was available for analysis in the referenced studies, whereas the present dataset included multiple replicates. Nevertheless, the magnitude of the observed differences suggests that the protocol described here preserves core-emulsion uniformity comparatively better than those previously published. Details of the estimation procedure are provided in S1 File to allow assessment of the comparison and its associated uncertainty.

**Table 2:** Comparison of emulsion-generation protocols and the resulting variability in core emulsions. Coefficients of variation (CVs, i.e., standard deviation of diameter divided by mean diameter) marked with an asterisk (*) were derived based on published images; calculation details are provided in S1 File. The ratio represents the CV of the core emulsions divided by the CV of the corresponding template single emulsions.

	This work	Sukovich et al. (2017) [14]	Lin et al. (2022) [15]
*Reference of first protocol*	Wang *et al*. (2022) [16]	Sukovich *et al*. 2017 [14]	Sukovich *et al*. 2017 [14]
*Shear force generation*	Vortexing	Vortexing and flicking	Vortexing and flicking
*Variability of template single emulsions (CV)*	3.40%	6.94%*	4.07%
*Variability of core emulsions (CV)*	3.74%	15.47%*	15.10%*
*(CV after DTE)/(CV before DTE)*	1.10	2.23	3.71

The preservation of core uniformity observed with this DTE protocol appears to distinguish it from previously reported approaches (see S1 File for a detailed step-by-step protocol and data repository for video protocol [14,15]). This may reflect better control over shear forces in this protocol. The present protocol specifies a defined vortexing regime and requires formation of a stable, thin film along the vessel wall, promoting more repeatable vortex-flow behavior [16], whereas other protocols use less-defined combinations of vortexing and tube flicking [14,15]. This likely results in a broader and less controlled distribution of shear forces which may therefore increase the frequency of core-breakup events.

In this work, we only made use of microfluidically produced monodisperse single emulsions since monodispersity is important in the context of microbial assays. Producing monodisperse templates requires syringe pumps, a commercially available microfluidic chip, and standard consumables, representing the main equipment requirement of the workflow. Nevertheless, this remains a relatively modest investment compared with fully microfluidic double-emulsion production and provides a practical balance between accessibility, cost, and control over template uniformity (for cost comparison, see Table A in S1 File). Other approaches to generate monodisperse water-in-oil emulsions have been described but were not evaluated in this study; readers are referred to relevant reviews for a broader comparison of available methods [29–31].

During DTE, large double emulsion complexes are randomly broken up into smaller and smaller MCDE. The endpoint of this breakup depends on the shear forces that are exerted during vortexing [16]. These shear forces need to be sufficiently large to break up MCDE into SCDE (see Fig 1 and Fig RR in S1 File) but not so large as to break up SCDE into broken core double emulsions. Based on our results, we hypothesize that the outcome of DTE can be divided into three regimes: the multi-core regime, the single-core regime (Uniformity > 90%) and the broken core regime (Fig S and Fig U in S1 File). In the multi-core regime, MCDE are abundant, an indication that the forces during vortexing were not sufficient to break large fractions of MCDE into SCDE (Fig N and Fig P, in S1 File). Increasing the vortex speed increases the fraction of cores that end up in SCDE, increasing conversion efficiency and population uniformity (Fig O and Fig R in S1 File). This happens up to a critical point where increasing vortex speed starts breaking up cores and starts introducing unwanted broken core double emulsions – the broken core regime. These outcomes can be used as guides for rapid coarse-grained optimization of DTE.

### Near-uniform DTE populations are gated and sorted by FACS

As mentioned before, we did not succeed in obtaining populations with 100% SCDE (Uniformity = 100%). MCDE, although limited in numbers, were present in every population we made (Fig 1B and Fig 3B, and Figs M-W in S1 File). This was also reported by Wang et al. (2022). The consistent appearance of MCDE after vortexing remains unexplained, suggesting random elements in the DTE process. This spurred us to validate a side scatter-based gating strategy, which allows DE-SWIRL to be used despite non-uniform populations.

Double-emulsion FACS sorting achieved high specificity comparable to conventional cell and particle sorting, while recovery was consistent with previously reported values for double-emulsion sorts. Firstly, sorting SCDE showed high specificity: sorts showed similar purity as conventional cell or particle sorting (Fig 4B; 98–99%, [25–27]). Secondly, sorting recoveries were between 30 and 45% (Fig 4C). Typical FACS sort recovery ranges from 50 to 90% depending on cell type and size, with lower sorting recoveries seen for larger particles [32,33]. The lower recovery values we report here are typical for double emulsion sorts due to their size [34–36]. Recovery can be increased by optimizing delay time; in this way sorting recoveries of up to 88% have been reported [35]. In our example (DE-SWIRL of synthetic community), we did not optimize delay time since sorting was highly specific and recovery allowed sufficient throughput to validate DE-SWIRL. However, in high-throughput settings, optimizing delay time is recommended. This allows more efficient isolation of rare events, improves sample use, and reduces total sort time.

On a final note, we want to point out that DTE populations with a large fraction of MCDE could result in less stable sorts. This is especially the case when MCDE are large relative to the FACS nozzle size, causing fanning and an unstable droplet break-off point [21,34,36,37]. To quantify this effect (MCDE-driven sorting errors), we evaluated sorting efficiency and stability as a function of emulsion uniformity using two double-emulsion populations with markedly different MCDE fractions (Fig SS in S1 File). Increasing uniformity from 36.14% to 87.35% resulted in a corresponding increase in sorting efficiency from 68.87 ± 7.02% to 82.0 ± 3.46%, indicating a clear trend toward improved sorting performance with higher uniformity. Although this difference did not reach statistical significance (p = 0.06, Welch two-sample t-test), the lower-uniformity population showed both lower average sorting efficiency and greater variability between measurements. Together, these observations indicate less repeatable and less robust sorting at lower emulsion uniformity, consistent with MCDE-induced fanning and perturbation of the FACS nozzle break-off. Importantly, successful SCDE sorting was still achievable even at low uniformity, demonstrating that MCDE presence does not preclude accurate sorting but instead reduces robustness. Based on these observations, we recommend aiming for uniform populations when possible, to minimize MCDE-driven sorting errors and ensure reproducible operation.

### Droplet size is a critical parameter for DE-SWIRL

Droplet size is a critical parameter for high-throughput functionality screens. It determines the kinetics of biological reactions and the carrying capacity of microcultures. However, droplet size is bounded by technical limits of DTE and FACS in DE-SWIRL. DTE determines the lower limit of droplet size in DE-SWIRL since smaller droplets are more difficult to emulsify into near-uniform populations (Fig 1B). The upper limit of droplet size is expected to be primarily constrained by the FACS nozzle size used, since DTE efficiently converts large droplets to near-uniform populations. A general rule of thumb suggests that the droplet diameter should not exceed one-third of the nozzle diameter to avoid disturbing the FACS stream. On top of this, droplets that are too large relative to the nozzle size are prone to deformation and may break during sorting [38]. Taking these considerations into account, we predict that DE-SWIRL is compatible with droplet sizes from 6 pL up to 50 pL. Although we did not explicitly test the DTE conversion efficiency with large droplet sizes (>24 pL), reported DTE behavior with large droplets (up to 65 pL) does not seem to change, suggesting that conversion efficiency remains consistent across this range [16]. On top of this, sorting of droplets up to 50 pL has been shown [39].

### DE-SWIRL allows high-throughput microbiome screening and sorting of desired events

In this study, we showed that single emulsions with grown microcultures can be turned into double emulsions and sorted out and grown to yield macrocultures (Fig 4D). This capability is important since it allows physiological characterization of (rare) isolates that display a desired trait or function. In this study, our isolation efficiencies were 19.8% and 35.4%, aligning with the only other reported isolation efficiency of 37.5% in [40]. Isolation efficiency after FACS depends on sorting recovery and on the physiological state of microcolonies. Here, factoring in the respective sorting recoveries, we saw that 63.5% and 78.3% of sorted microcolonies started growing.

In this study, up to 44.49 ± 7.93% of 6 pL and 51.67 ± 5.51% of 24 pL single emulsions survived the DTE treatment (Fig 1C). Seeing that around 10^8^ single emulsions can be made in 3 hours, and considering a high loading of 20% of droplets containing a single cell (as determined by Poisson statistics), this means that 10^7^ bacterial cells can be screened using DE-SWIRL. This throughput allows the exhaustive screening of all bacteria residing in 10 mL of seawater [41], one mg of feces [42] or soil [43], or 10 cm² of skin [44]. DE-SWIRL can be applied to virtually any microbiome, being restricted only by (i) the availability of extraction techniques that allow representative sampling of the microbiome members from their environment, and (ii) the ability to disperse the bacteria into individual cells for encapsulation.

We focused on biomass-based readouts in this study, due to their simplicity and ease of interpretation, yielding a robust starting point for validating the workflow. We expect any published fluorescent assay that was performed with on-chip double emulsions to be directly adaptable to DE-SWIRL (such as [10,40,45]). Any assay developed for single emulsions (such as [11,46–48]) could, in principle, be adapted to double emulsions, but will require additional optimization due to differences in ‘leakiness’ between single and double emulsions [49,50].

We included a detailed DE-SWIRL protocol in S1 File, which together with the data reported and the video protocol in the data repository, maximizes implementability. The total cost of implementation is estimated to be around US$ 3,000 (syringe pumps being the main contributor to cost, see Table A in S1 File).

## Conclusion

In this study, we validated DE-SWIRL, a readily accessible workflow that allows single-cell functionality screens of microbiomes with limited financial investment (~US$ 3,000, required to produce uniform template single emulsions). We showed that DTE produces near-uniform double emulsion populations for sortable droplet sizes. We compared the quality of DTE double emulsions to chip-based double emulsions and saw that while DTE did introduce variability in core emulsions compared to on-chip methods, this variability was not substantial. We showed that in near-uniform conditions, DTE and FACS are compatible, allowing accurate analysis and sorting of double emulsions. By facilitating the exploration of single-cell variability, DE-SWIRL addresses a critical methodological gap for microbiology and microbial ecology. Going forward, we expect DE-SWIRL to enable more accessible single-cell functionality screening, leading to new insights into the heterogeneity of microbial populations.

## Supporting information

S1 File Supporting Information (PDF)

## Abbreviation:

SCDE: single-core double emulsion
MCDE: multi-core double emulsion
DTE: droplet-templated emulsification
FACS: fluorescence-activated cell sorting
DE-SWIRL: Double Emulsion-Sorting Workflow with sImple, Rapid emuLsification
CV: coefficient of variation
