# Supplementary material for: Accessible, robust ultrahigh-throughput microbiome analysis via integration of uniform droplet-templated emulsification and FACS
Source: PLoS One. 2026 Sep 9;21(9):e0356887. doi: 10.1371/journal.pone.0356887 (PMC13557392; doi:10.1371/journal.pone.0356887)
Supplement: Supplementary file 1 [file pone.0356887.s001.pdf]

Accessible, robust ultrahigh-throughput microbiome analysis via integration of uniform droplet-templated emulsification and FACS - Electronic supplementary information

Supplementary information - Acronyms

SCDE: single-core double emulsion

MCDE: multi-core double emulsion

DTE: droplet-templated emulsification

FACS: fluorescence-activated cell sorting

DE-SWIRL: Double Emulsion-Sorting Workflow with sImple, Rapid emuLsification

(i)FCM: (imaging) flow cytometry

CV: coefficient of variation

sCy5: sulfo-Cyanine5 carboxylic acid

Supplementary information - Results

Detailed DE-SWIRL protocol

Single emulsion production

Materials: PTFE tubing, HPLC fittings, commercially available chip, HFE7500, fluorosurfactant, syringe pumps, 1 mL syringe, aqueous phase.

As commercial chip suppliers we recommend Darwin microfluidics which deliver flow focusing droplet generators that specify target droplet sizes. For 1/16” OD tubing, inlet holes with 1 mm diameter are suited. We recommend World Precision Instruments AL-1000 as entry level syringe pumps.

1. Set up two syringe pumps and load 1 mL syringes with 1) bacterial suspension and desired dyes or bioreporters and 2) filtered HFE7500 + desired fluorosurfactant.
2. Connect syringes to PTFE tubing (1/16” OD, 0.3 mm ID, Deutsch & Neumann, Germany) using Luer lock-HPLC fittings (IDEX Health and Science, USA) or a syringe needle.
3. Connect the tubing to the chip. To collect the produced droplets, connect a piece of tubing at the outlet of the chip to an Eppendorf tube.
4. Start filling the chip with oil by running oil phase at 300 µL/hr.
5. Start droplet production by running the aqueous phase syringe pump at 250 µL/hr (keep oil running at 300 µL/hr). Observe the start of the droplet generation at the outlet using a stereomicroscope.
6. After droplet production has started, wait 5 minutes for the droplet size to stabilize.
7. After 5 minutes start collecting droplets in a fresh Eppendorf tube. Monodisperse droplets will be formed with cells encapsulated at single-cell level.

Double emulsion production using DTE

For a video protocol of steps 7-12 see the supplementary data of this paper.

Materials: Double emulsion buffer: 2w% Tween20 in 1xPBS (adjust osmotic strength so that it is isotonic to the single emulsion aqueous phase used), Benchtop vortex (we used Analog Vortex Mixer (VWR) in all our trails), 50 mL conical base centrifuge tube, Kimtech precision wipe.

1. Aspirate the desired amount of creamed single emulsions using a micropipette.
2. Wipe the pipette tip with a clean tissue (Kimtech) to remove excess emulsion.
3. Add 2 mL of double emulsion buffer (i.e. buffer volume) to a 50 mL conical base centrifuge tube.
4. Submerge the tip of the micropipette to the bottom of the centrifuge tube.
5. Slowly press the plunger to release the single emulsions into the buffer. Be careful to not add air.
6. Secure the centrifuge tube in a vortex mixer that is turned off. Put the vortex mixer at the lowest RPM.
7. Turn the vortex mixer on.
8. Gradually increase the vortex speed by turning the knob while securing the centrifuge tube in a vertical position.
9. Observe the formation of a thin film along the wall of the tube. The film should show no repeated up and down movement. If such instability occurs, go back to lowest rpm, press down on the vortex to mechanically slow it down, then gradually release to increase the speed. Keep centrifuge tube vertical to avoid destabilizing film.
10. Once a stable film is achieved, continue vortexing the mixture at the set speed for 15 seconds.
11. Monitor the stability of the film throughout the emulsification process to ensure consistency.
12. Turn off vortex after 15 seconds.

13. Look at the produced DTEDTE-population using a microscope to grade uniformity. Count double emulsions in a microwell to establish conversion efficiency if desired (see Materials and Methods)

Goal: Aim for >90% SCDE

Troubleshooting:

Issue	Suggested action(s)
>10% multicore double emulsions (Fig. N)	1. Increase vortexing speed<br>2. Increase recipient diameter
Presence of broken core double emulsions (Fig. U)	1. Decrease vortexing speed<br>2. Decrease recipient diameter
Liquid film does not stabilize	1. Use more emulsion buffer<br>2. Keep falcon vertical without moving it during vortexing

FACS-sorting

Materials: Double emulsion buffer: 2w% Tween20 in 1xPBS (adjust osmotic strength so that it is isotonic to the single emulsion aqueous phase used), FACS tube

1. Let double emulsions sediment for 5 minutes
2. Remove supernatant, be careful not to disturb double emulsion pellet.
3. Add fresh double emulsion buffer.
4. Repeat steps 1 and 3 3x in total. Washing steps remove bacteria from double emulsions broken during DTE.
5. Resuspend double emulsions by shaking gently and aspirate desired amount into FACS tubes.
6. Agitate double emulsions by shaking FACS tube, load FACS tube in FACS and run. Ensure the agitation feature is activated to keep the emulsions in suspension.

7. Use SSC-A and FSC-W to properly set the gating for SCDE. Tip: Logarithmic axes are easier to find SCDE population, linear more suited for accurate gating. Use a small sample to adjust the gates as necessary, confirm gate by sorting into double emulsion stabilizing buffer and observing pure SCDE.
8. Start the FACS sorting process, run at low flow rates (1-5), high flow rates shear double emulsions.
9. If sorting recovery is low, optimize the delay time as seen in [1].
10. To break double emulsions after sorting, sort into 1x PBS. The lack of surfactant breaks the emulsions (see Table E), this step replaces the toxic PFO that is used in other protocols

Troubleshooting:

Issue	Suggested action(s)
Low event rate	1. Double emulsions sedimented: stop analysis and agitate tube by hand<br>2. Bad DTE conversion efficiency: decrease total vortexing time to max 15 seconds<br>3. Low concentration, add less emulsion buffer
No clear SCDE population visible	1. High flow rates shear SCDE into broken double emulsions, reduce flow rate to minimum<br>2. Optimize FSC/SSC detector voltage
Low sort recovery	1. Optimize delay time manually as described in [1,2], generally delay time needs to be increased by 0.2 increments starting from the calibrated delay time until a maximum is seen in sort recovery.<br>2. Increase nozzle size<br>3. If no larger nozzles available, perform FACS-optimization following guidelines for large particle sorting by Galbraith et al. [3,4]<br>4. Low uniformity of DTE population is destabilizing FACS sort: increase DTEDTE uniformity

Low sort purity (nominal = 1-2 % contamination[5–7])	1. Readjust gate<br>2. Increase DTE uniformity
Low isolation efficiency	1. Low sort recovery (see above)<br>2. Low efficiency of double emulsion lysis: sort into neat PBS or another physiologically gentle solution (neat: no surfactant). Add culture medium after. We have seen that sorting directly into culture medium (e.g. LB) stabilizes double emulsions, likely due to surfactant effects of amphiphilic molecules such as oligopeptides.[8] This prevents release of bacteria and yields low isolation efficiencies.<br>3. Bacteria are not physiologically able to grow after lysis: make sure microcultures are fresh, preferably exponential phase

DTE detailed instructions:

An unstable film can be identified by the repeated up and down movement of the aqueous phase front. This instability is undesirable as it has been linked to poorer emulsification outcomes in our trials. An unstable film will either stabilize or cease to exist, halting the emulsification process. In the former case, once a stable film is achieved, it is appropriate to proceed with emulsification, starting the timing from the moment the stable film is obtained.

Using a smaller volume of double emulsion buffer makes it more challenging to achieve a stable film. For small volumes, such as 1.5 mL, users are advised to begin at the lowest RPM, gradually increasing the speed by turning the knob. If a stable film is not achieved, pressing down on the vortex at the lowest speed to slow it down mechanically, releasing the force gradually to allow the speed to increase. A volume of 3 mL is generally easier to work with for achieving a stable film. 1.5 ml is close to the minimal workable volume.

(1) Equation used to calculate sorting recovery.

$$FACS\ Recovery\ = \frac{Observed\ sorted\ amount\ of\ particle\ of\ interest}{Theoretically\ sorted\ amount\ of\ particle\ of\ interest}\ (1)$$

(2) Equation used to calculate FACS purity.

$$FACS\ Purity = \frac{Observed\ sorted\ amount\ of\ particle\ of\ interest}{Observed\ sorted\ amount\ of\ particles}\ (2)$$

Results part 1

Table A Main costs to implement DE-SWIRL compared to on-chip workflow. Consumable costs (tubing, syringes, etc) not included since they are a small contributor to total costs of implementation.

Equipment	Cost ($)	DE-SWIRL	On-chip emulsification	Source
Commercial single emulsion chip	200	1	1	https://darwin-microfluidics.com/products/droplet-generator-pdms-different-nozzle-sizes
WPI AL-1000 syringe pump	945.40	2	3	https://www.fishersci.com/shop/products/sgl-syr-infusion-pump-al1000/NC1881156
Double emulsion chip	In-house fabrication	0	1	/
Vortex	345	1	0	https://www.fishersci.com/shop/products/fisher-scientific-variable-speed-mini-vortex-mixer/14955163#?keyword=
Stereomicroscope	544	1	0	https://www.fishersci.com/shop/products/basic-fix-magnification-stereo-microscope-2/p-7145810#?keyword=
Inverted microscope	11465	0	1	https://www.fishersci.be/shop/products/primovert-inverted-microscope-phototube-2/15168229
Chronos 2.1-HD High Speed Camera	6800	0	1	https://www.krontech.ca/product/chronos-2-1-hd-high-speed-camera/
**Total**	/	2979.8 $	21301.2 $	/

Table B Aqueous phase composition and technical details of single emulsion populations.

Single emulsion population	Composition of aqueous phase	Notes	Figure
4 pL droplet population	100 nM sulfo-cyanine5 carboxylic acid (sCy5, Lumiprobe, Germany) in 1x PBS (10 mM phosphate buffer, 2.7 mM potassium chloride and 137 mM sodium chloride, pH 7.4, at 25 °C, Sigma)	Used 10 µm height chip, droplets used in results part 2 and 3.	Fig. G
6 pL droplet population	1 µM sCy5 in 1x PBS	Used 10 µm height chip, droplets used in results part 1. Double emulsions visible in fig 1	Fig. H
24 pL droplet population	1 µM sCy5 in 1x PBS	Used 20 µm height chip, droplets used in results part 1. Double emulsions visible in fig 1	Fig. I
9 pL synthetic community droplets (Population 1)	in LB (Luria/Miller, Roth):<br>• $9*10^6$/mL *Comomonas nitrativorans* 23310<br>• $1*10^6$/mL *Escherichia coli* MG1665 Y3	Used 10 µm height chip, droplets used in results part 4. Syringes were cooled with ice pack to slow down bacterial growth during droplet making.	Fig. J
7 pL synthetic community droplets (Population 2)	in LB:<br>• $9*10^6$/mL *C. nitrativorans* 23310<br>• $1*10^6$/mL *E. coli* MG1665 Y3	Used 10 µm height chip, droplets used in results part 4. Syringes were cooled with ice pack to slow down bacterial growth during droplet making.	Fig. K

Table C Summary of double emulsion production parameters and corresponding outcomes. This table presents the various parameters tested for double emulsion production, along with their quantitative outcomes (uniformity and conversion efficiency) and qualitative outcomes (figures and observational notes).

Single emulsion population used	Vortex speed (rpm)	Vortex volume (mL)	Volume of single emulsions (µL)	Figure	Regime	Conversion efficiency (%)	Uniformity (%SCDE)	Notes[a]
4 pL droplet population	2500	1.5	15	Fig.3 B, Fig. M	Multicore	/	68.20 ± 1.30	Results part 2, results part 3
6 pL droplet population	300	1.5	15	Fig.1, Fig. N	Multicore	5.11 ± 1.29	29.85 ± 9.43	Results part 1
6 pL droplet population	2500	1.5	15	Fig.1, Fig. O	Single core/Multicore	44.49 ± 7.93	85.06 ± 3.02	Results part 1
6 pL droplet population	300	4.0	15	Fig.1, Fig. P	Multicore	2.26 ± 0.28	22.33 ± 5.82	Results part 1
6 pL droplet population	2500	4.0	15	Fig.1, Fig. Q	Single core/Multicore	34.38 ± 7.09	85.00 ± 3.38	Results part 1
24 pL droplet population	300	1.5	15	Fig.1, Fig. R	Single core	51.67 ± 5.51	98.03 ± 1.03	Results part 1
24 pL droplet population	2500	1.5	15	Fig.1, Fig. S	Broken core	31.21 ± 7.56	28.01 ± 1.42	Broken cores diminish both uniformity and conversion efficiency, Results part 1
24 pL droplet population	300	4.0	15	Fig.1, Fig. T	Single core/Multicore	49.16 ± 29.27	88.01 ± 3.95	Results part 1
24 pL droplet population	2500	4.0	15	Fig.1, Fig. U	Broken core	25.38 ± 1.42	27.13 ± 6.05	Broken cores diminish uniformity, Results part 1

9 pL synthetic community droplets (Population 1)	2500	1.5	15 µL drops + 5 µL 1w% picosurf in HFE7500	Fig.4, Fig. V, Fig. KK	Single core/Multicore	/	88.37 + 3.63	Results part 4, Excess oil was added to allow osmotic swelling of emulsions in hypoosmotic double emulsion buffer[10] resulting in more background oil droplets
7 pL synthetic community droplets (Population 2)	2500	1.5	15 µL drops + 5 µL 1w% picosurf in HFE7500	Fig.4, Fig. W, Fig. LL	Single core/Multicore	/	81.80 ± 2.51	Same as population above

```
Call:
betareg(formula = PTE_recovery ~ buffer_volume * vortex_speed * droplet_radius, data = experiment_data, link = "logit")

Standardized weighted residuals 2:
    Min      1Q  Median      3Q     Max 
-3.9903 -0.4110 -0.1299  0.2386  3.7967 

Coefficients (mean model with logit link):
                                             Estimate Std. Error z value Pr(>|z|)   
(Intercept)                                -7.518e+00  2.355e+00  -3.193  0.00141 **
buffer_volume                              -5.613e-01  8.195e-01  -0.685  0.49339   
vortex_speed                                3.466e-03  1.117e-03   3.104  0.00191 **
droplet_radius                              4.297e-01  1.411e-01   3.047  0.00231 **
buffer_volume:vortex_speed                  1.164e-04  3.847e-04   0.302  0.76230   
buffer_volume:droplet_radius                2.896e-02  4.875e-02   0.594  0.55248   
vortex_speed:droplet_radius                -2.109e-04  6.930e-05  -3.043  0.00234 **
buffer_volume:vortex_speed:droplet_radius -7.884e-06  2.373e-05  -0.332  0.73971   

Phi coefficients (precision model with identity link):
      Estimate Std. Error z value Pr(>|z|)    
(phi)   23.443      6.777   3.459 0.000542 ***
---
Signif. codes:  0 '***' 0.001 '**' 0.01 '*' 0.05 '.' 0.1 ' ' 1 

Type of estimator: ML (maximum likelihood)
Log-likelihood: 28.52 on 9 Df
Pseudo R-squared: 0.9009
Number of iterations: 37 (BFGS) + 2 (Fisher scoring)
```

Fig. A R output of the beta regression model of DTE conversion efficiency (here parameter called DTE_recovery).

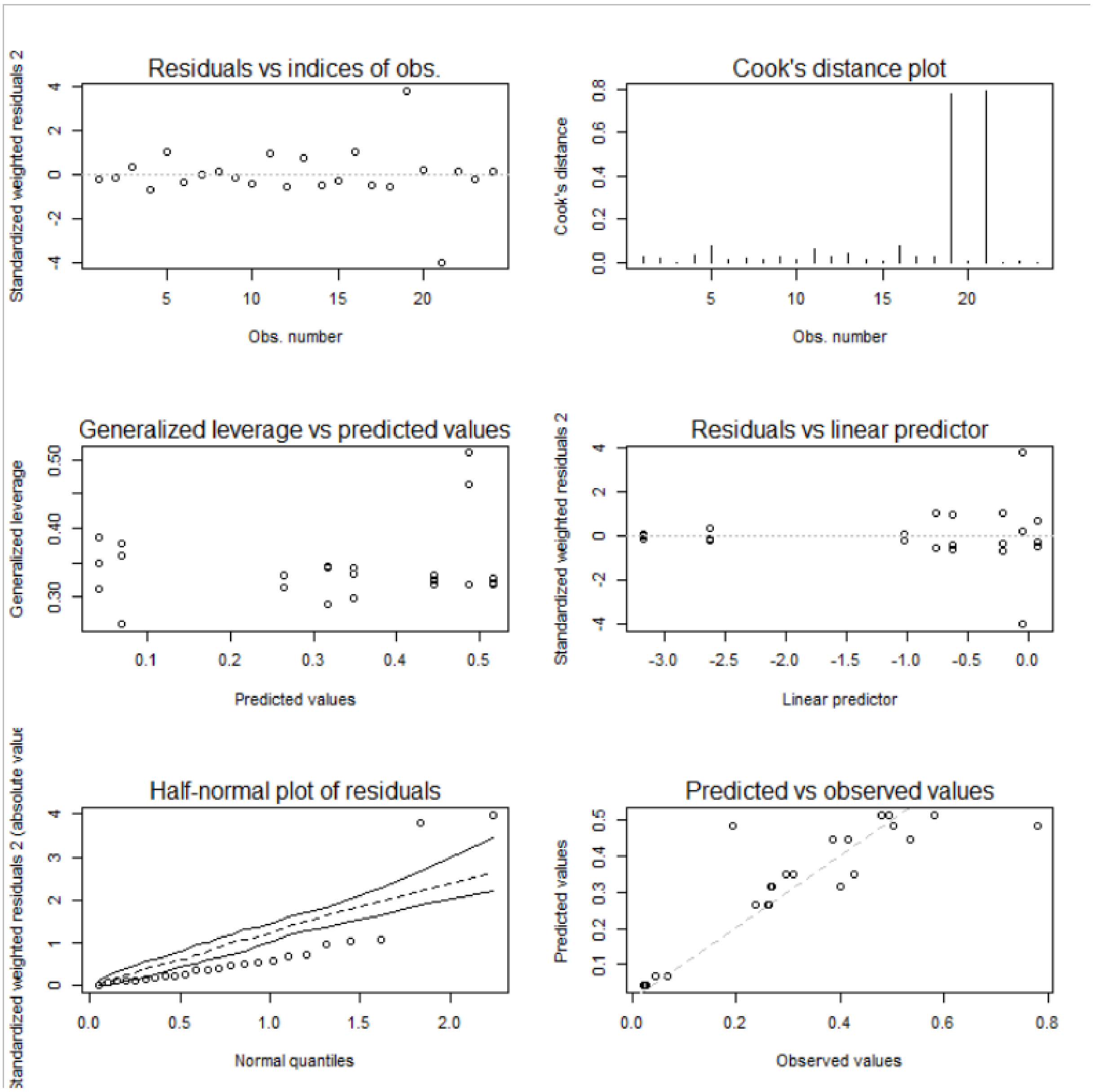

Fig. B Residual plots of beta regression model of DTE conversion efficiency.

```
Call:
betareg(formula = PTE_yield ~ buffer_volume * vortex_speed * droplet_radius, data = experiment_data, link = "logit")

Standardized weighted residuals 2:
    Min      1Q  Median      3Q     Max 
-2.9816 -0.9206  0.1624  0.9659  2.0429 

Coefficients (mean model with logit link):
                                            Estimate Std. Error z value Pr(>|z|)    
(Intercept)                               -1.241e+01  1.335e+00  -9.294  < 2e-16 ***
buffer_volume                              9.341e-01  3.917e-01   2.385  0.01709 *  
vortex_speed                               7.471e-03  6.565e-04  11.380  < 2e-16 ***
droplet_radius                             9.997e-01  1.070e-01   9.346  < 2e-16 ***
buffer_volume:vortex_speed                -3.579e-04  2.009e-04  -1.782  0.07475 .  
buffer_volume:droplet_radius              -9.651e-02  3.029e-02  -3.186  0.00144 ** 
vortex_speed:droplet_radius               -5.587e-04  4.916e-05 -11.364  < 2e-16 ***
buffer_volume:vortex_speed:droplet_radius  3.721e-05  1.450e-05   2.566  0.01028 *  

Phi coefficients (precision model with identity link):
      Estimate Std. Error z value Pr(>|z|)    
(phi)    97.76      28.29   3.456 0.000549 ***
---
Signif. codes:  0 '***' 0.001 '**' 0.01 '*' 0.05 '.' 0.1 ' ' 1 

Type of estimator: ML (maximum likelihood)
Log-likelihood:  46.8 on 9 Df
Pseudo R-squared: 0.9736
Number of iterations: 38 (BFGS) + 2 (Fisher scoring) 
```

Fig. C R output of the beta regression model of DTE uniformity (here parameter called PTE_yield).

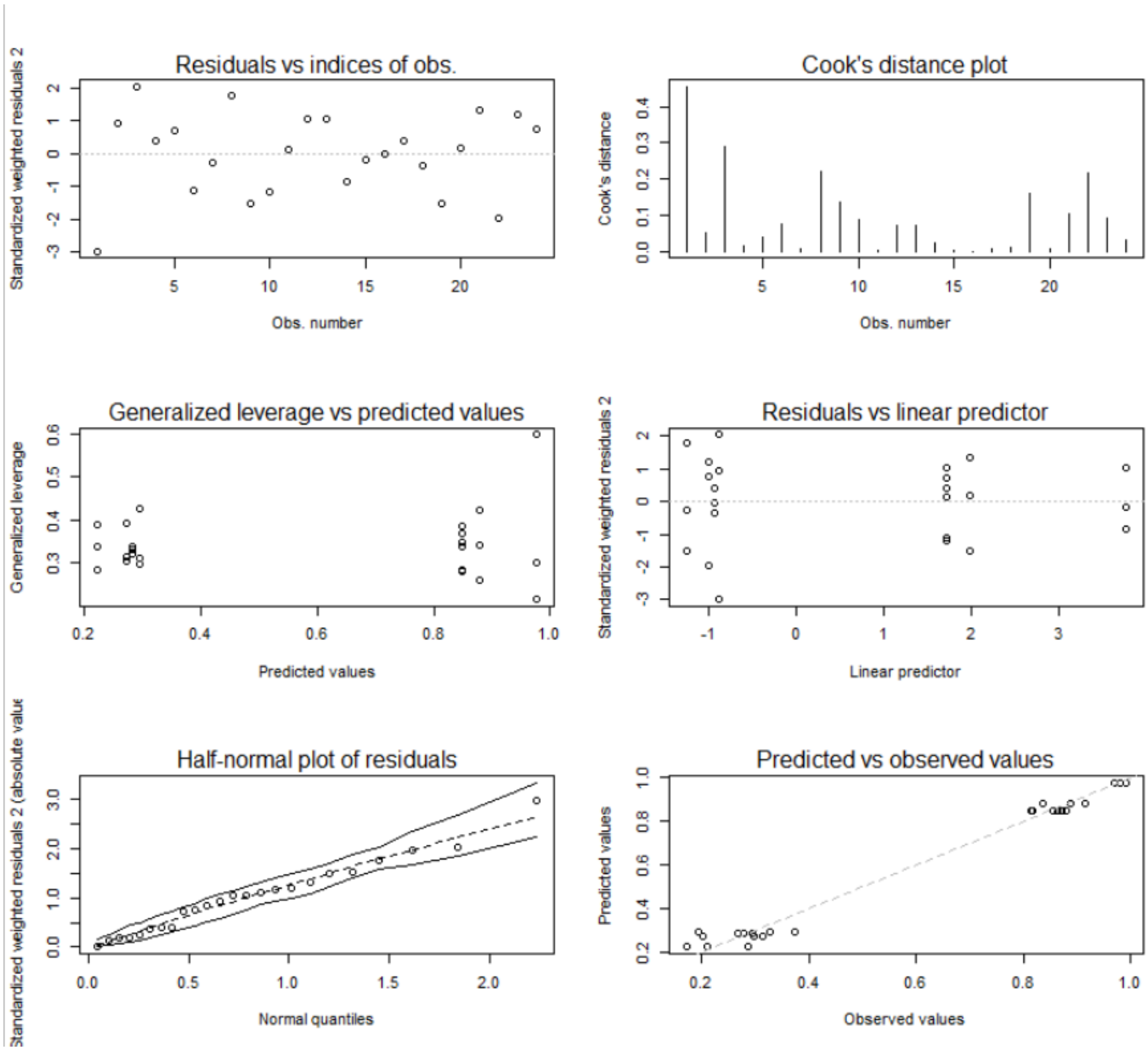

Fig. D Residual plots of beta regression model of DTE uniformity.

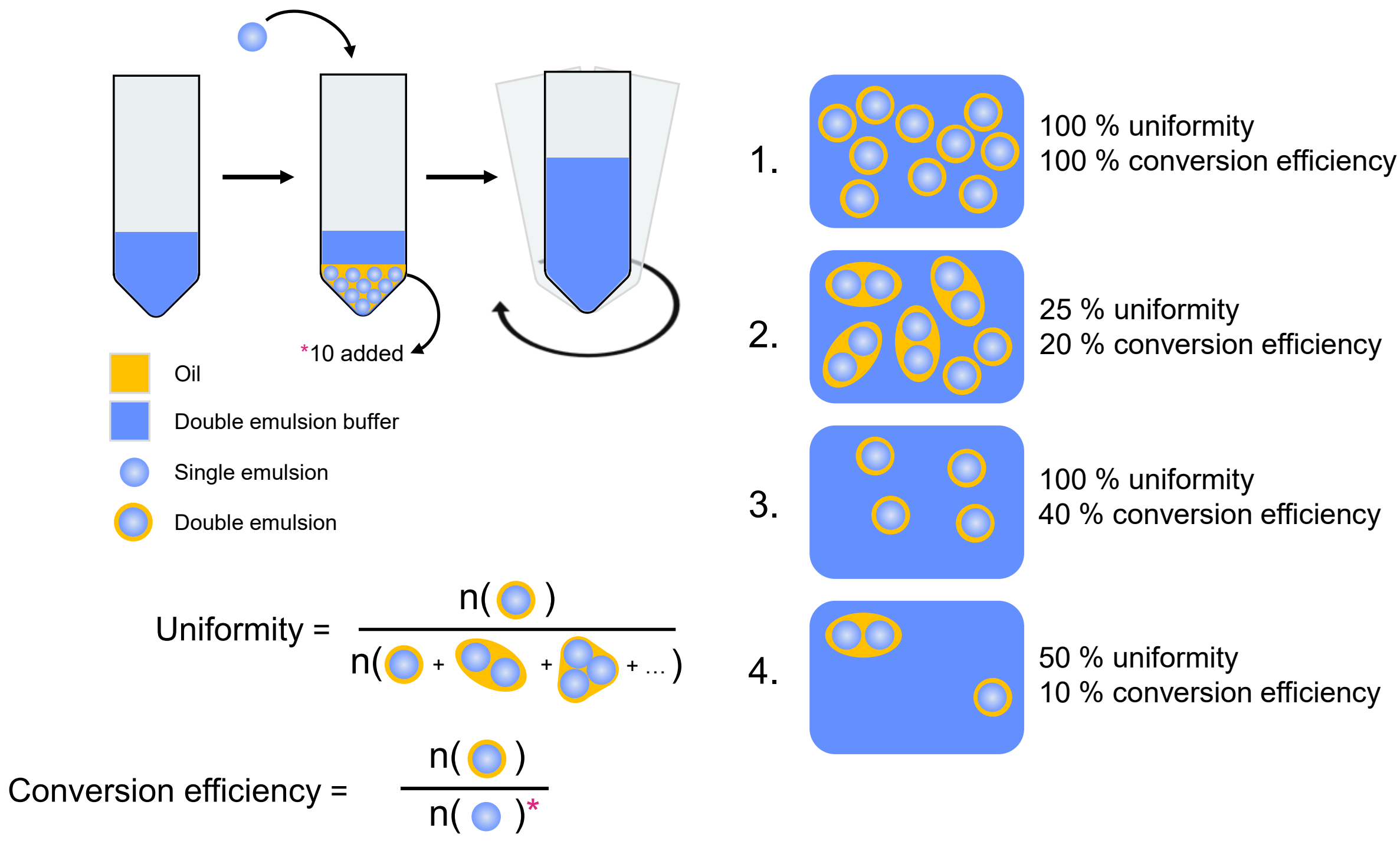

Fig. E Schematic representation of the DTE process and the vortexing outcome parameters, DTE uniformity, and conversion efficiency. Ten single emulsions are added into a recipient, followed by vortexing.[9] The outcomes are as follows: 1. All 10 single emulsions are successfully emulsified into SCDE, resulting in a 100% conversion efficiency (10/10) and a 100% uniformity, as only SCDE are present (10/(10+0)). 2. Only 2 single emulsions form SCDE, leading to a 20% conversion efficiency (2/10). The uniformity is 25%, given that out of 6 double emulsion objects, only 2 are SCDE (2/(4+2)). 3. Four SCDE are formed, equating to a 40% conversion efficiency (4/10). Since all are SCDE, the uniformity is 100%. 4. One single emulsion ends up as SCDE, resulting in a 10% conversion efficiency (1/10). Additionally, 50% of the double emulsions are SCDE (1/(1+1)).

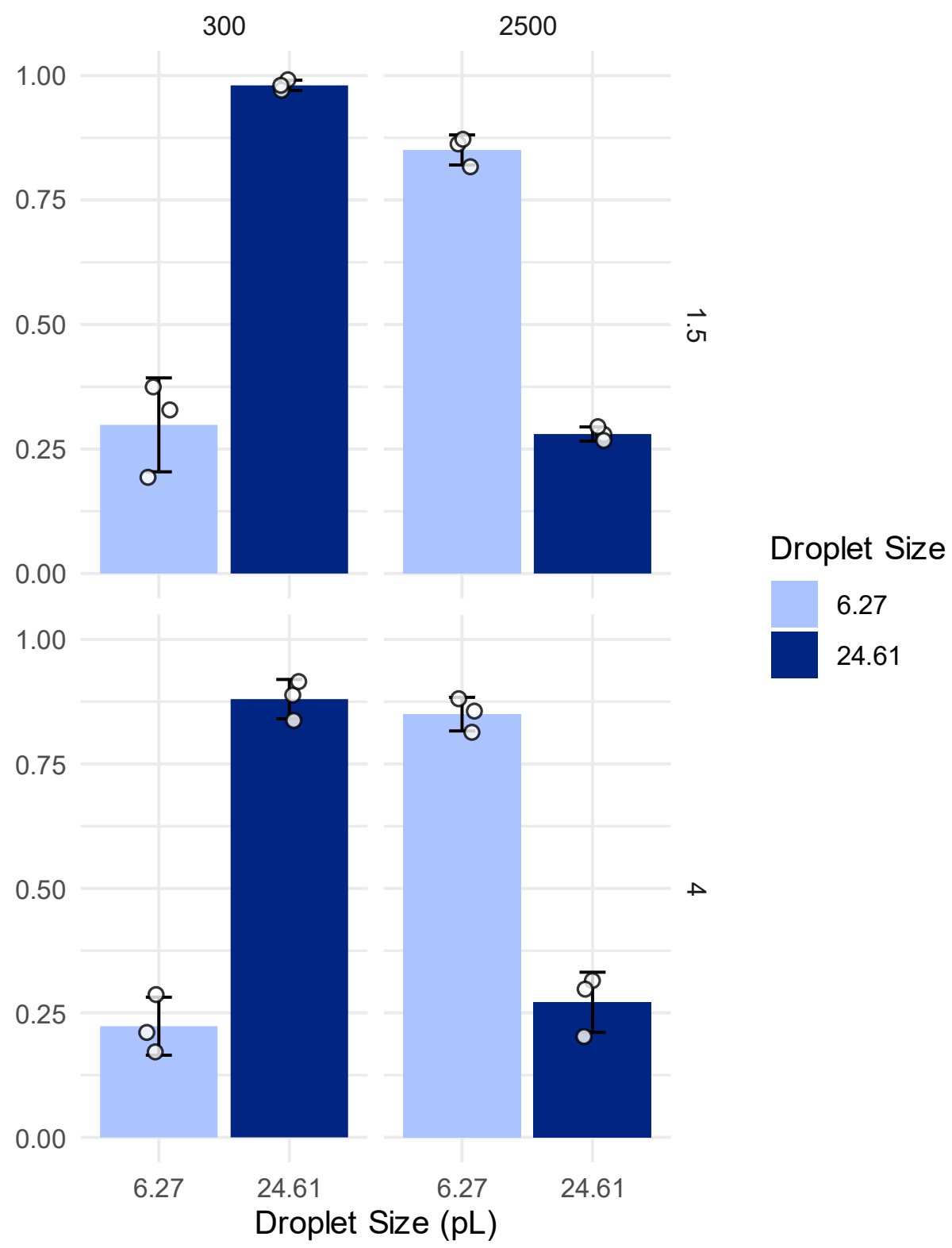

Fig. F *Barplots of mean DTE uniformity of different DTE regimes. Error bars indicate standard deviation (n = 3). The white points are the replicates for each condition.* Rows, *top to bottom: 1.5 mL and 4 mL volume of outer aqueous phase, columns, left to right: 300 rpm and 2500 rpm vortex speed. Maximum uniformity is seen in different DTE regimes depending on droplet size.*

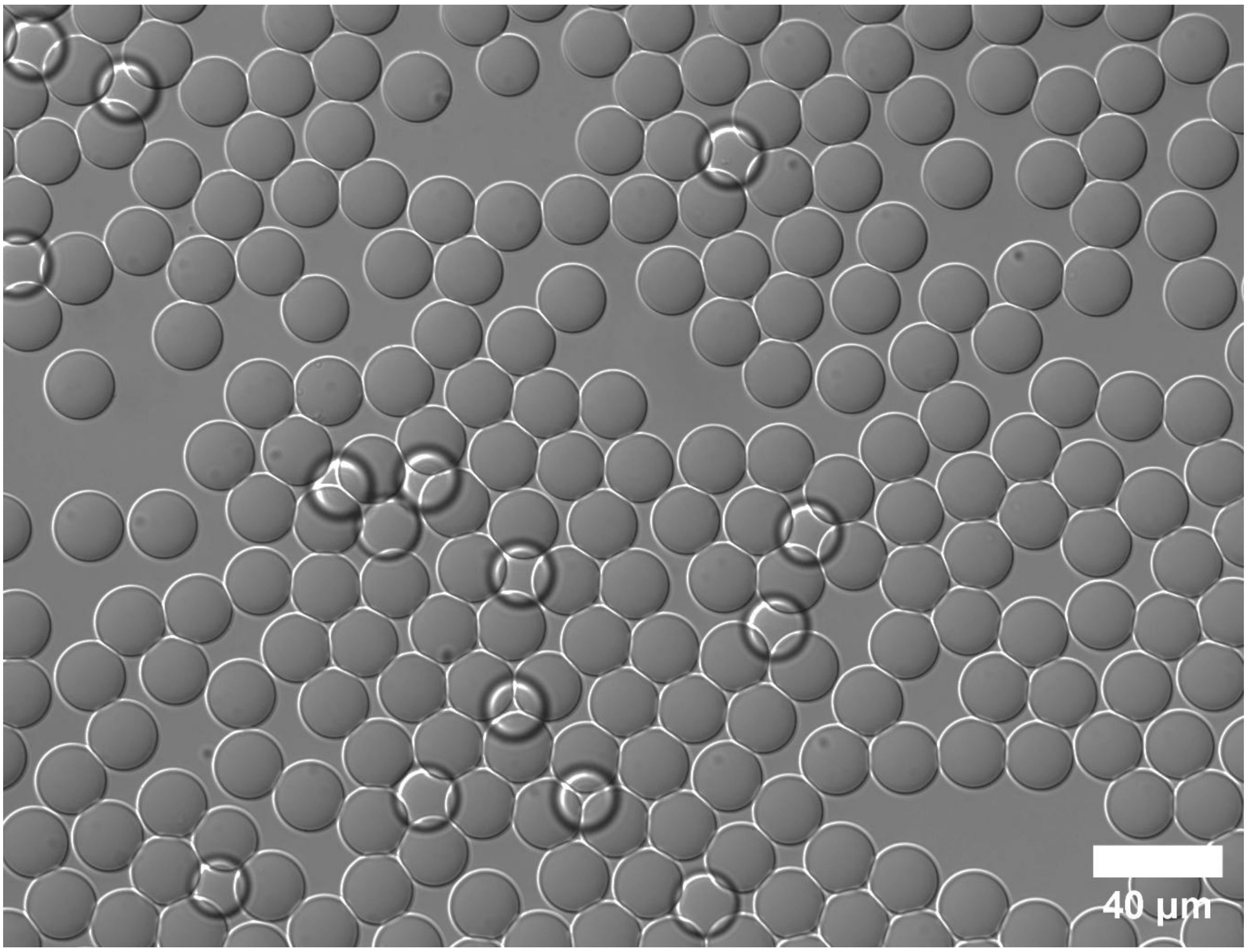

Fig. G Brightfield microscopic image of 4 pL single emulsion population taken with 40x objective.

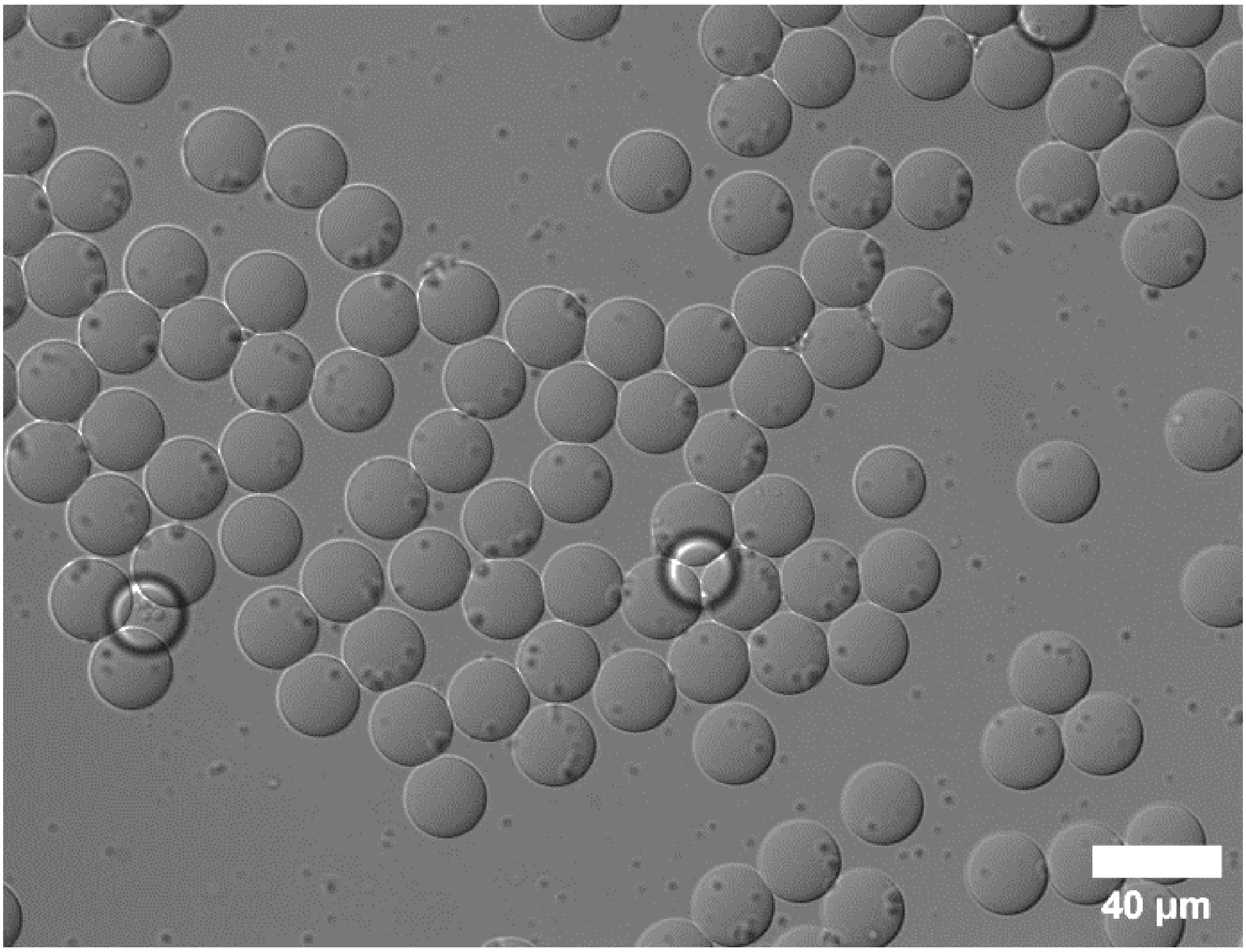

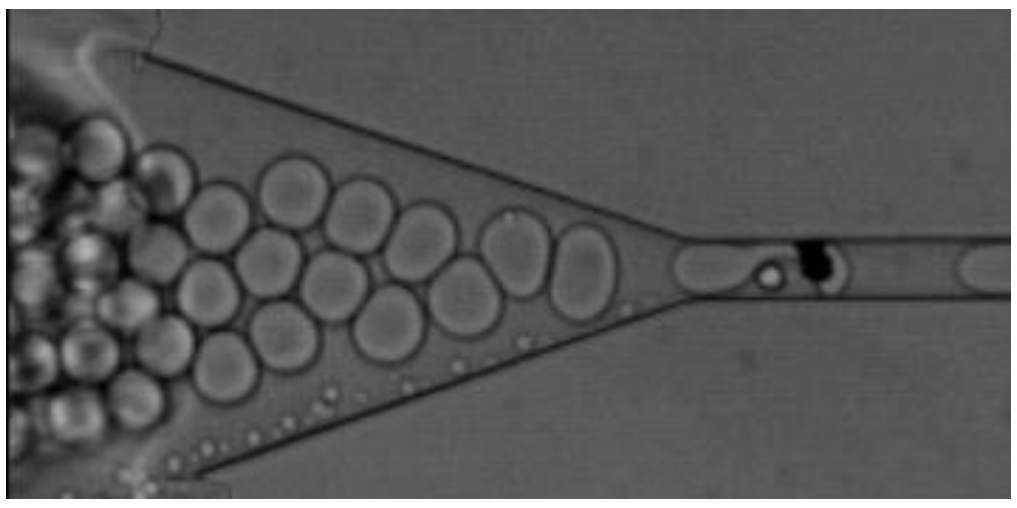

Fig. H Left: Brightfield microscopic image of 6 pL single emulsion population. Black dots are microsatellite droplets formed during droplet generation (right). Taken with 40x objective.

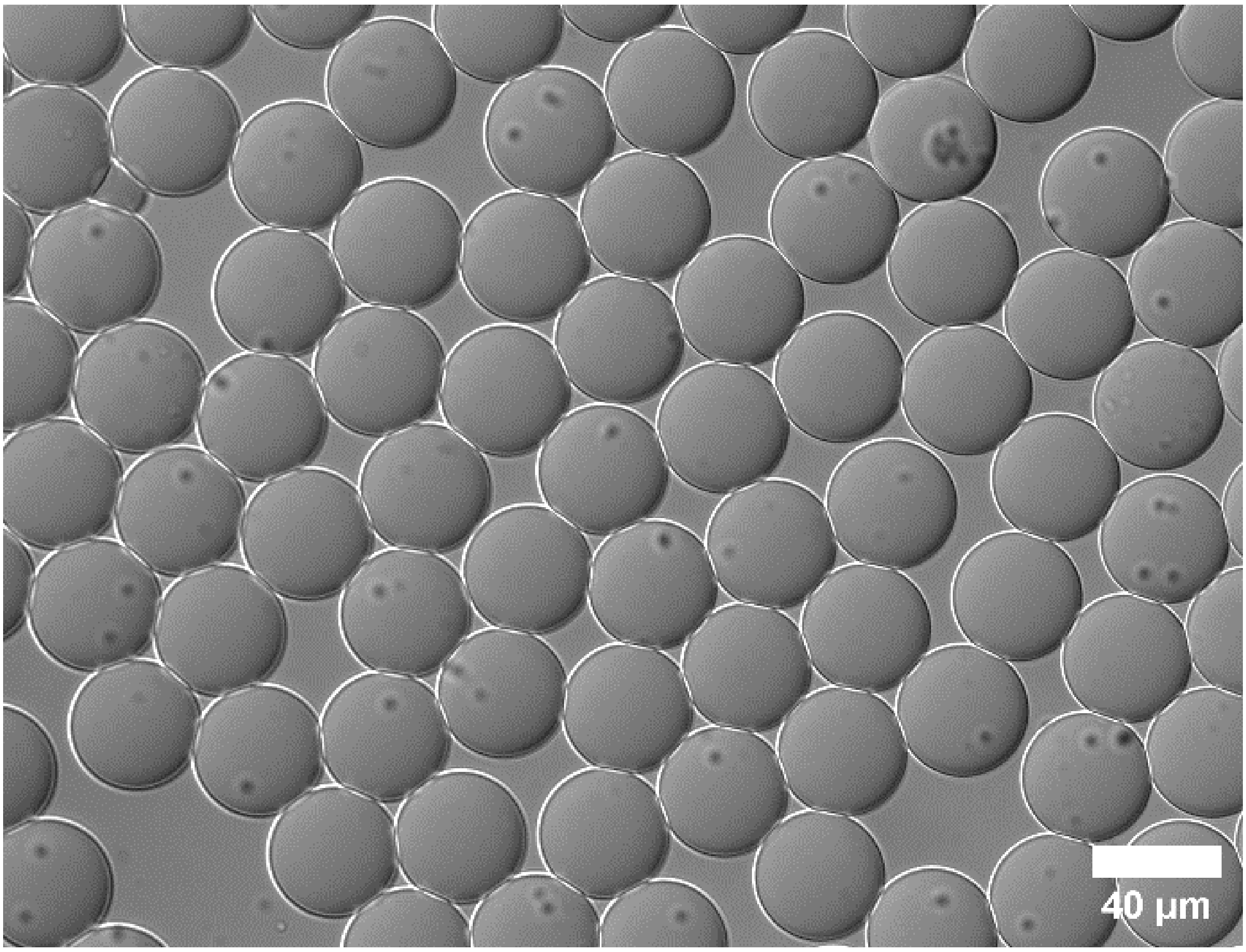

Fig. I Left: Brightfield microscopic image of 24 pL single emulsion population taken with 40x objective. Black dots are microsattelite droplets formed during droplet generation.

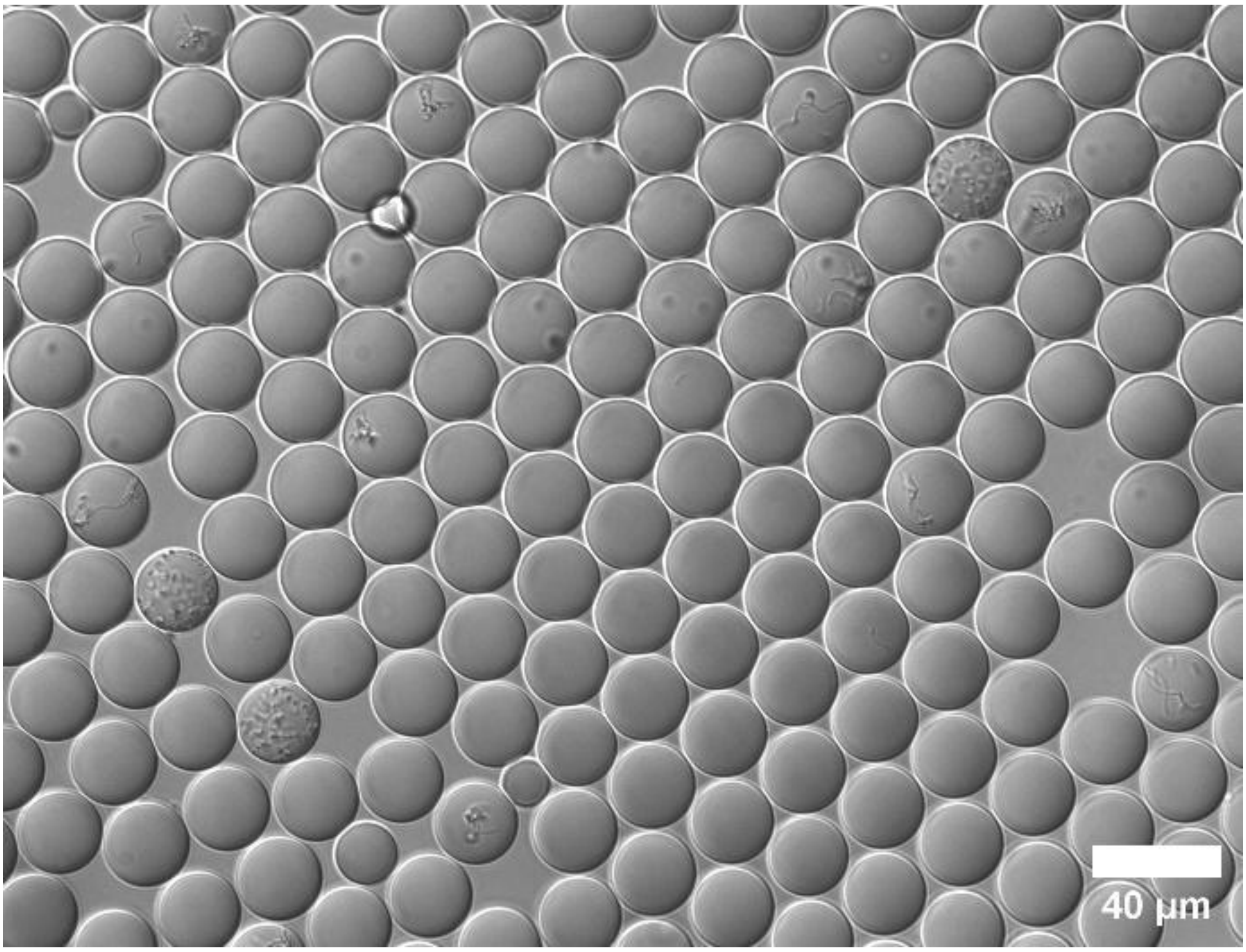

Fig. J Left: Brightfield microscopic image of the 9 pL synthetic community single emulsion population taken with 40x objective (Population 1). The structures inside of the droplets are microcultures of *E. coli* and *C. nitrativorans*.

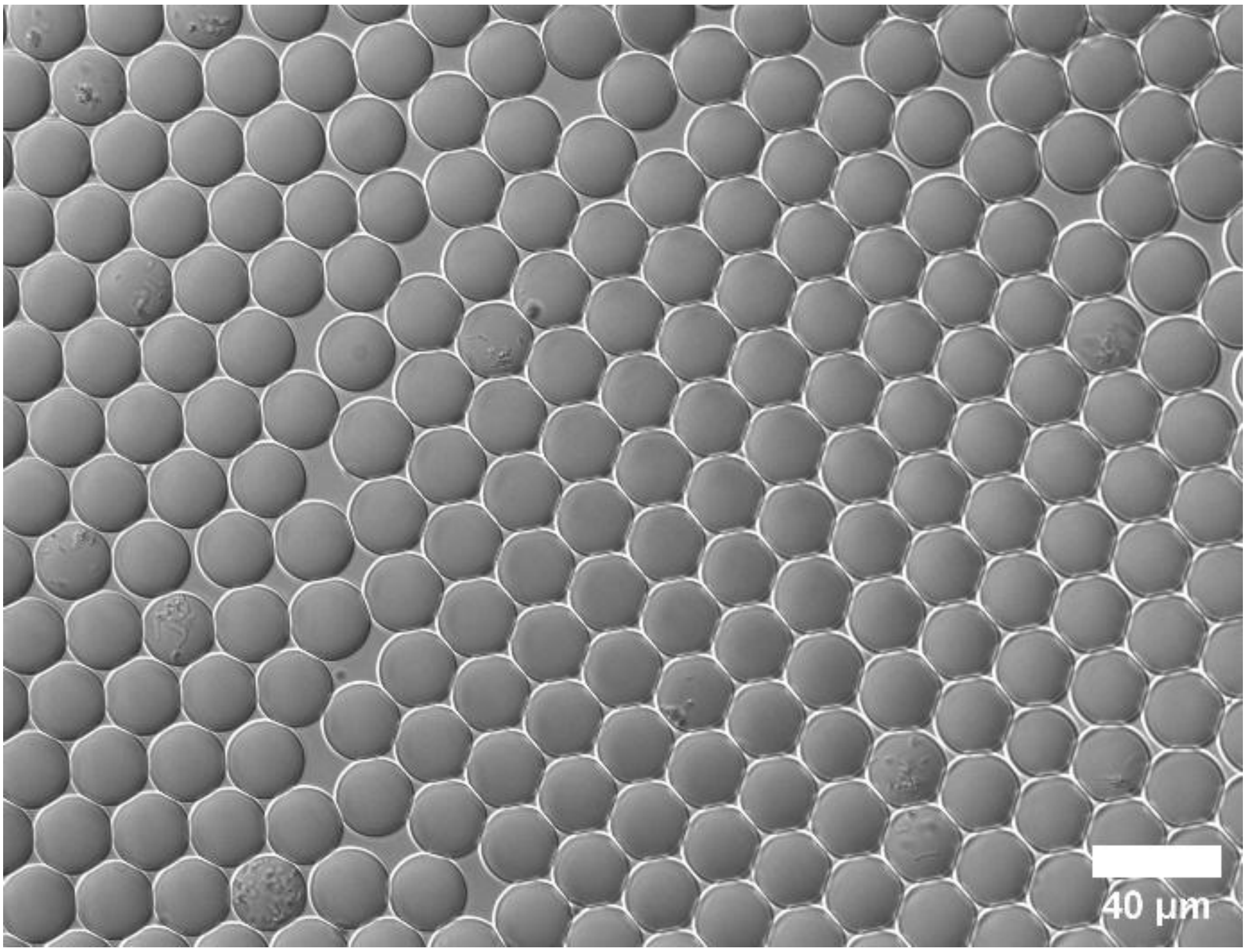

Fig. K Left: Brightfield microscopic image of the 7 pL synthetic community single emulsion population taken with 40x objective (Population 2). The structures inside of the droplets are microcultures of *E. coli* and *C. nitrativorans*.

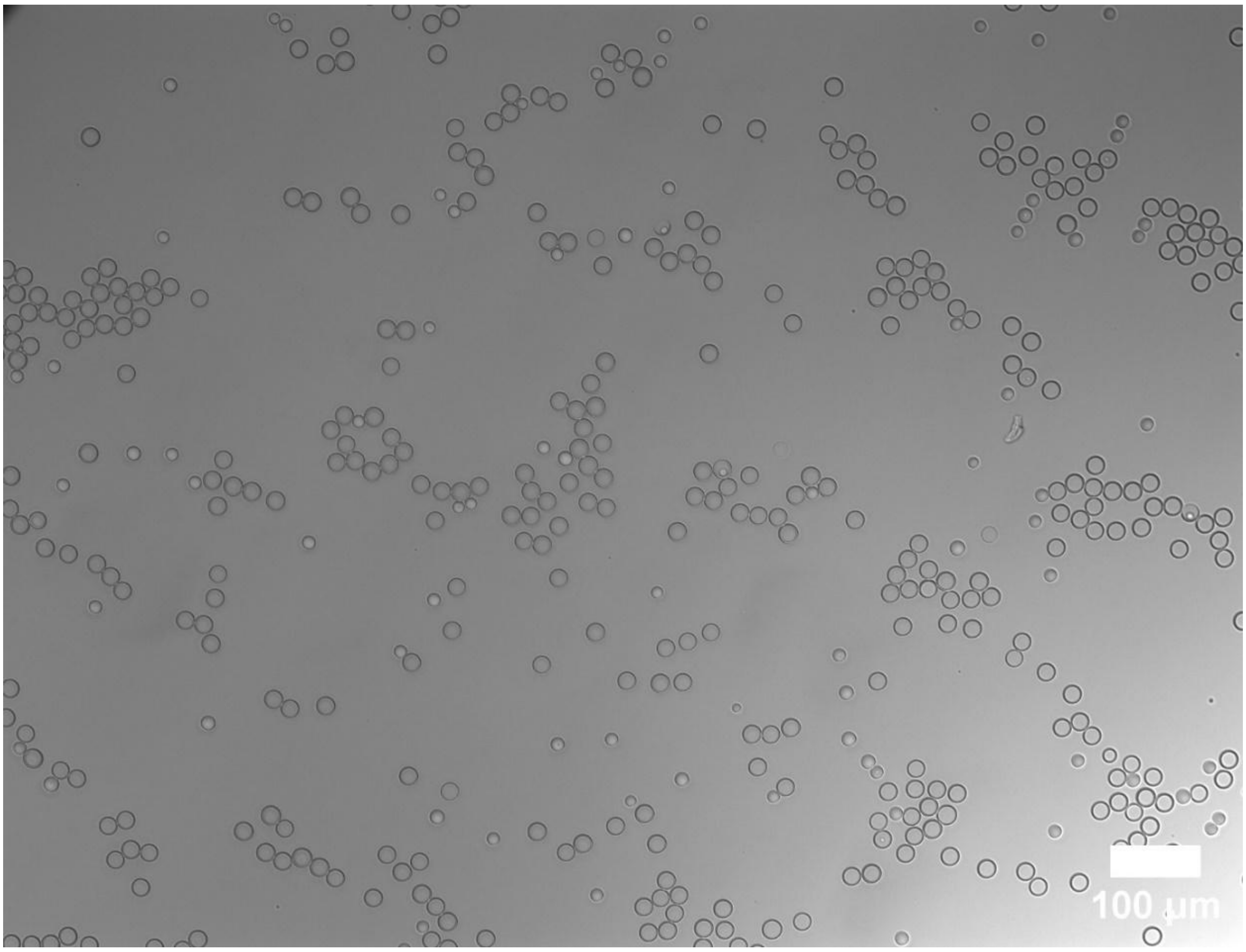

Fig. L Brightfield microscopic image of on-chip produced double emulsions from a 4 pL single emulsion population. The image was captured using a 10x objective lens, it showcases the structure and uniformity of the double emulsions generated on a chip-based microfluidic device.

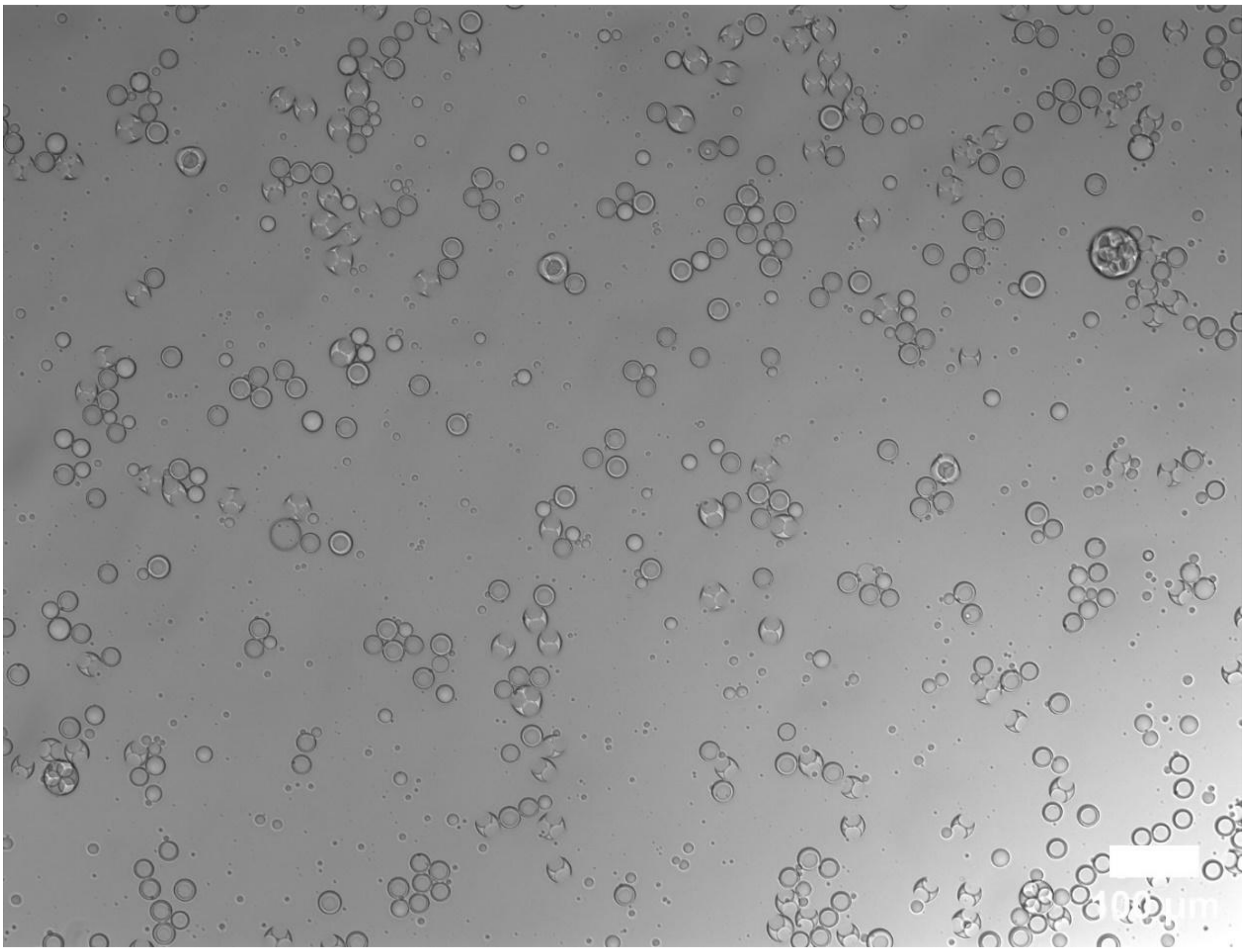

Fig. M Brightfield microscopic image of DTE-produced double emulsions before parameter optimization, starting from a 4 pL single emulsion population. The image was captured using a 10x objective lens, it showcases ordered composition of the vortexed population in single-core, double-core, … nth-core double emulsions. It shows the presence of oil droplets, the monodispersity of single-core double emulsions and the presence of larger artifacts consisting of multiple cores.

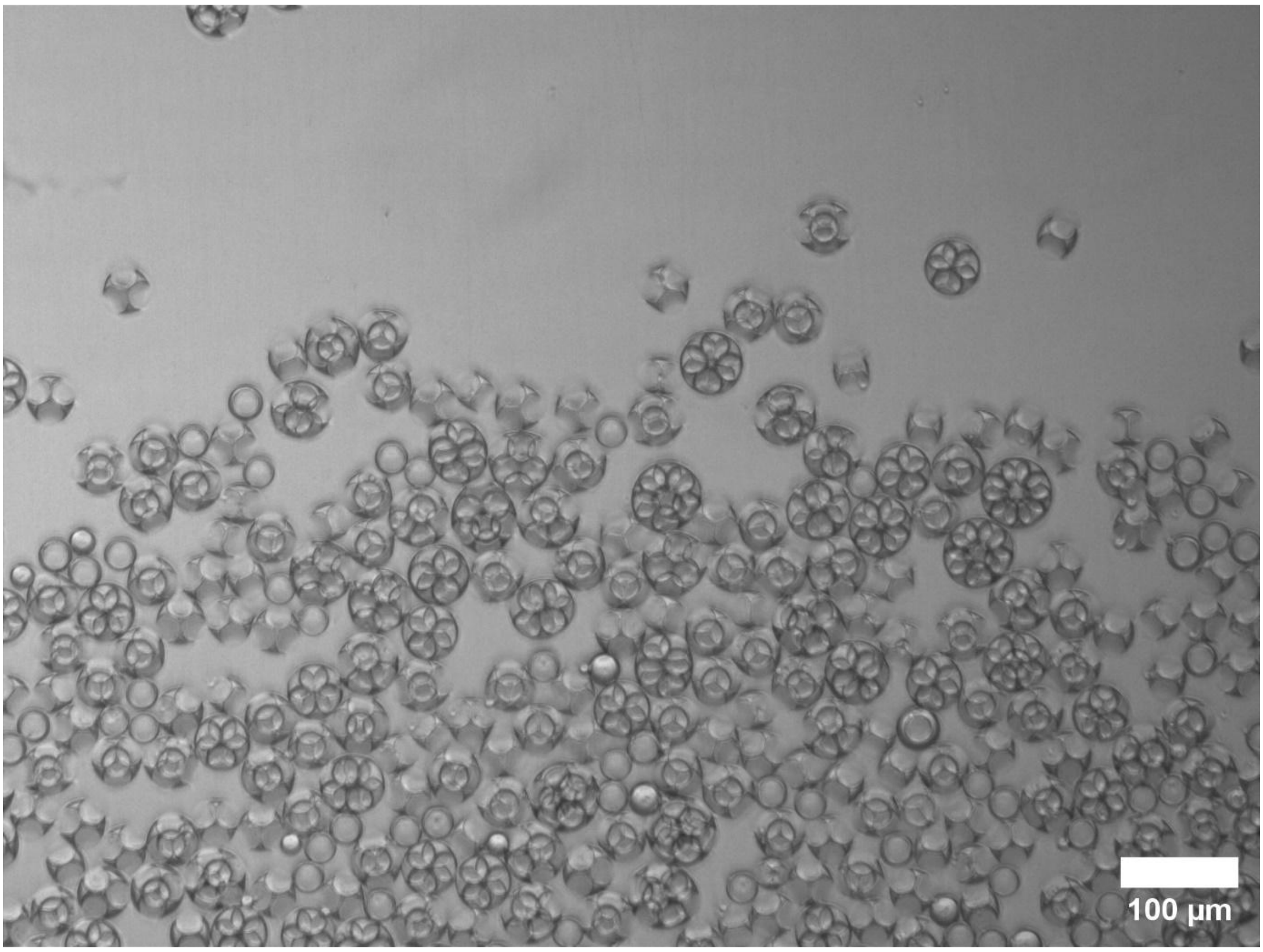

Fig. N Brightfield microscopic image of double emulsions (6 pL single emulsions, emulsified at 300 rpm in 1.5 mL), captured in a 96-well plate. The image showcases a multicore regime. The high-contrast (lighter) objects are oil droplets. The visual appearance is distinct from previous microscopy pictures (Fig. 2 – S 8), due to the use of a different recipient (96-well plate) and a different microscope setup, 10x objective (Zeiss Primo Vert inverted microscope).

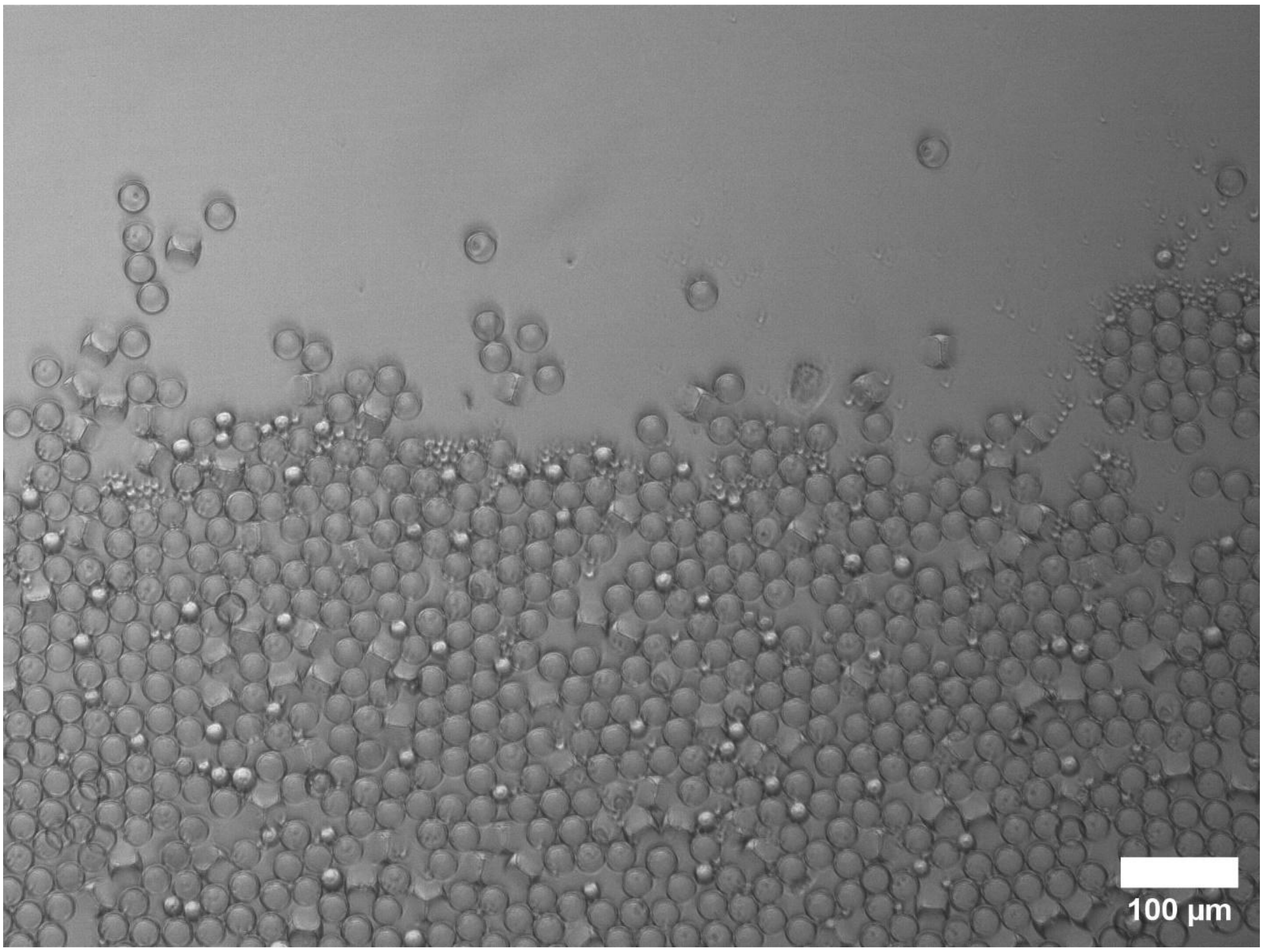

Fig. O. Brightfield microscopic image of double emulsions (6 pL single emulsions, emulsified at 2500 rpm in 1.5 mL), captured in a 96-well plate using an inverted microscope, 10x objective. The image showcases a single core/multicore regime, since there are still a substantial amount of MCDE noted. It also demonstrates the monodispersity of single-core double emulsions made through vortexing. The high-contrast (lighter) objects are oil droplets.

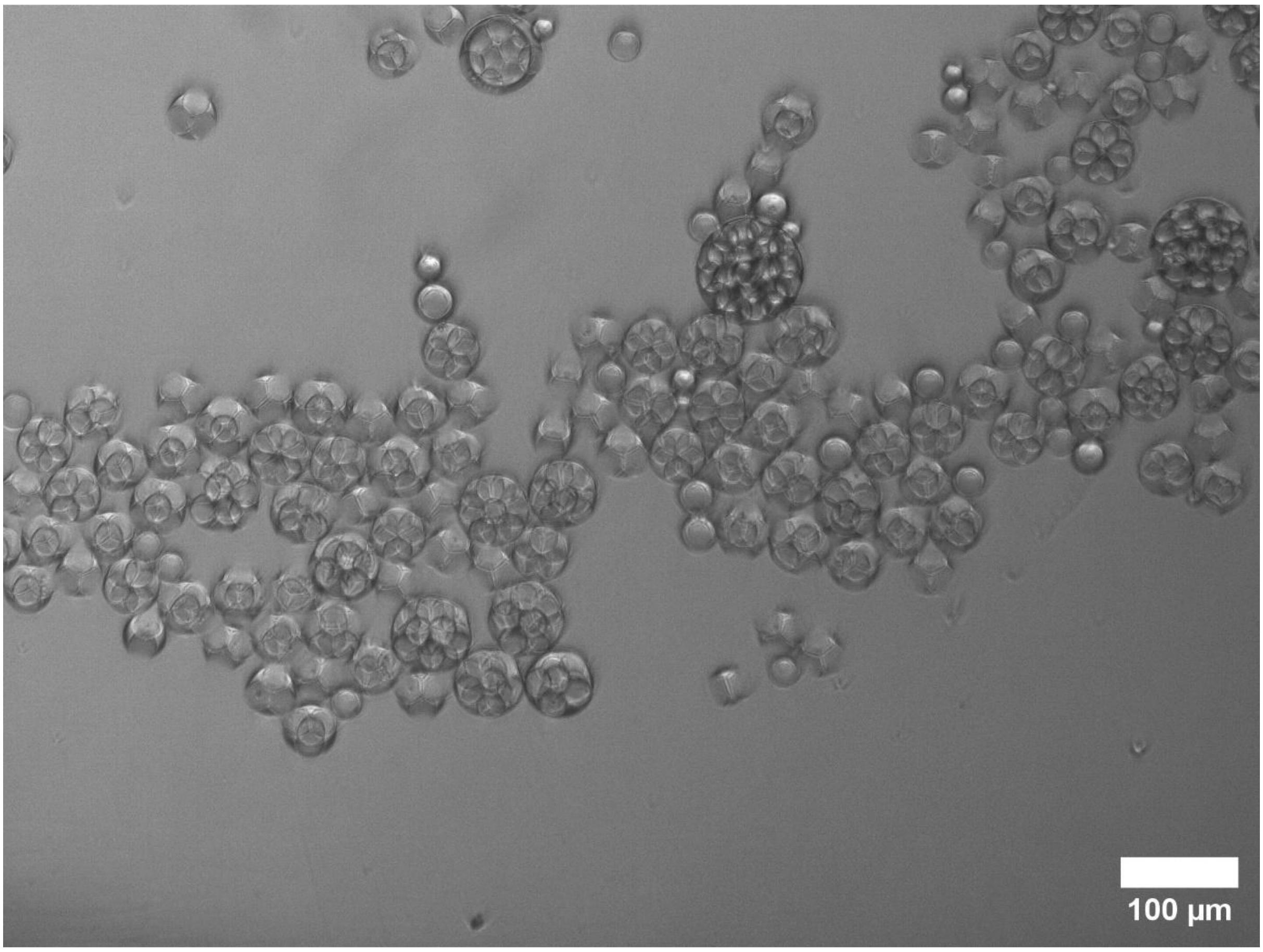

Fig. P Brightfield microscopic image of double emulsions (6 pL single emulsions emulsified at 300 rpm in 4 mL), captured in a 96-well plate using an inverted microscope, 10x objective. The image showcases a multicore regime. The high-contrast (lighter) objects are oil droplets. Very large multi-core double emulsions can be seen.

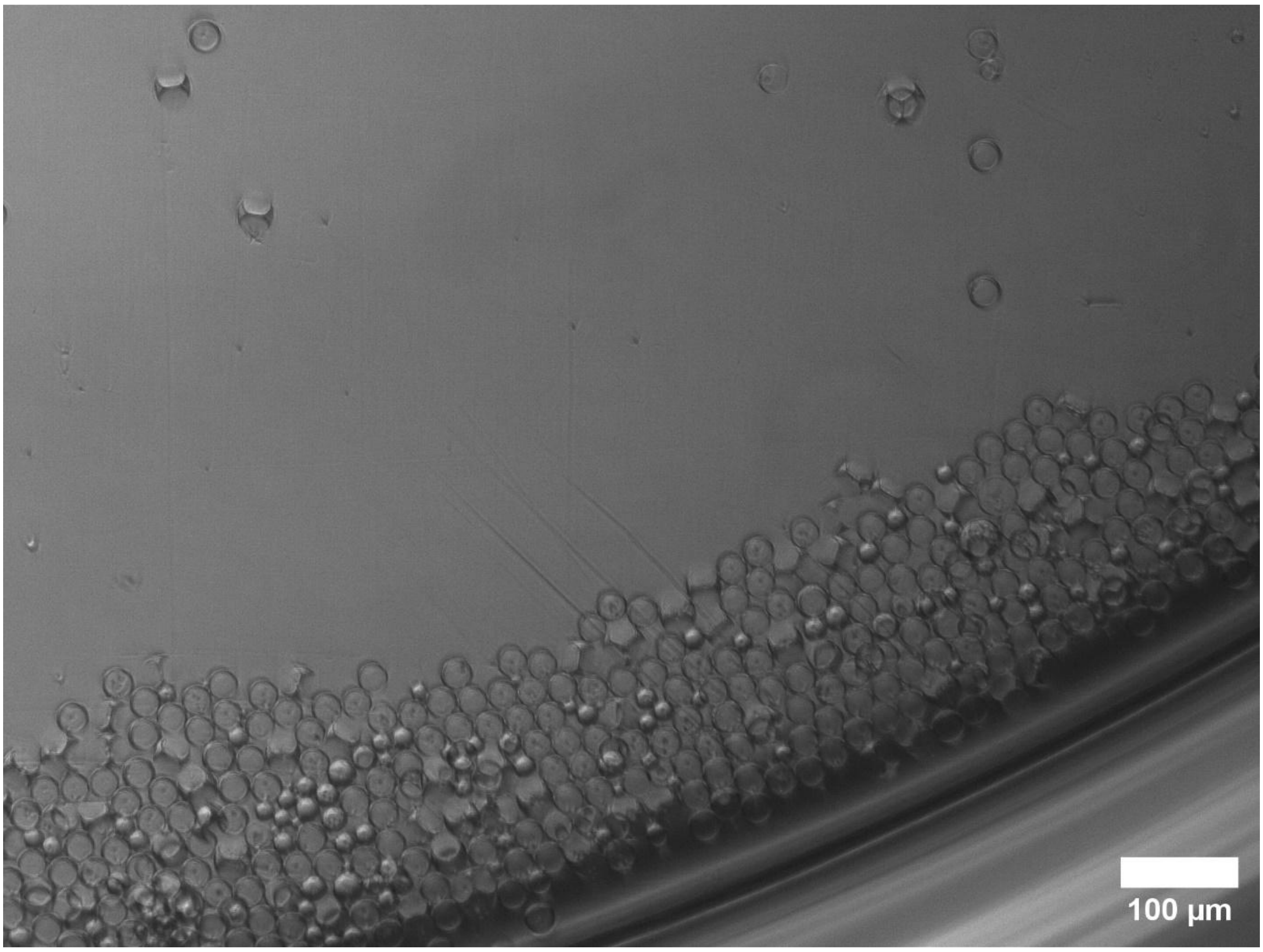

Fig. Q Brightfield microscopic image of double emulsions (6 pL single emulsions, emulsified at 2500 rpm in 4 mL), captured in a 96-well plate using an inverted microscope, 10x objective. The image showcases a SCDE/multicore regime, since there are still a substantial amount of MCDE noted. It also demonstrates the monodispersity of single-core double emulsions made through vortexing. The high-contrast (lighter) objects are oil droplets. Edge of 96-well plate is visible in the bottom right.

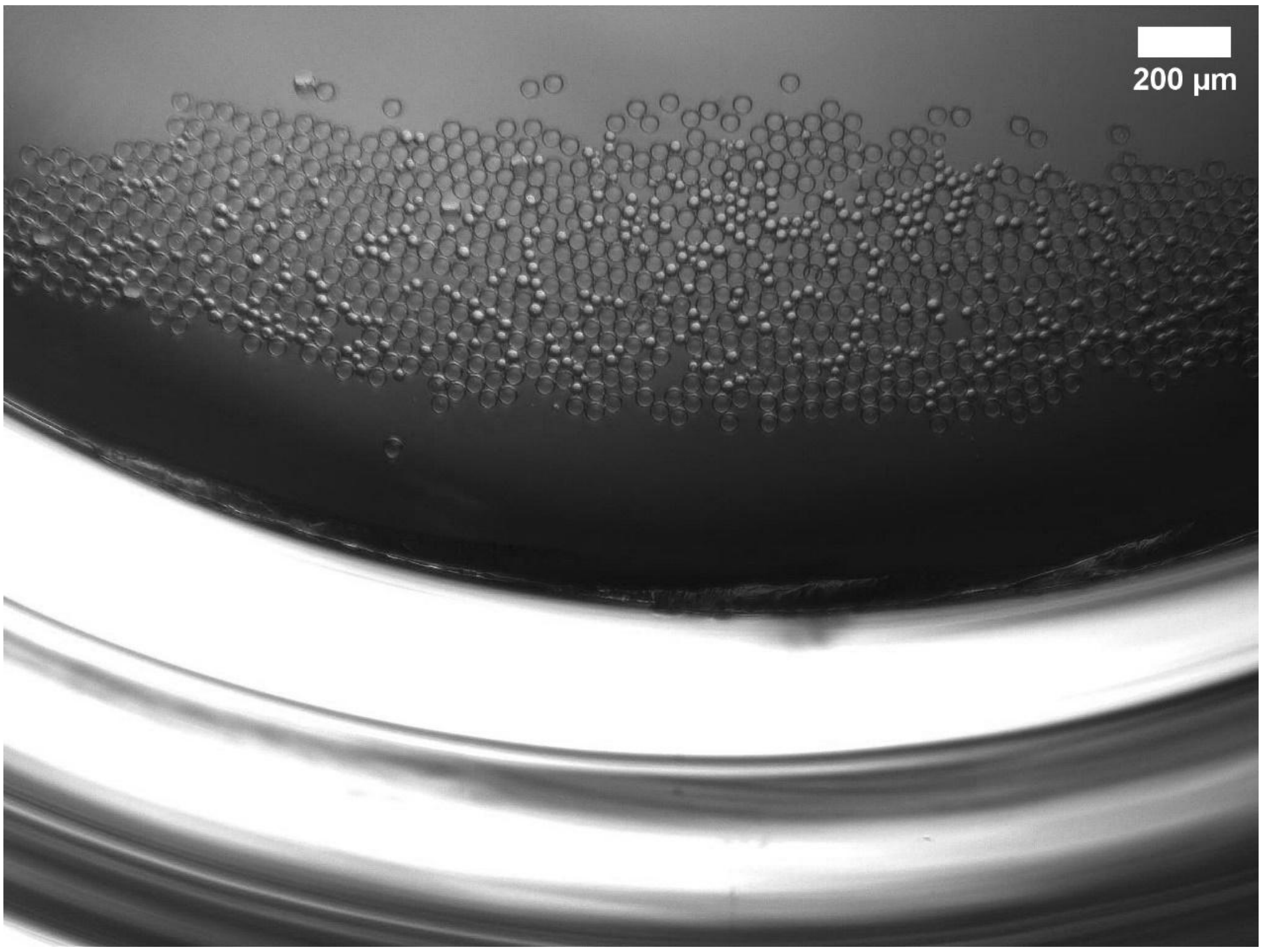

Fig. R Brightfield microscopic image of double emulsions (24 pL single emulsions, emulsified at 300 rpm in 1.5 mL), captured in a 96-well plate using an inverted microscope, 4x objective. The image showcases a single core regime. It also demonstrates the monodispersity of single-core double emulsions made through vortexing. The high-contrast (lighter) objects are oil droplets. The edge of 96-well plate is visible on the bottom of the image.

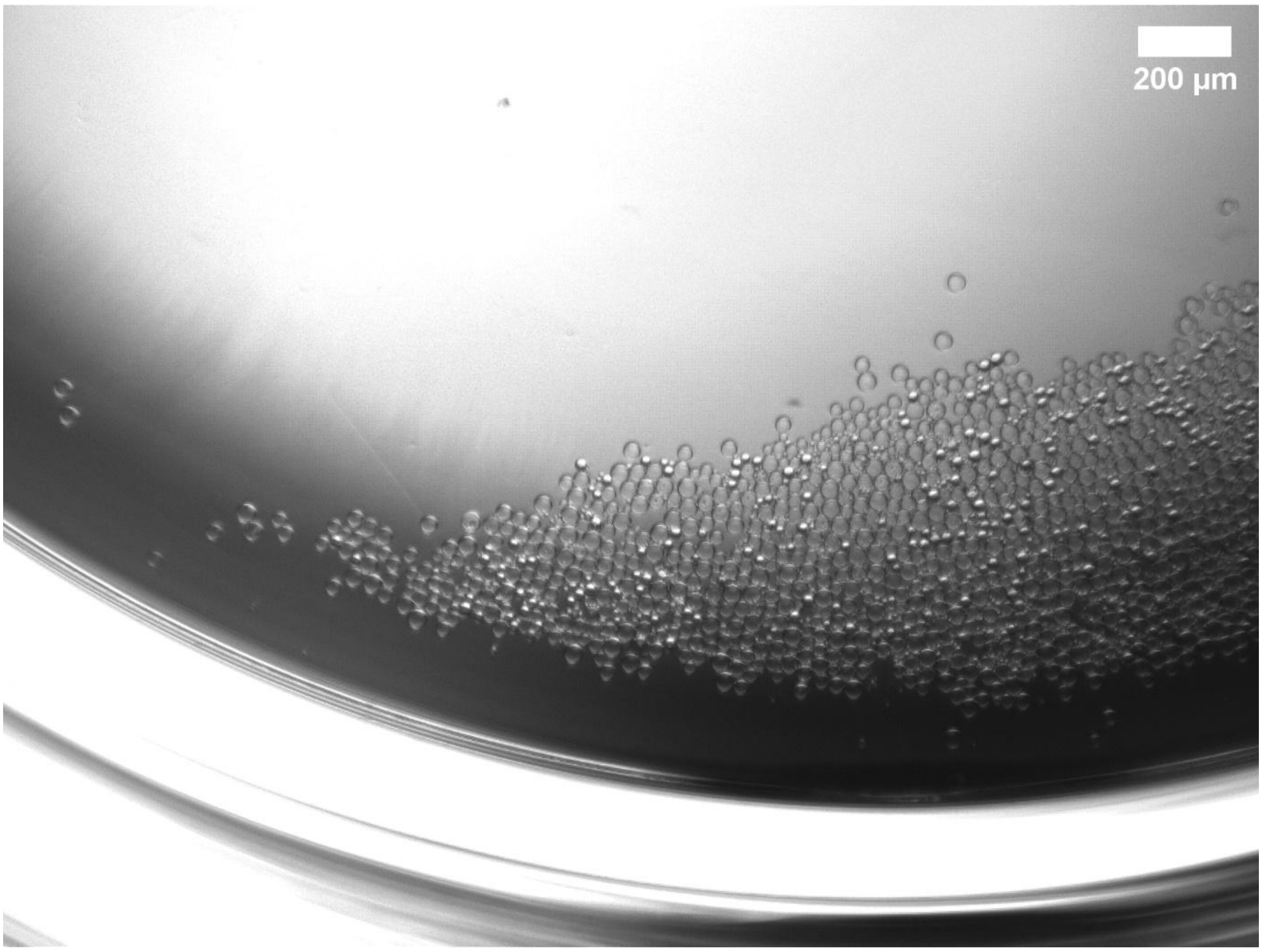

Fig. S Brightfield microscopic image of double emulsions (24 pL single emulsions, emulsified at 2500 rpm in 1.5 mL), captured in a 96-well plate using an inverted microscope, 4x objective. The image showcases a broken core regime, since the SCDE are of different sizes. The high-contrast (lighter) objects are oil droplets. The edge of 96-well plate is visible on the bottom of the image.

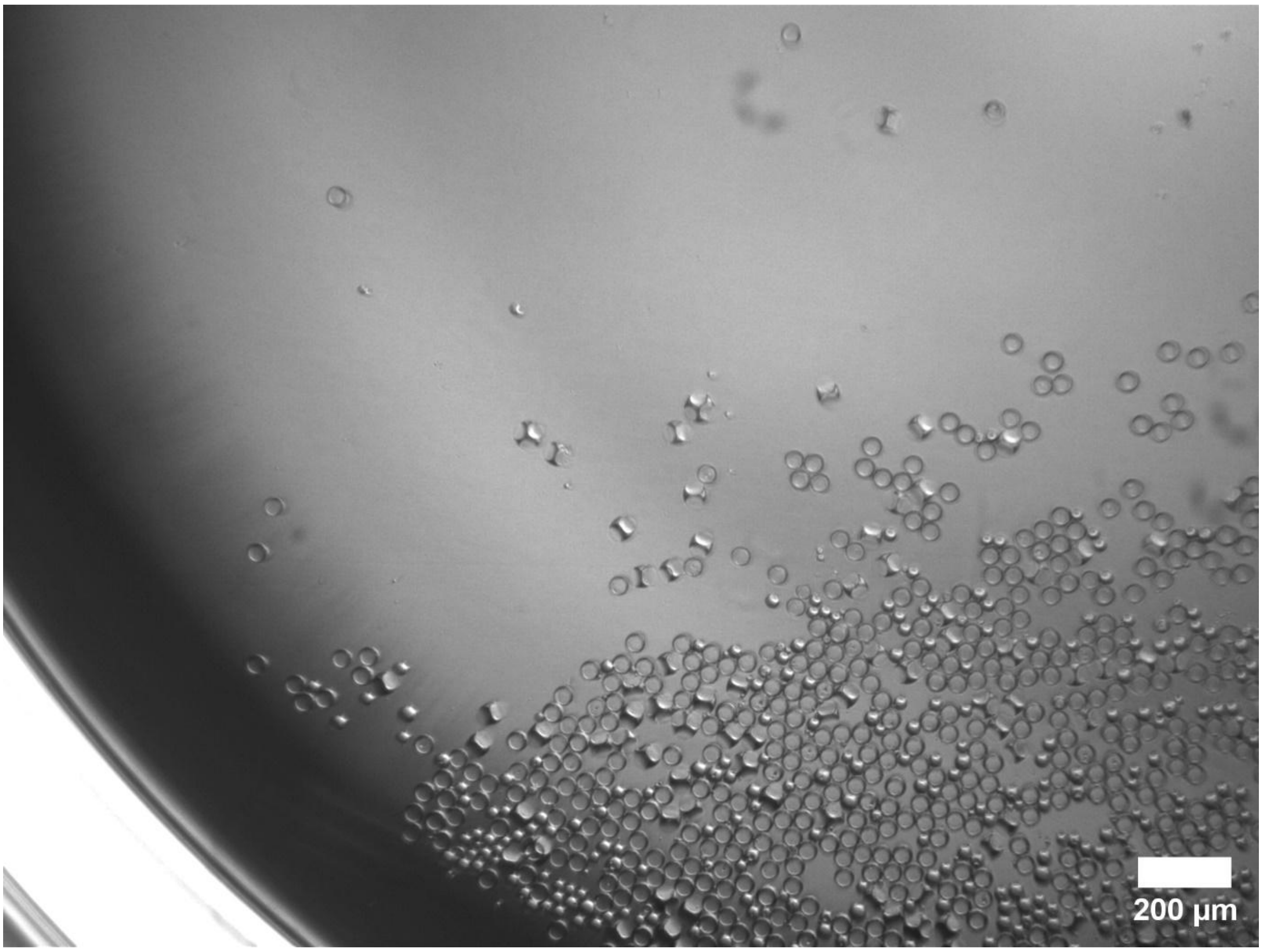

Fig. T Brightfield microscopic image of double emulsions (24 pL single emulsions, emulsified at 300 rpm in 4 mL), captured in a 96-well plate using an inverted microscope, 4x objective. The image showcases a single core/multicore regime, since there are still quite some MCDE noted. It also demonstrates the monodispersity of single-core double emulsions made through vortexing. The high-contrast (lighter) objects are oil droplets. The edge of 96-well plate is visible in the bottom left.

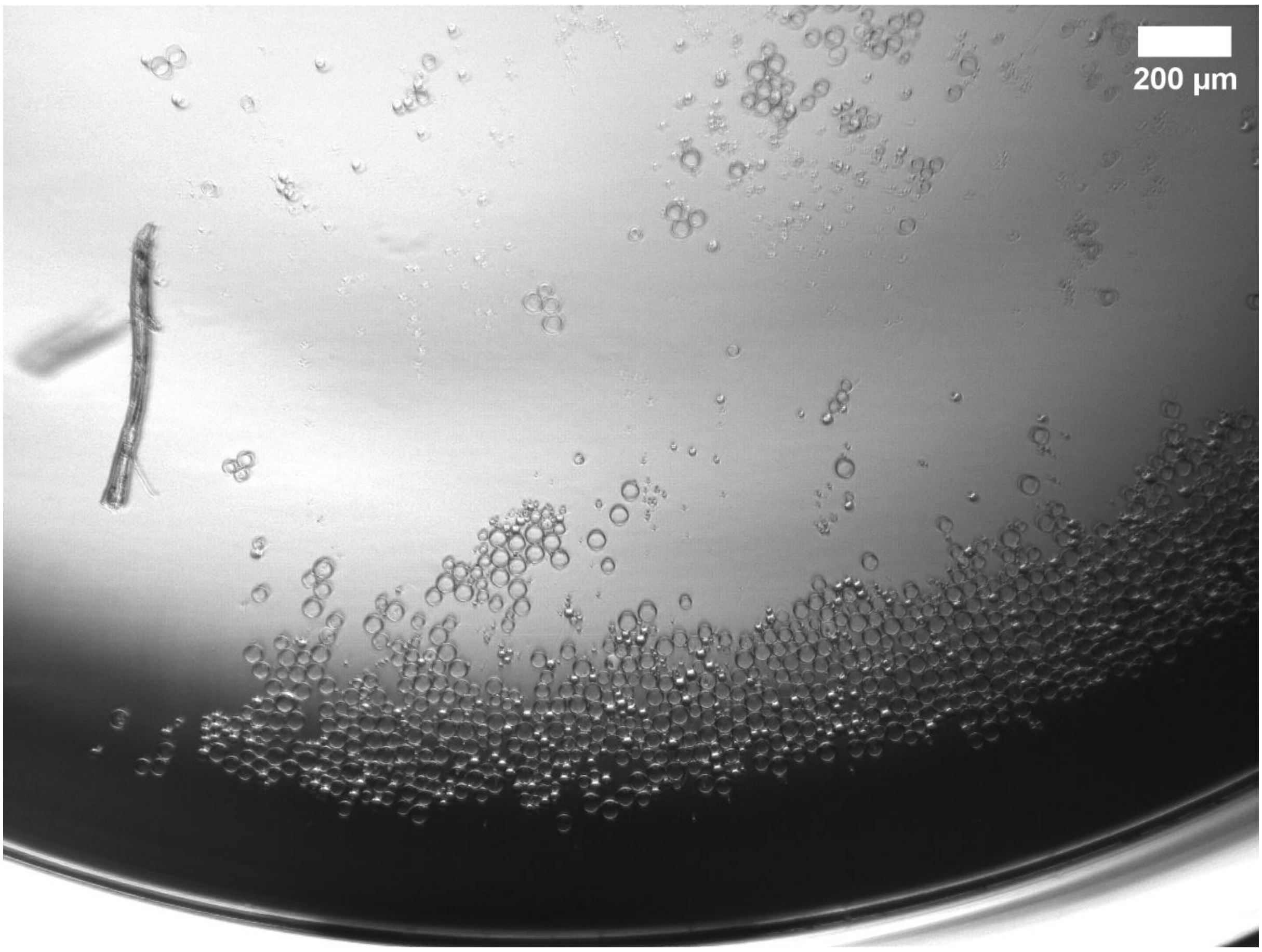

Fig. U Brightfield microscopic image of double emulsions (24 pL single emulsions, emulsified at 2500 rpm in 4 mL), captured in a 96-well plate using an inverted microscope, 4x objective. The image showcases a broken core regime, since the SCDE are broken up into different sizes. The high-contrast (lighter) objects are oil droplets. The edge of 96-well plate is visible in the bottom. A dust particle can be seen on the left.

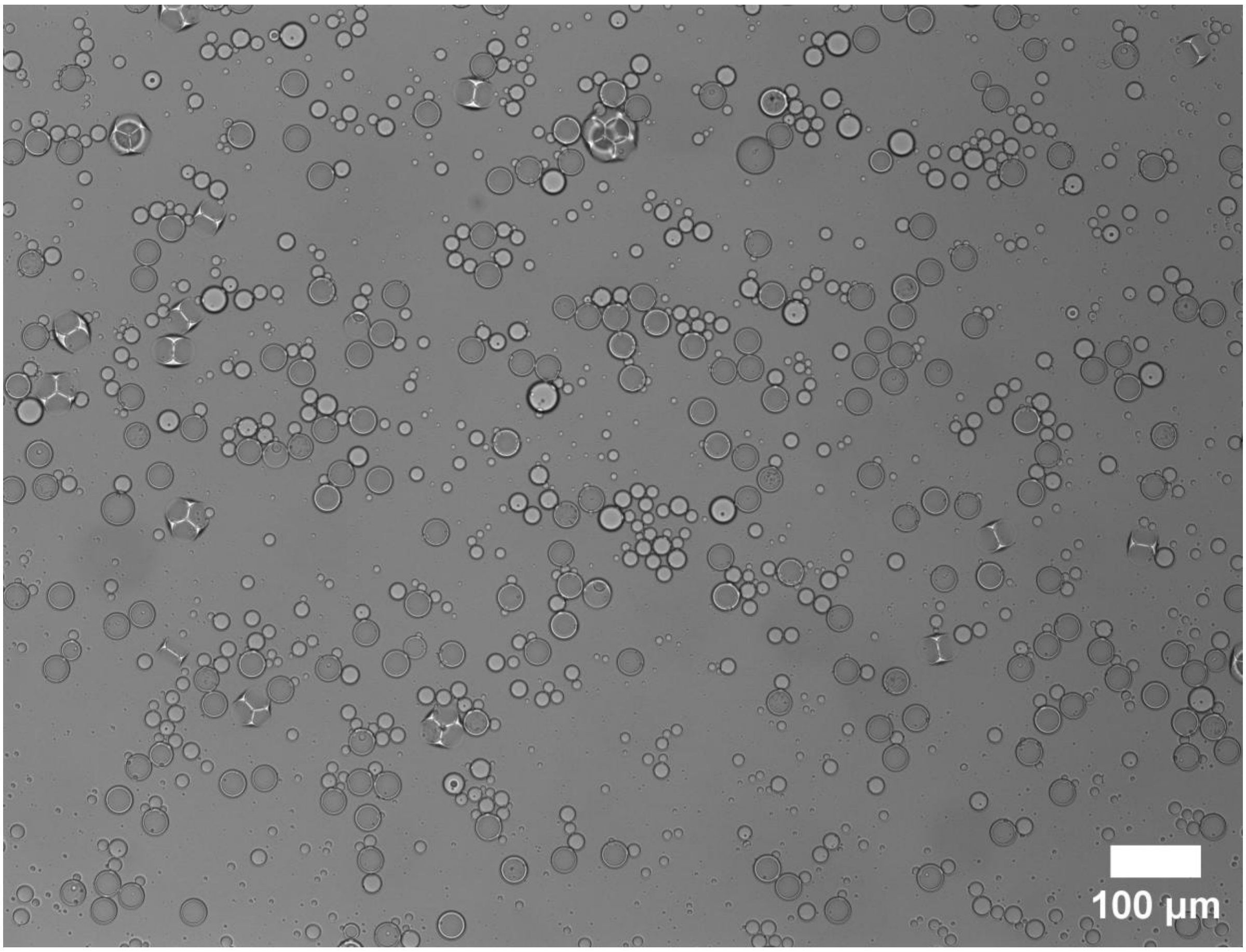

Fig. V 9 pL synthetic community after DTE (Population 1). The dark droplets are double emulsions, the light droplets are oil droplets. An single core/multicore regime can be seen. An increased amount of oil is visible compared to other DTE populations since extra oil was added to allow osmotic swelling.

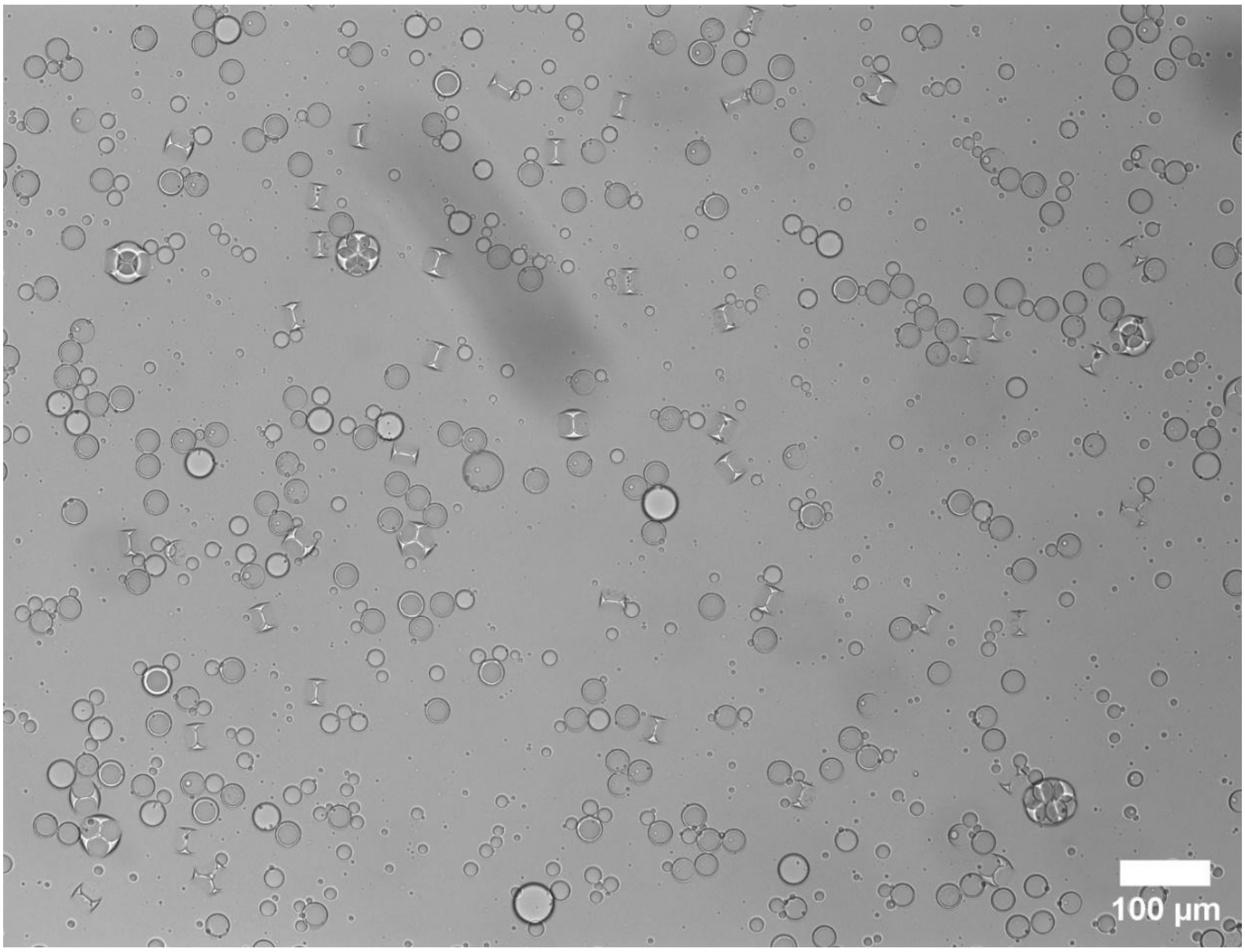

Fig. W. 7 pL synthetic community after DTE (Population 2). The dark droplets are double emulsions, the light droplets are oil droplets. An SCDE/multicore regime can be seen. An increased amount of oil is visible compared to other DTE populations since extra oil was added to allow osmotic swelling.

Results part 2

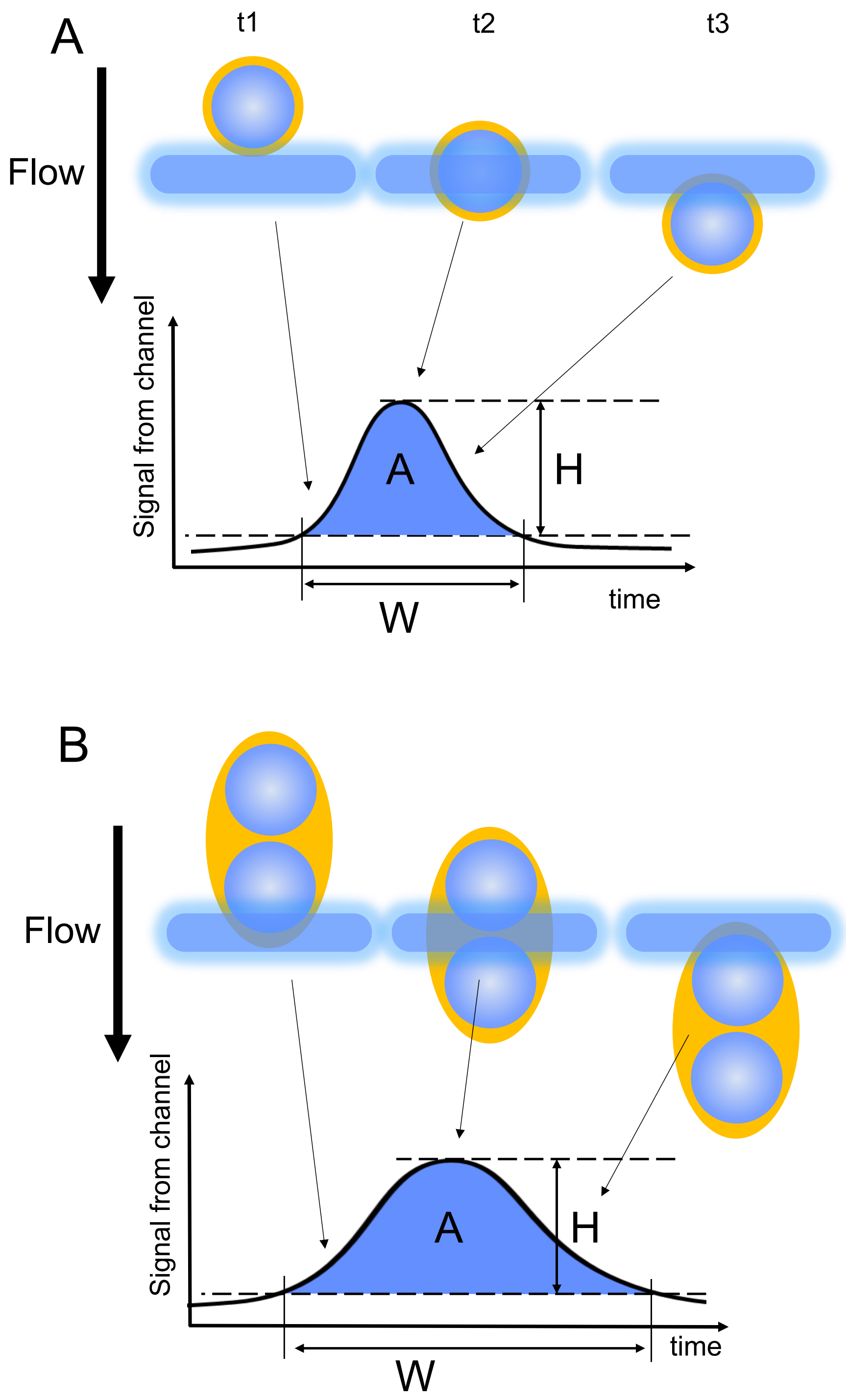

Fig. X A Schematic representation of flow cytometric particle analysis. The blue bar represents the flow cytometer laser. The particle (single core double emulsion in this case) moves with the flow through the laser over time. This generates a pulse in the forward scatter channel (~size of the particle), side scatter channel (~internal structure of particle, larger if more

structured + ~size of the particle) or fluorescent channels of the flow cytometer. From a pulse in a channel, three rudimentary parameters are derived: its height (H), its width (W) and its area (A). Depending on the morphology of the particle and the considered measured parameter, pulse shapes will be different as demonstrated in B, where a longer particle results in an increased travel time, leading to an increased width and area of the pulse, while the height stays the same. In our case, a suitable parameter would measure the increased internal complexity of a multicore double emulsion as an increase in either width (as demonstrated here), area or height, the larger the difference in signal between single core and multicore double emulsions, the more suitable the parameter is (B).

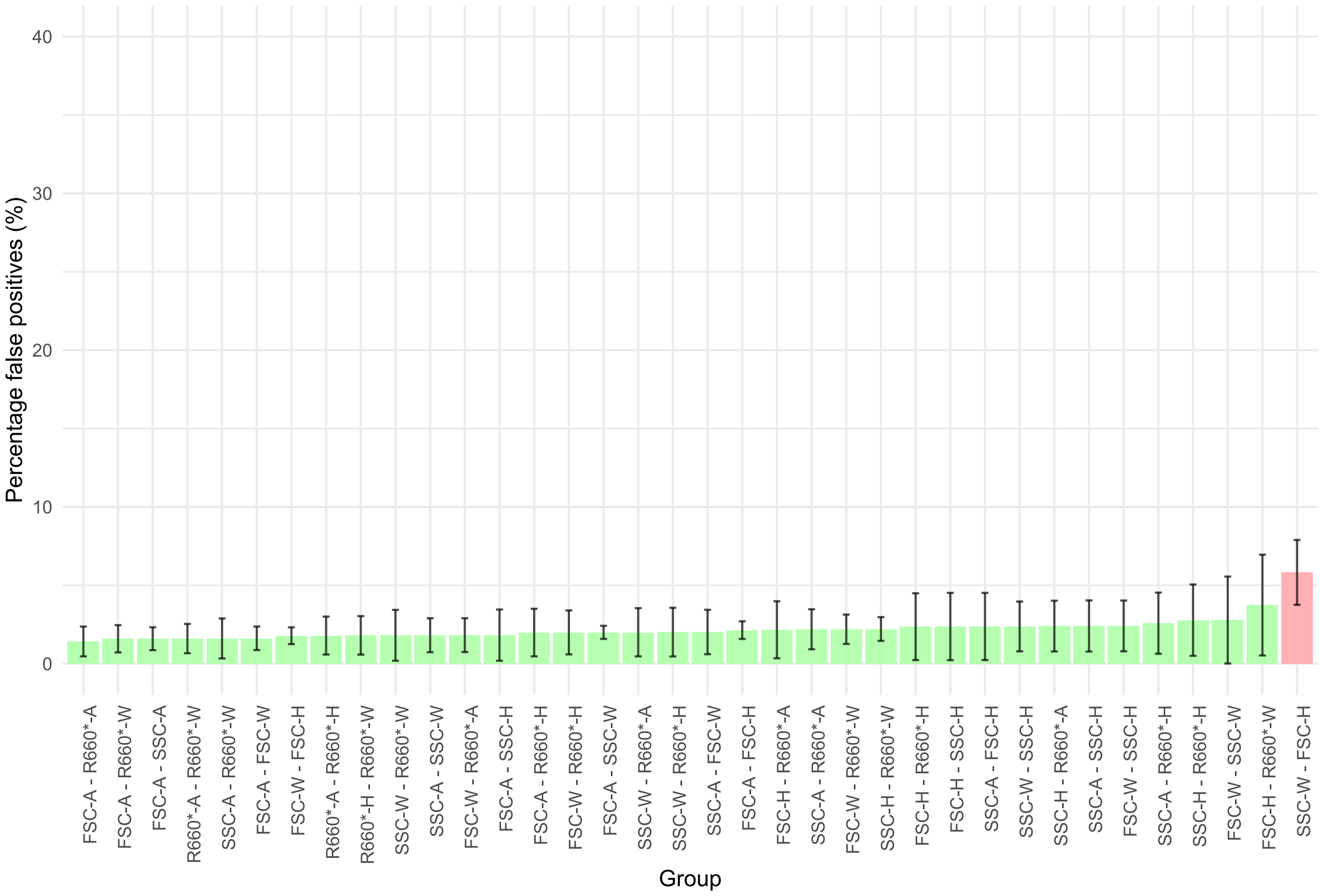

Fig. Y Barplots showing the mean false positive percentage included in the drawn gate during automated gating of each parameter pair in the low multicore scenario. Error flags are standard deviations (n = 3). The R660* channel is a red fluorescence channel, measuring the sCy5 droplet dye we included to look at fluorescent signal coming from the droplet cores, mimicking

assay signal. We set an arbitrary limit at 5 % false positives for this figure, parameter pairs with false positive rates larger than 5 % are coloured red.

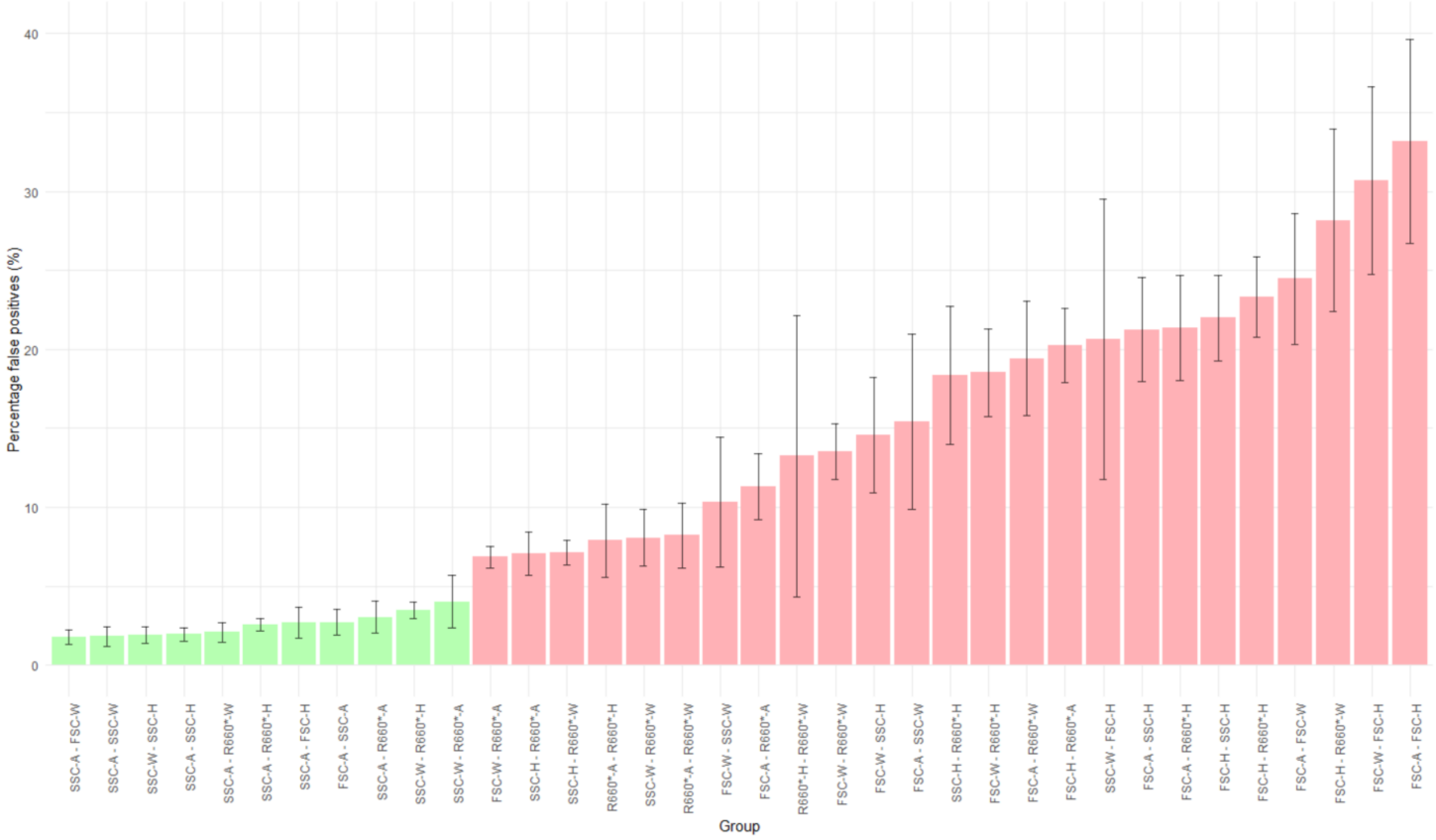

Fig. Z Barplots showing the mean false positive percentage included in the drawn gate during automated gating of each parameter pair in the high multicore scenario. Error flags are standard deviations (n = 3). The R660* channel is a red fluorescence channel, measuring the sCy5 droplet dye we included to look at fluorescent signal coming from the droplet cores, mimicking assay signal. We set an arbitrary limit at 5 % false positives for this figure, parameter pairs with false positive rates larger than 5 % are coloured red.

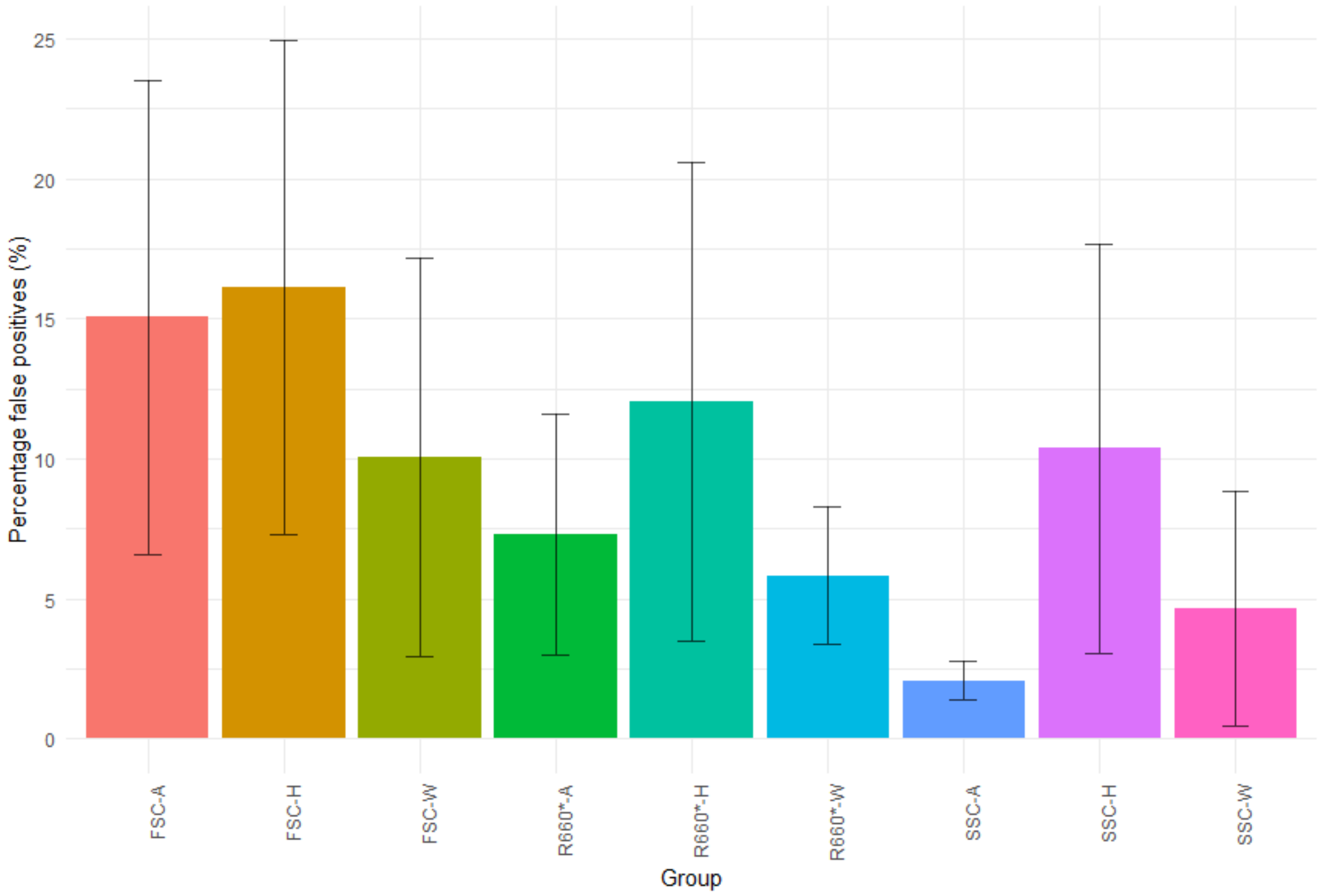

Fig. AA Performance of single parameters averaged out over all parameter pairs.

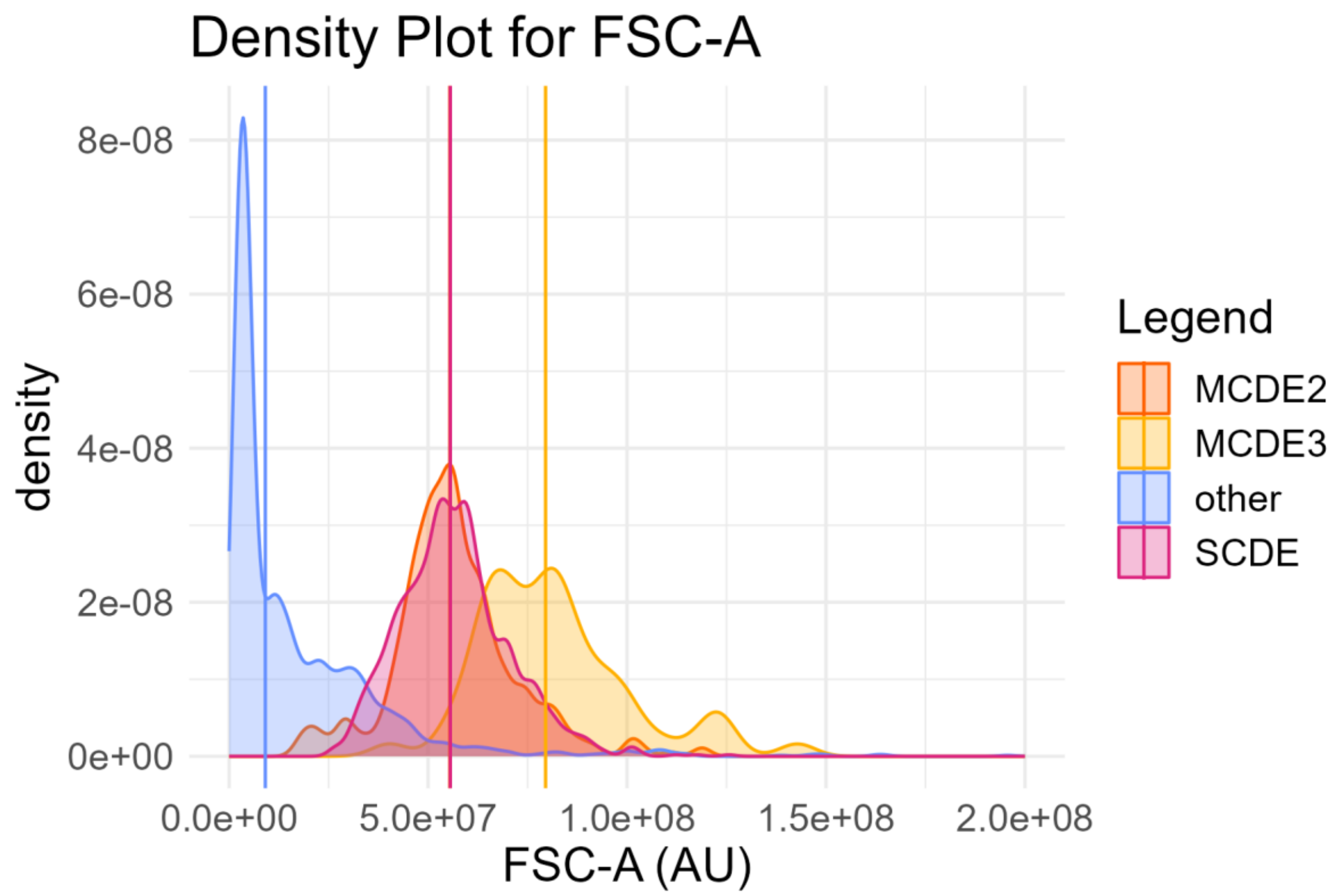

Density Plot for FSC-A
density
8e-08
6e-08
4e-08
2e-08
0e+00
0.0e+00
5.0e+07
1.0e+08
1.5e+08
2.0e+08
FSC-A (AU)
Legend
MCDE2
MCDE3
other
SCDE

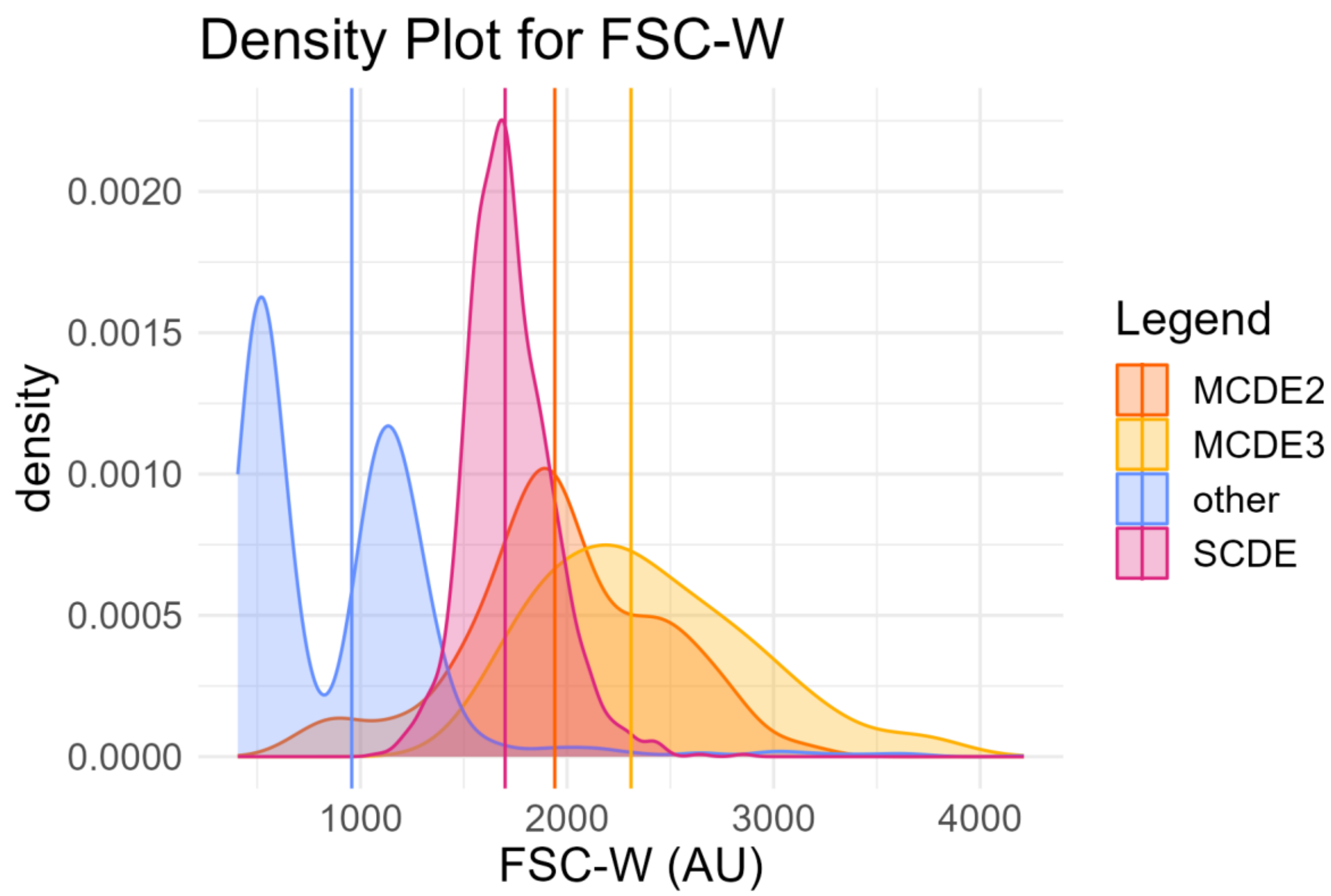

Density Plot for FSC-W
density
0.0020
0.0015
0.0010
0.0005
0.0000
1000
2000
3000
4000
FSC-W (AU)
Legend
MCDE2
MCDE3
other
SCDE

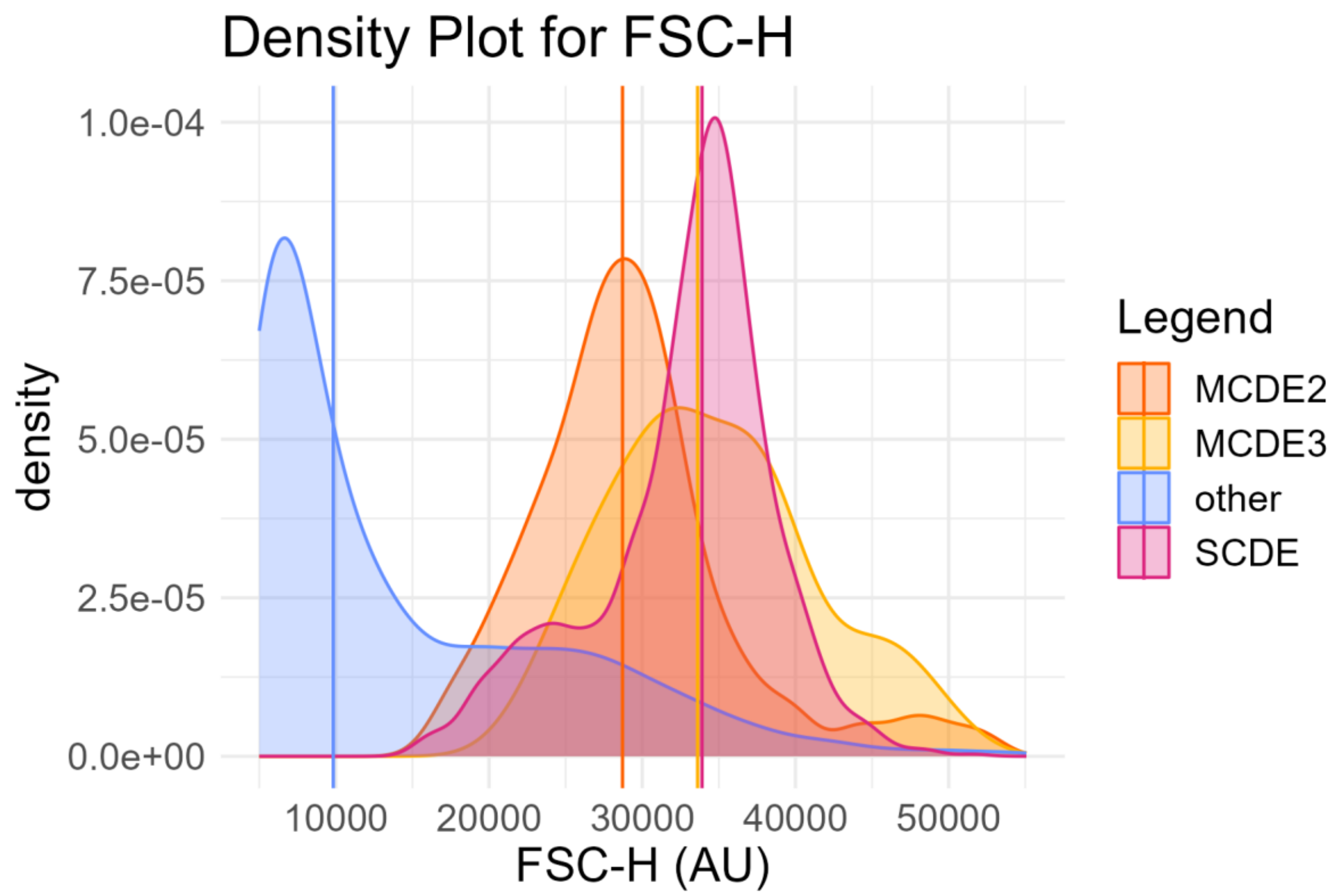

Fig. BB Density plots of forward scatter (FSC) A-, W-, and H-channels of all events in the high multicore scenario. These plots allow assessing qualitative clustering performance for a single parameter. Events are coloured by morphology. Magenta: single core double emulsions (SCDE), orange: double core double emulsions (MCDE2) and yellow: triple core double emulsions (MCDE3). Lines are drawn for the population medians in the corresponding colours.

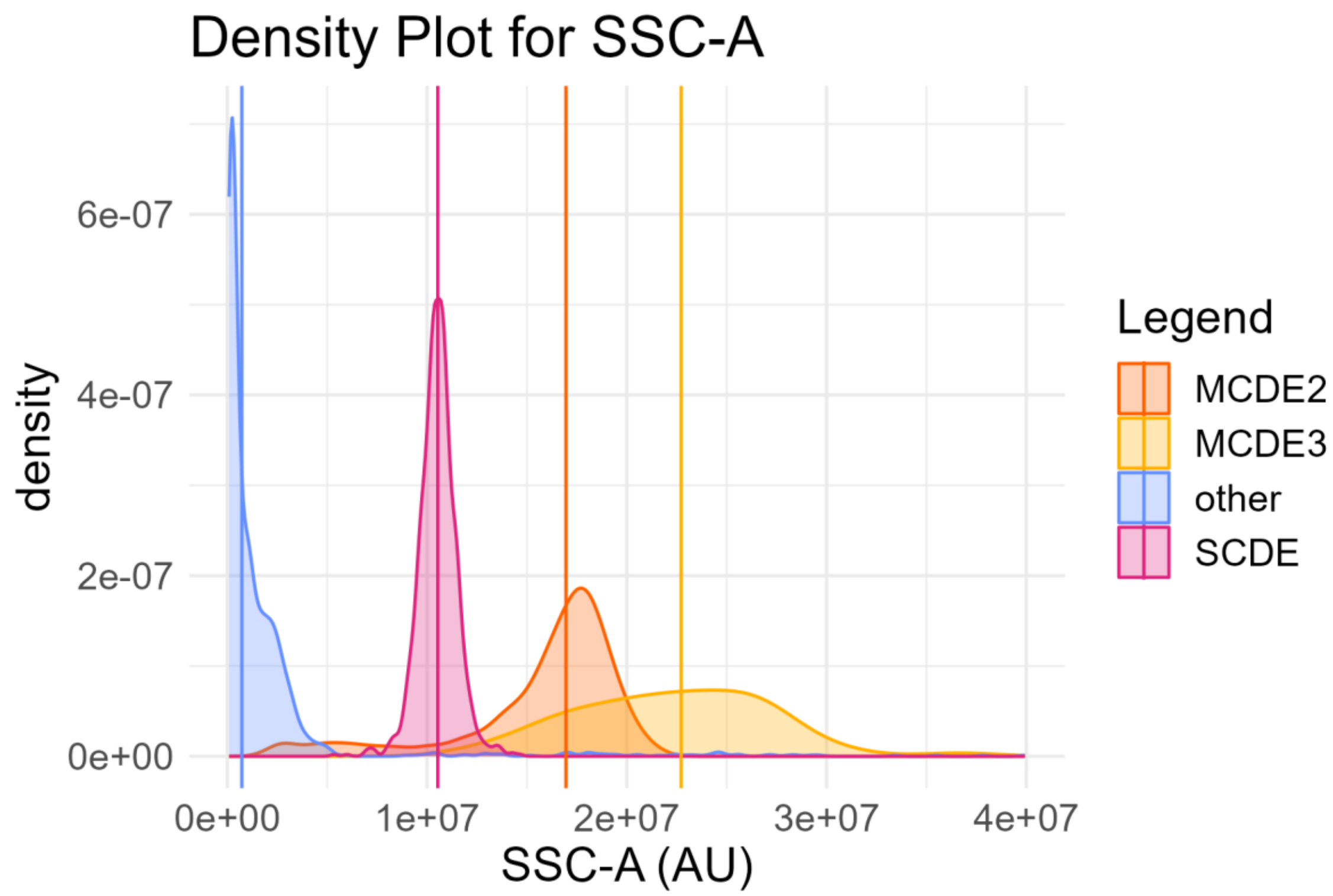
Density Plot for SSC-A
density
6e-07
4e-07
2e-07
0e+00
0e+00
1e+07
2e+07
3e+07
4e+07
SSC-A (AU)
Legend
MCDE2
MCDE3
other
SCDE

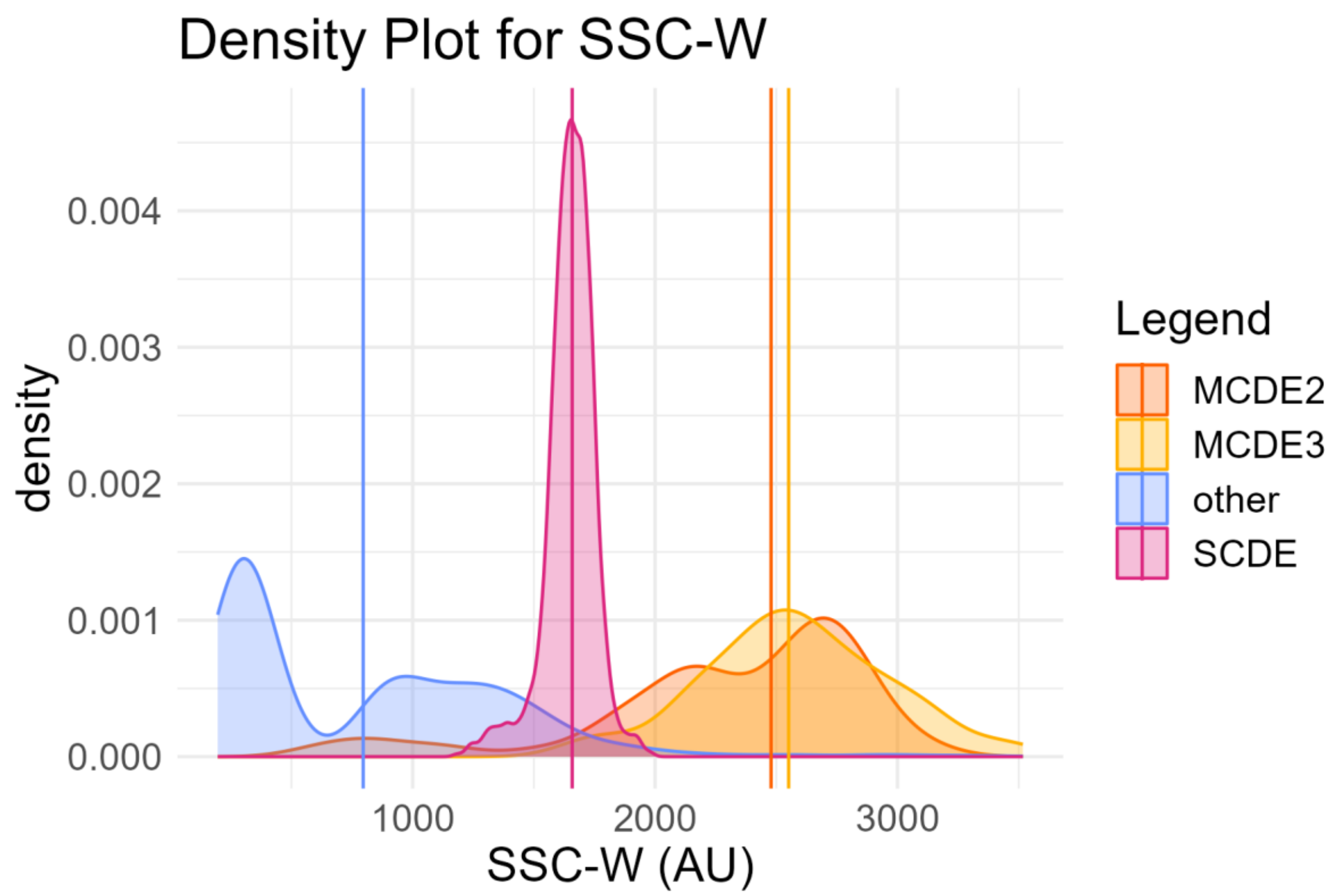
Density Plot for SSC-W
density
0.004
0.003
0.002
0.001
0.000
1000
2000
3000
SSC-W (AU)
Legend
MCDE2
MCDE3
other
SCDE

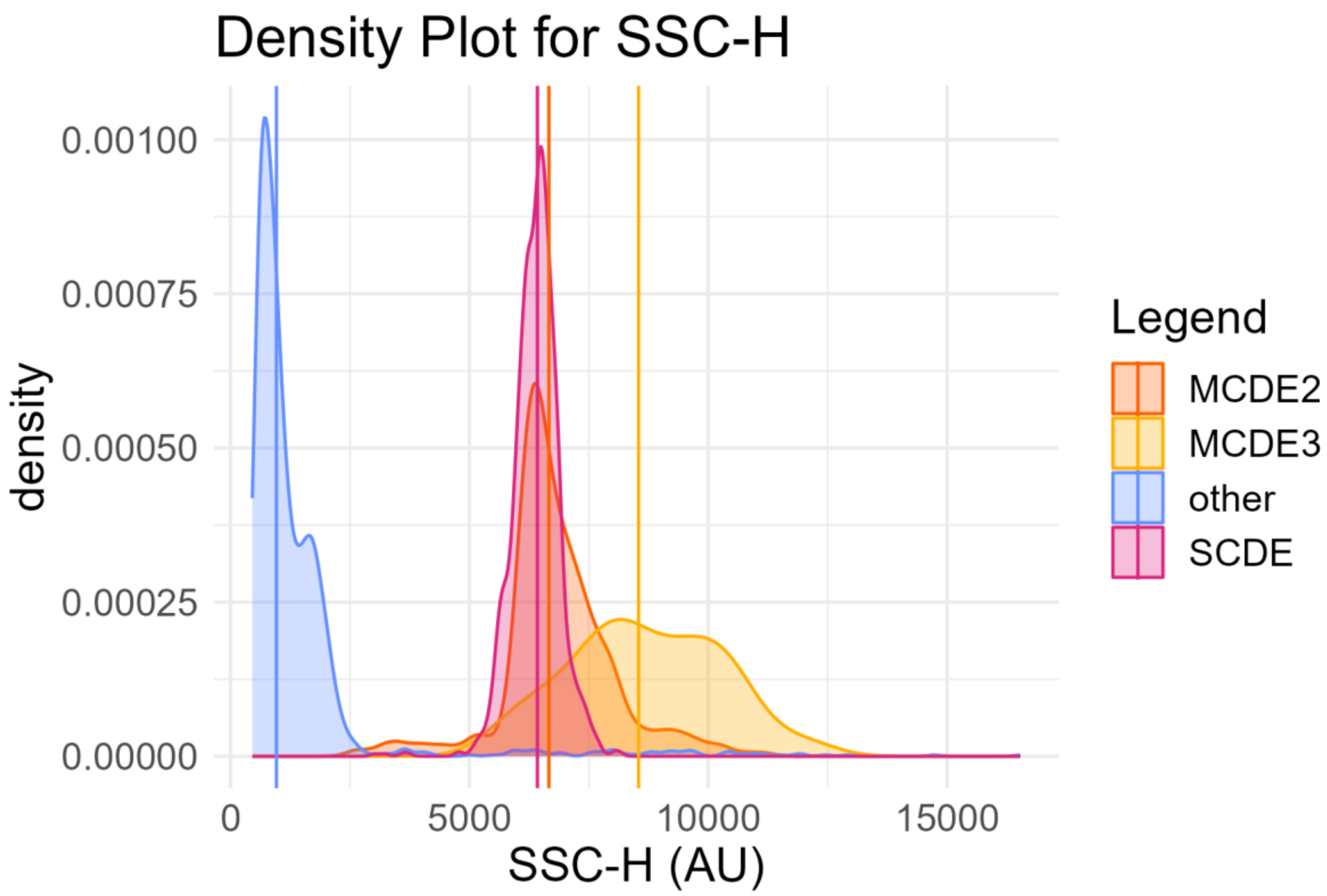

Fig. CC Density plots of side scatter (SSC) A-, W-, and H-channels of all events in the high multicore scenario. These plots allow assessing qualitative clustering performance for a single parameter. Events are coloured by morphology. Magenta: single core double emulsions (SCDE), orange: double core double emulsions (MCDE2) and yellow: triple core double emulsions (MCDE3). Lines are drawn for the population medians in the corresponding colours.

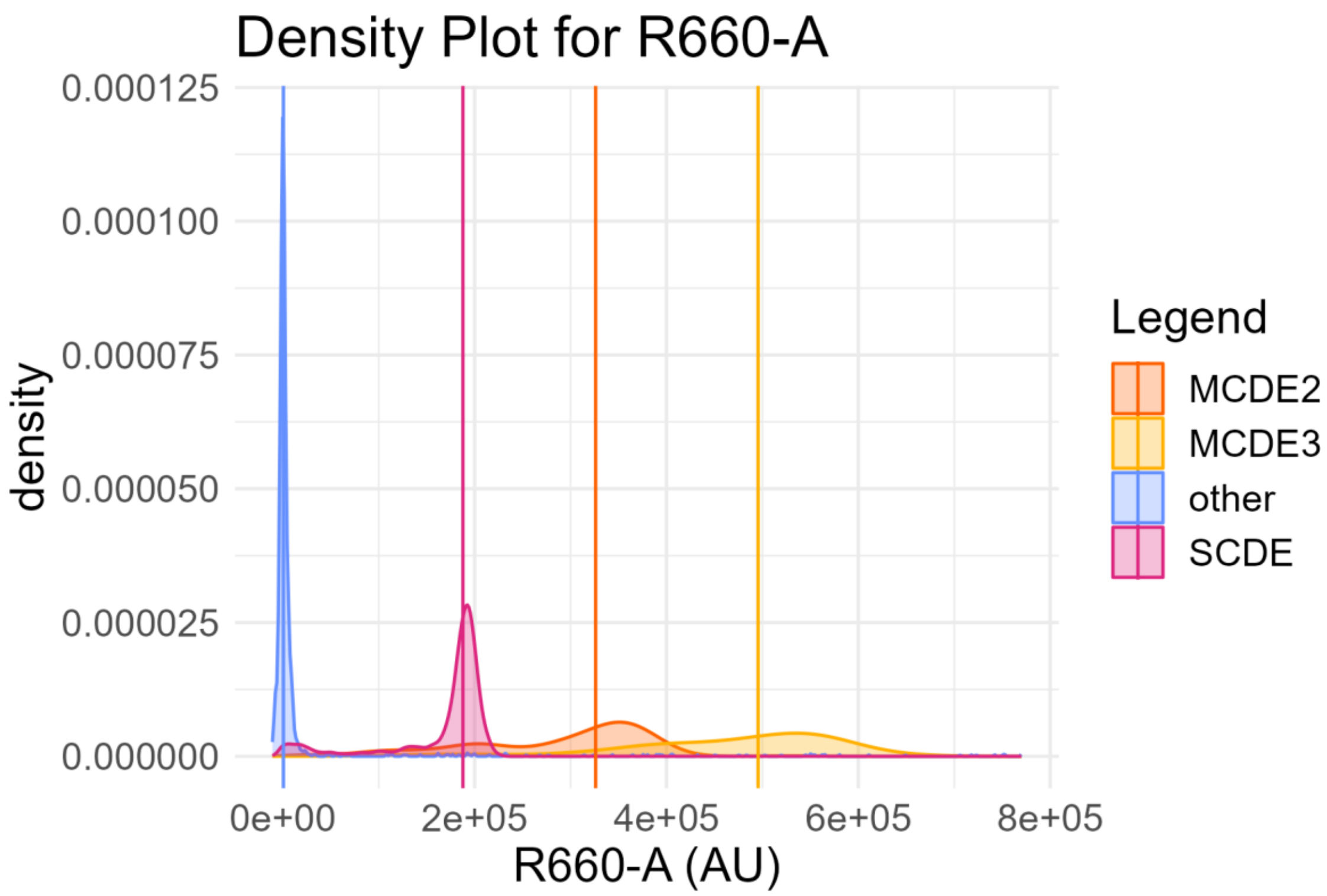

Density Plot for R660-A
0.000125
0.000100
0.000075
0.000050
0.000025
0.000000
density
0e+00
2e+05
4e+05
6e+05
8e+05
R660-A (AU)
Legend
MCDE2
MCDE3
other
SCDE

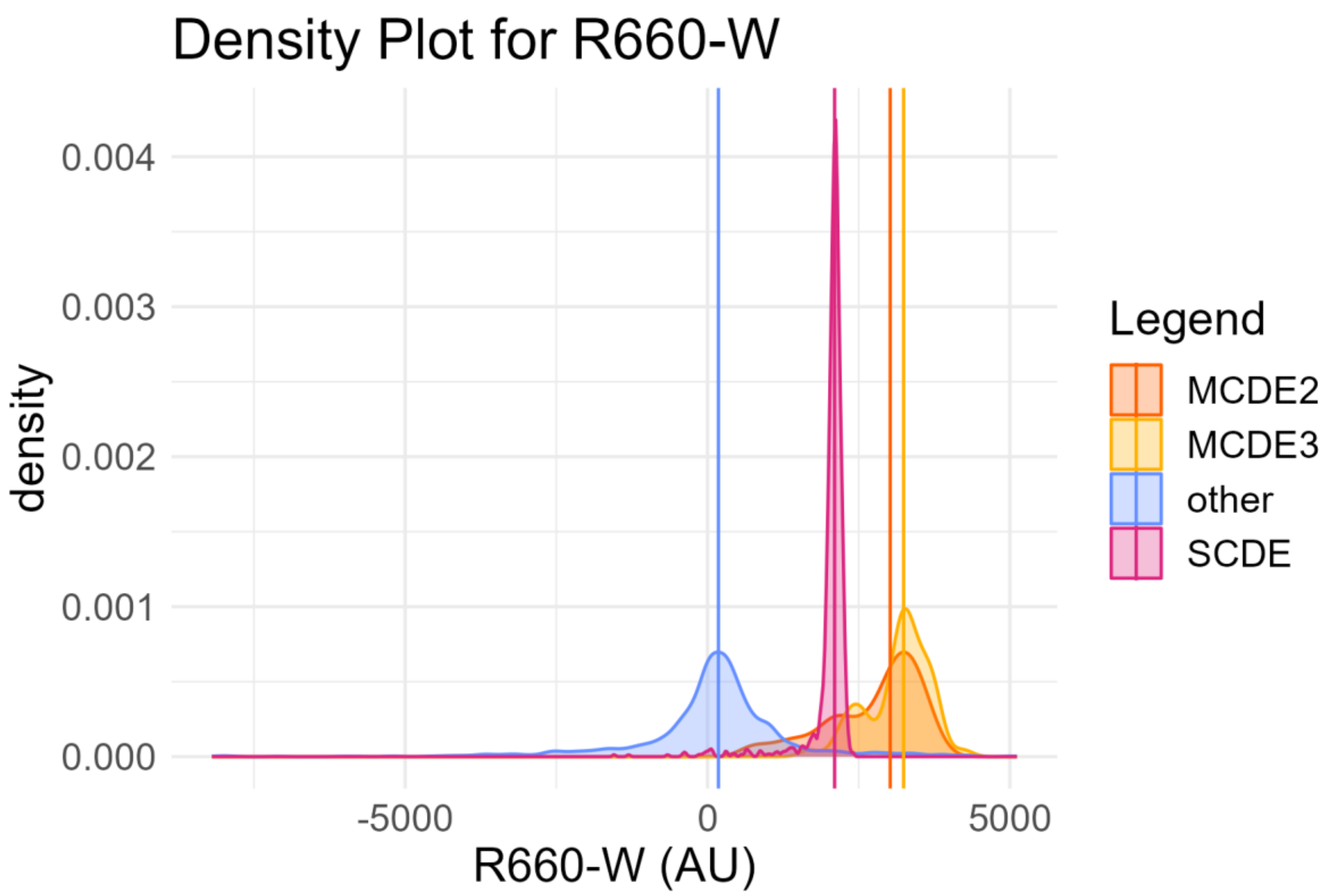

Density Plot for R660-W
0.004
0.003
0.002
0.001
0.000
density
-5000
0
5000
R660-W (AU)
Legend
MCDE2
MCDE3
other
SCDE

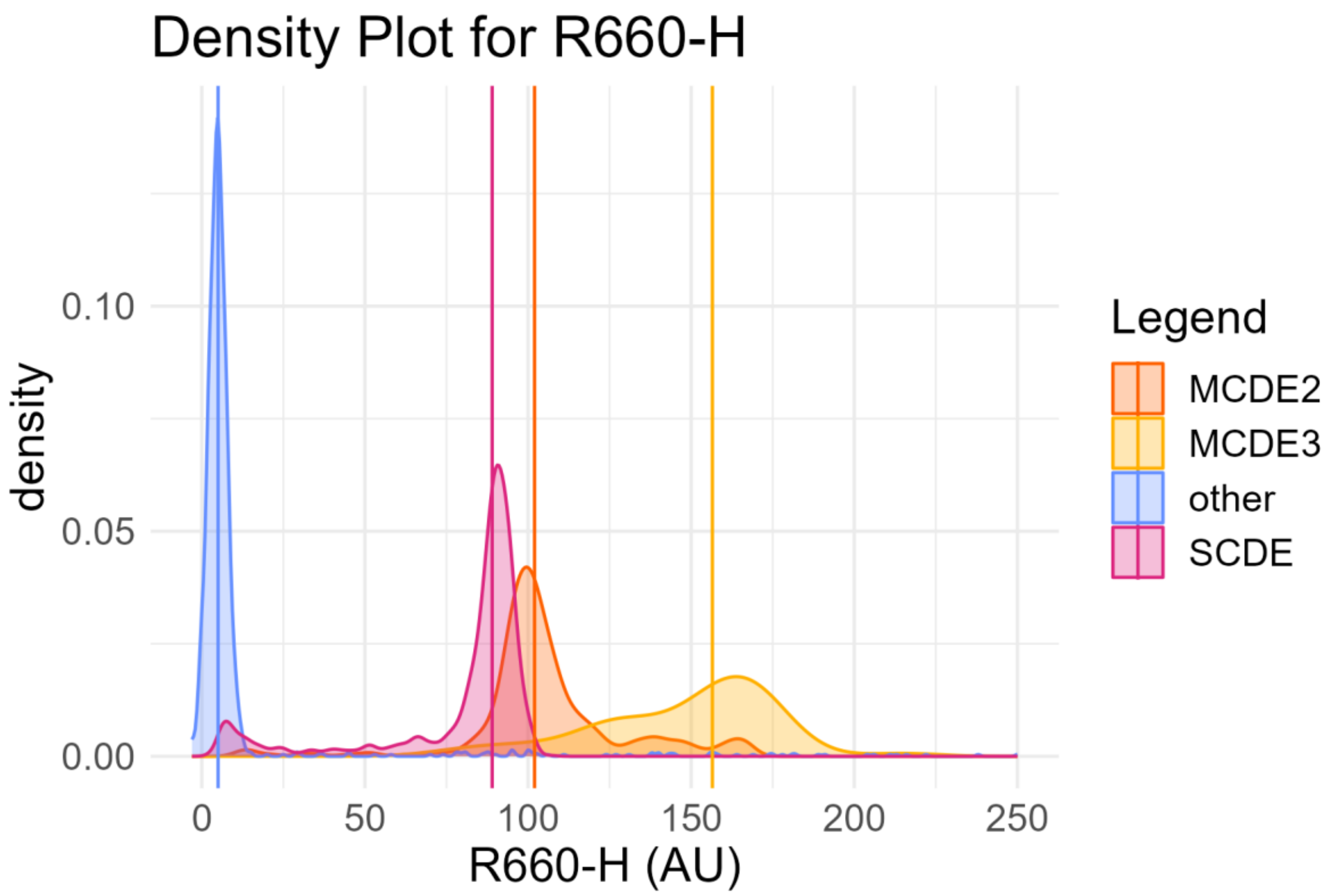

Fig. DD Density plots of Cy5 fluorescent-channels of all events in the high multicore scenario. These plots allow assessing qualitative clustering performance for a single parameter. Events are coloured by morphology. Magenta: single core double emulsions (SCDE), orange: double core double emulsions (MCDE2) and yellow: triple core double emulsions (MCDE3). Lines are drawn for the population medians in the corresponding colours.

Results part 3

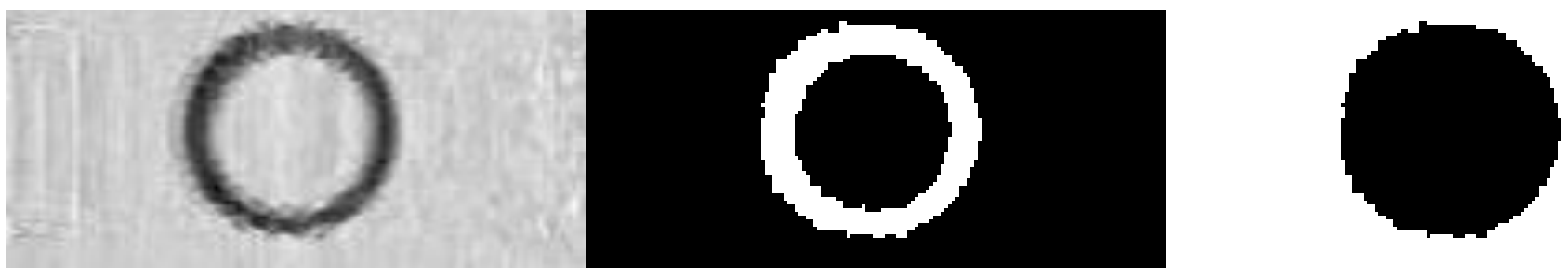

Fig. EE Calculating core size and oil layer size from imaging flow cytometer images. Black areas are calculated from the two binary images. Core size is equal to the black area inside

the droplet in the middle picture. Oil layer size is the white layer resulting from subtraction of black area in the rightmost picture and the core size derived from the middle picture.

Table D This table demonstrates that relative frequencies of different droplet fractions are the same between flow cytometry (FCM) and microscopy data. This serves as validation for the approach described in Materials and methods – Microscopy. Due to the low quality of microscopy images, accurate counting of broken cores was not feasible; therefore, imaging FCM data was used.

Droplet size (pL)	Volume buffer (mL)	Vortex speed (rpm)	Replicate	FCM-calculated uniformity (%)	Microscope-calculated uniformity (%)	Ratio
6	1,5	2500	1	89,51	86,35	0,96
6	1,5	2500	2	83,91	87,22	1,04
6	1,5	2500	3	87,12	81,61	0,94
6	4	2500	1	78,34	81,36	1,04
6	4	2500	2	77,47	85,60	1,10
6	4	2500	3	83,72	88,03	1,05
24	1,5	300	1	96,52	99,07	1,03
24	1,5	300	2	99,67	97,01	0,97
24	1,5	300	3	99,63	98,00	0,98
24	4	300	1	93,81	83,70	0,89
24	4	300	2	91,28	88,86	0,97
24	4	300	3	92,34	91,46	0,99

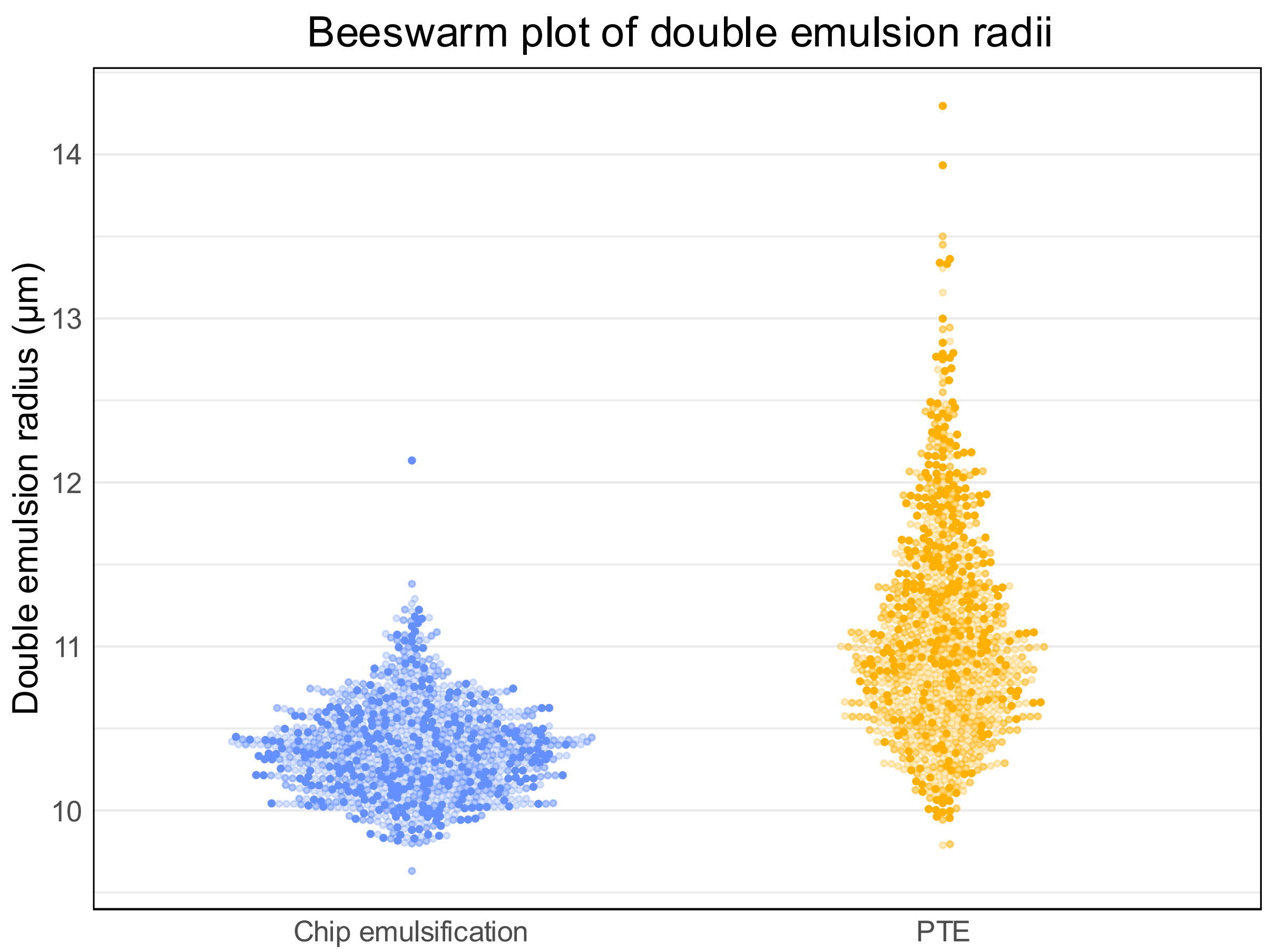

Fig. FF Beeswarm plot of the radii of single-core double emulsions y-axis is length in µm. Colours indicate different methods, colour shade each indicate one of three technical replicates (n = 306).

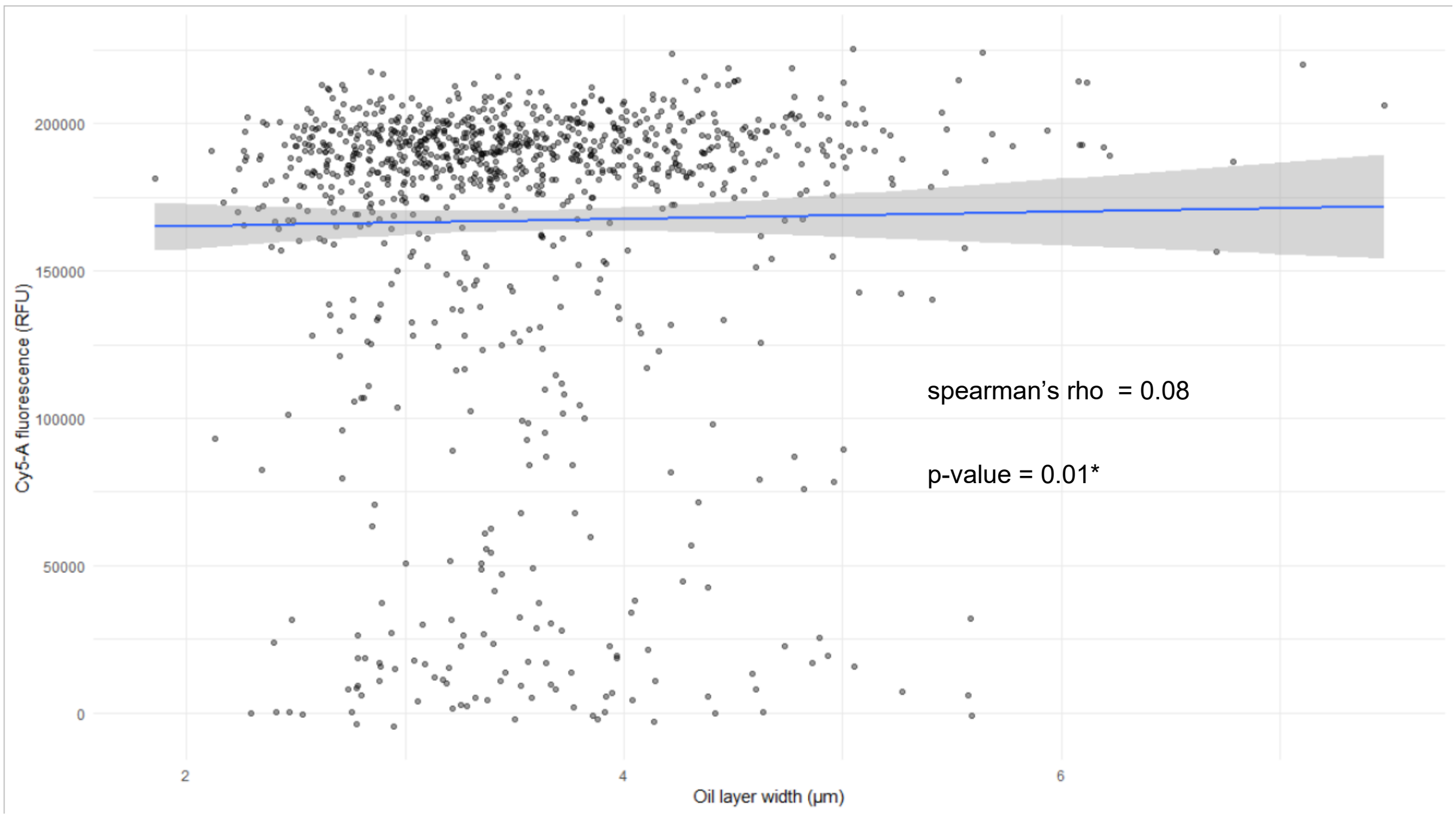

Fig. GG Scatter plot of SCDE events. X-axis is oil layer width in µm vs y-axis Cy5-A fluorescence (relative fluorescent units, RFU) of SCDE of all three DTE-replicates. There is a very weak significant correlation between fluorophore signal and oil layer thickness. A population is visible underneath the mean population with reduced Cy5-A fluorescence (<150 000 RFU). Looking at the iFCM images, these droplets were in-focus and showed normal scatter signals, making detection artefacts unlikely. They were also represented in the three replicates and in both methods (chip and DTE). The phenomenon behind this it is likely transport of sulfoCy5-COOH (the used fluorescent dye) out of the double emulsion which was observed when incubating the double emulsions over longer periods of time (hours). Why only a fraction of the population displays this behaviour is unclear.

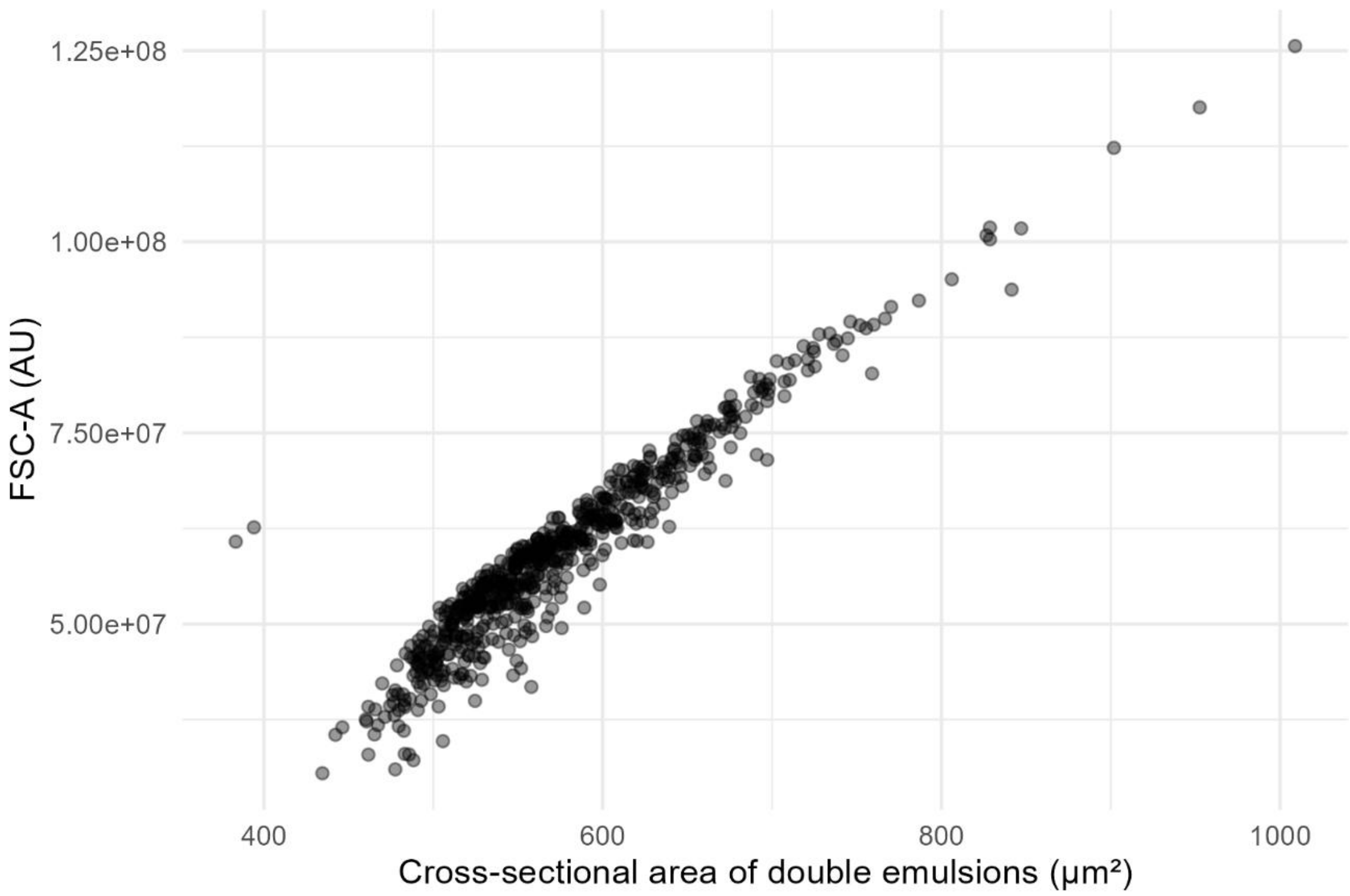

Fig. HH Scatter plot of cross-sectional area of double emulsions in µm² vs FSC-A signal (AU) of SCDE of all three DTE-replicates. This figure illustrates the linear correlation between the size of double emulsions and their forward scatter measurements, demonstrating the fundamental principle in flow cytometry that object size is proportional to forward scatter.[11]

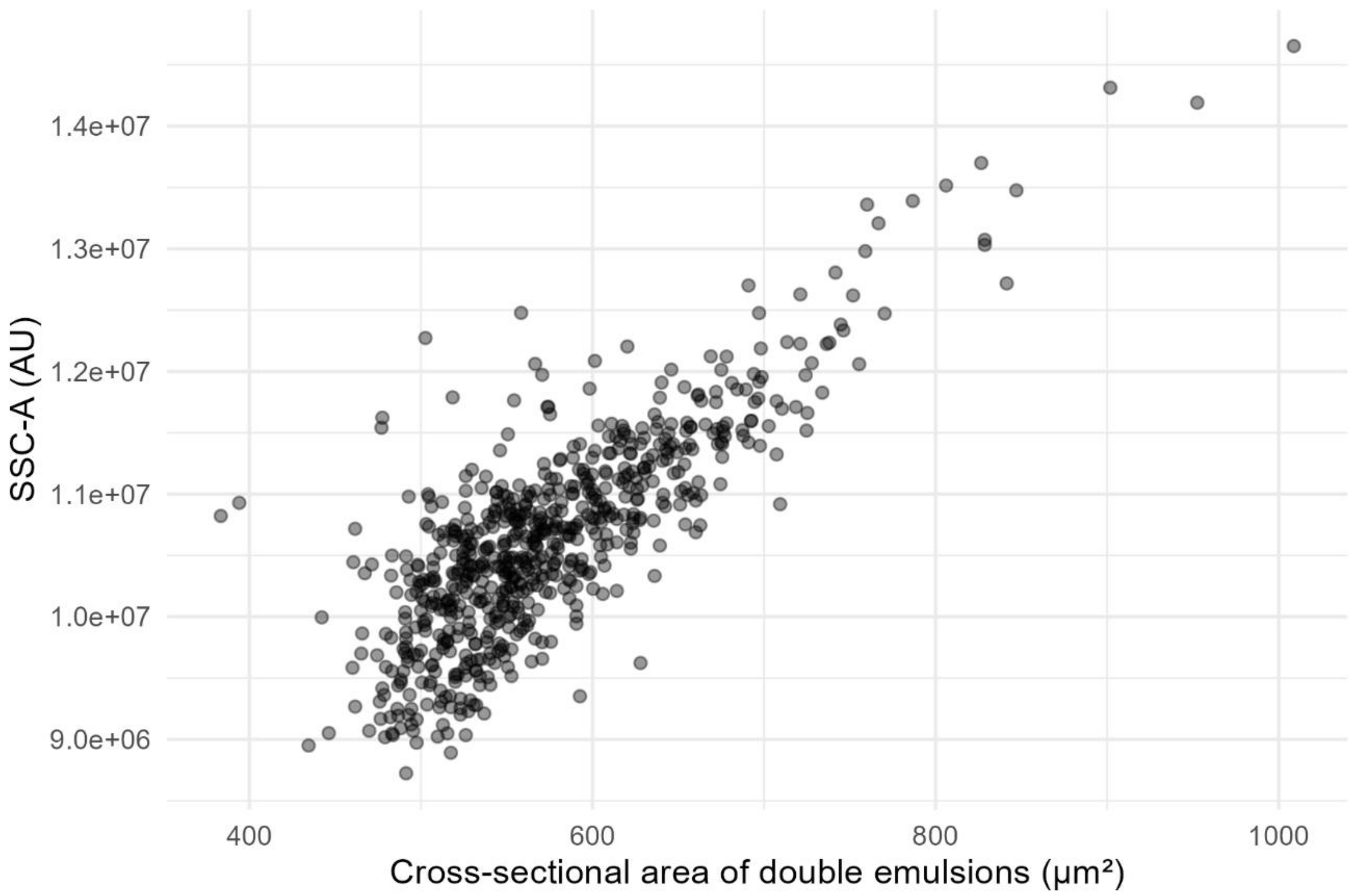

Fig. II Scatter plot of cross-sectional area of double emulsions in µm² vs SSC-A signal (AU) of SCDE of all three DTE-replicates.

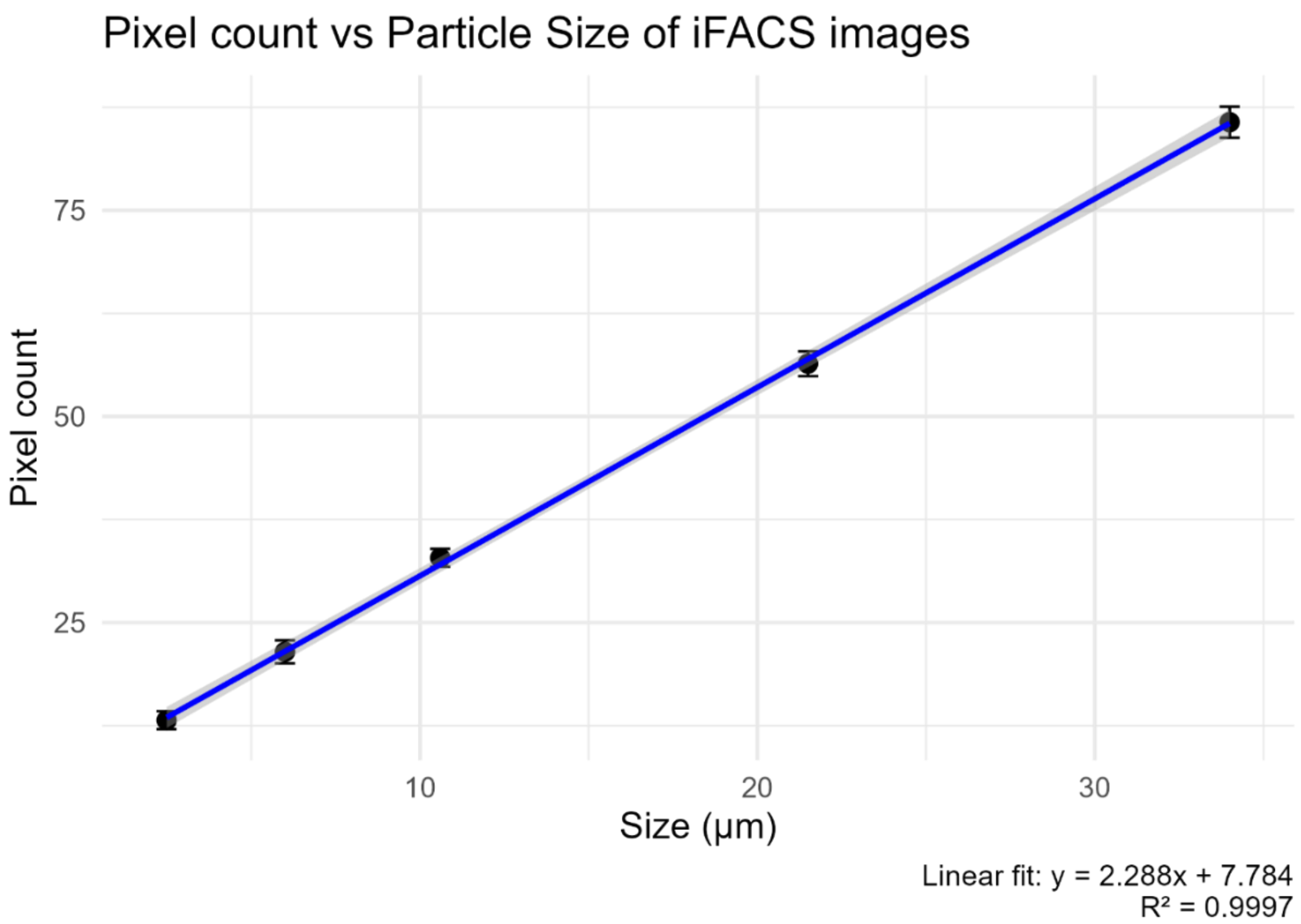

Fig. JJ Calibration curve for iFACS image analysis showing the relationship between particle size and measured pixel count on iFACS images. Calibration beads of 2.6 μm, 6 μm, 10.6 μm, and double emulsions of 21.5 μm and 34 μm diameter were used to generate the calibration line. The solid line is a linear regression fit. Equation and R-squared are shown on the bottom right.

Results part 4

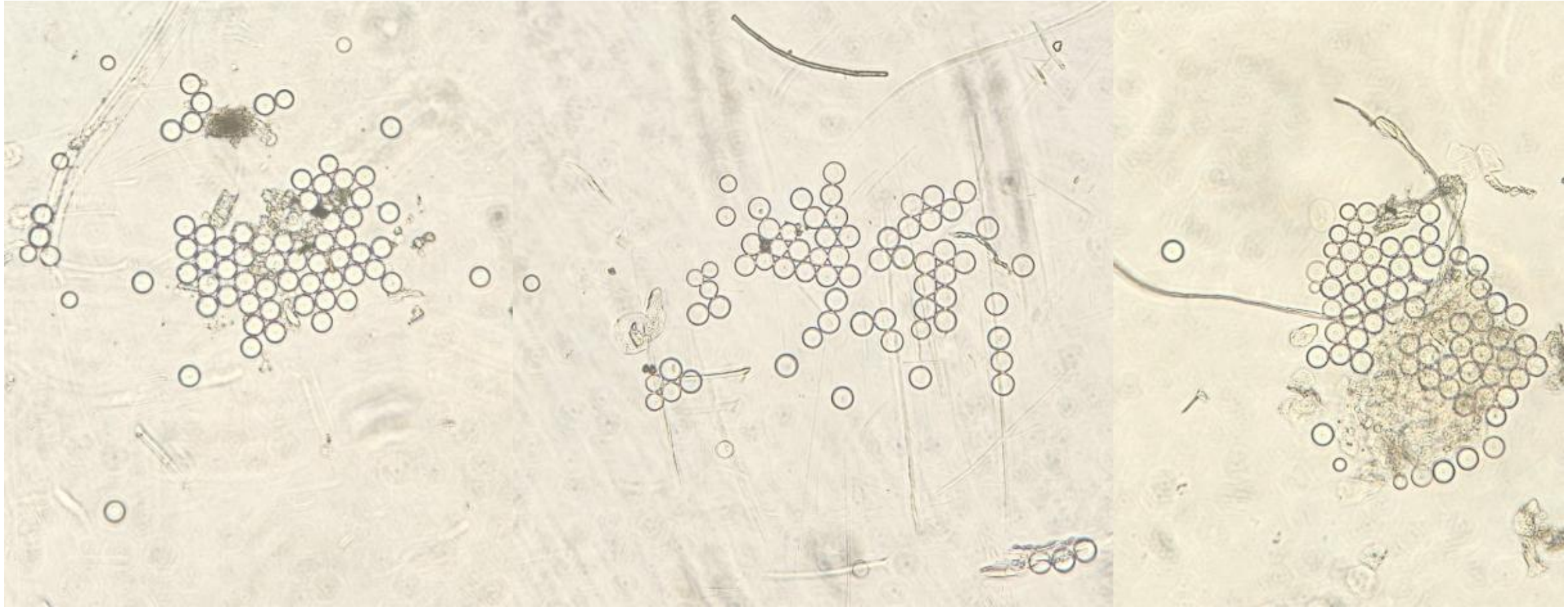

Fig. KK Population 1 SCDE sorted population. Foreign objects are dust particles. Optical imperfections of the 96-well plate bottom are also visible as out-of-focus lines in the field of view.

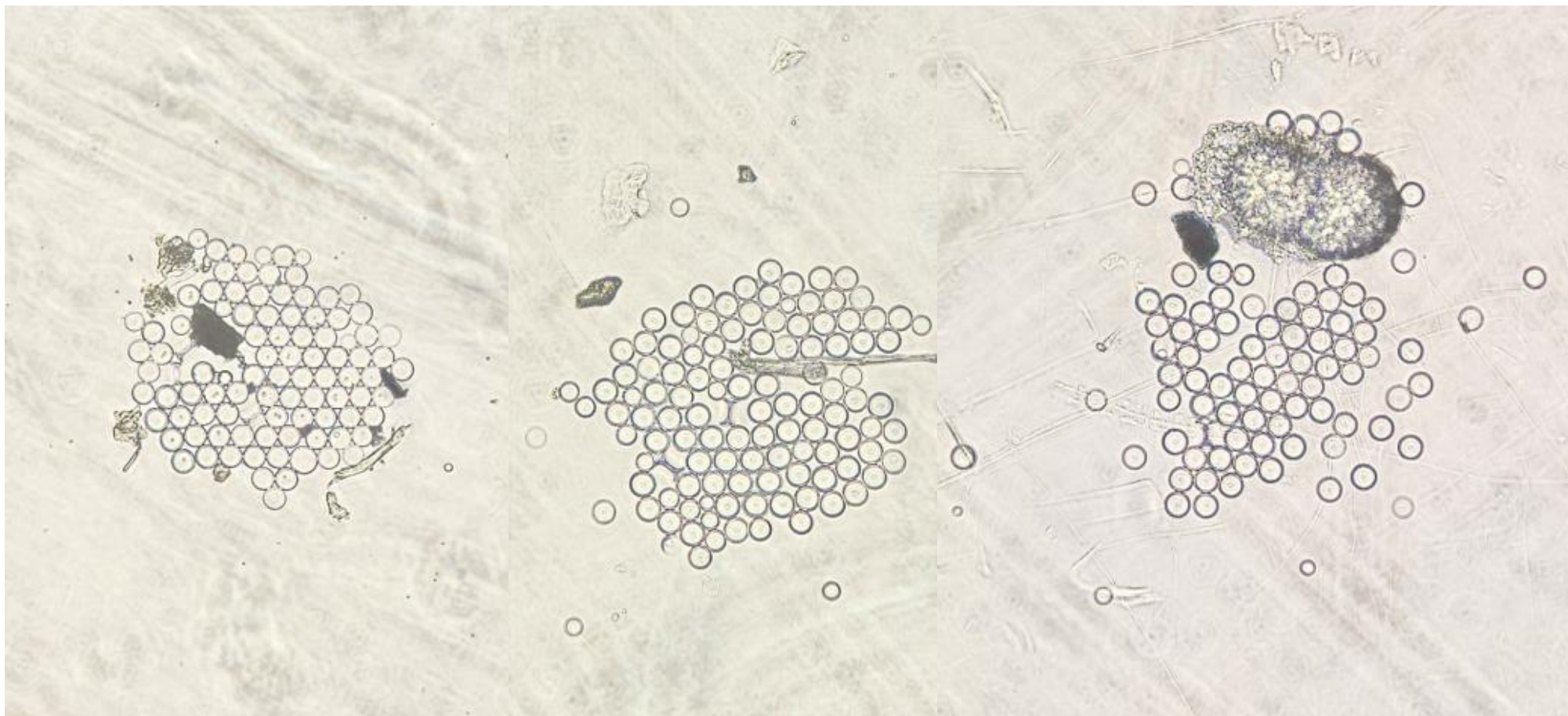

Fig. LL Population 2 SCDE sorted population. Foreign objects are dust particles. Optical imperfections of the bottom of the 96-well plate are also visible as out-of-focus lines in the field of view.

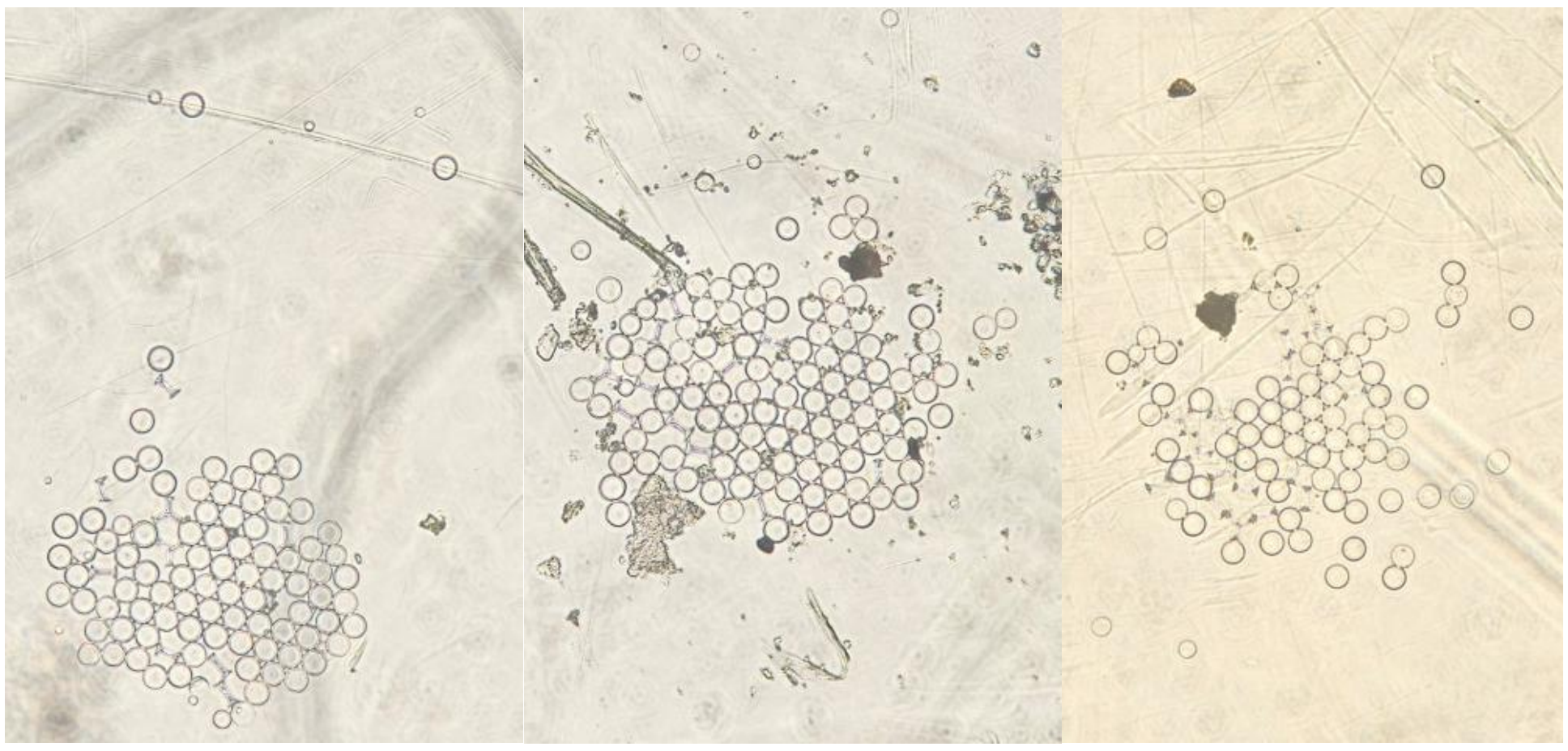

Fig. MM Non-stringent gating increases the fraction of MCDE that end up in the sorted fraction. Optical imperfections of the bottom of the 96-well plate are also visible as out-of-focus lines in the field of view.

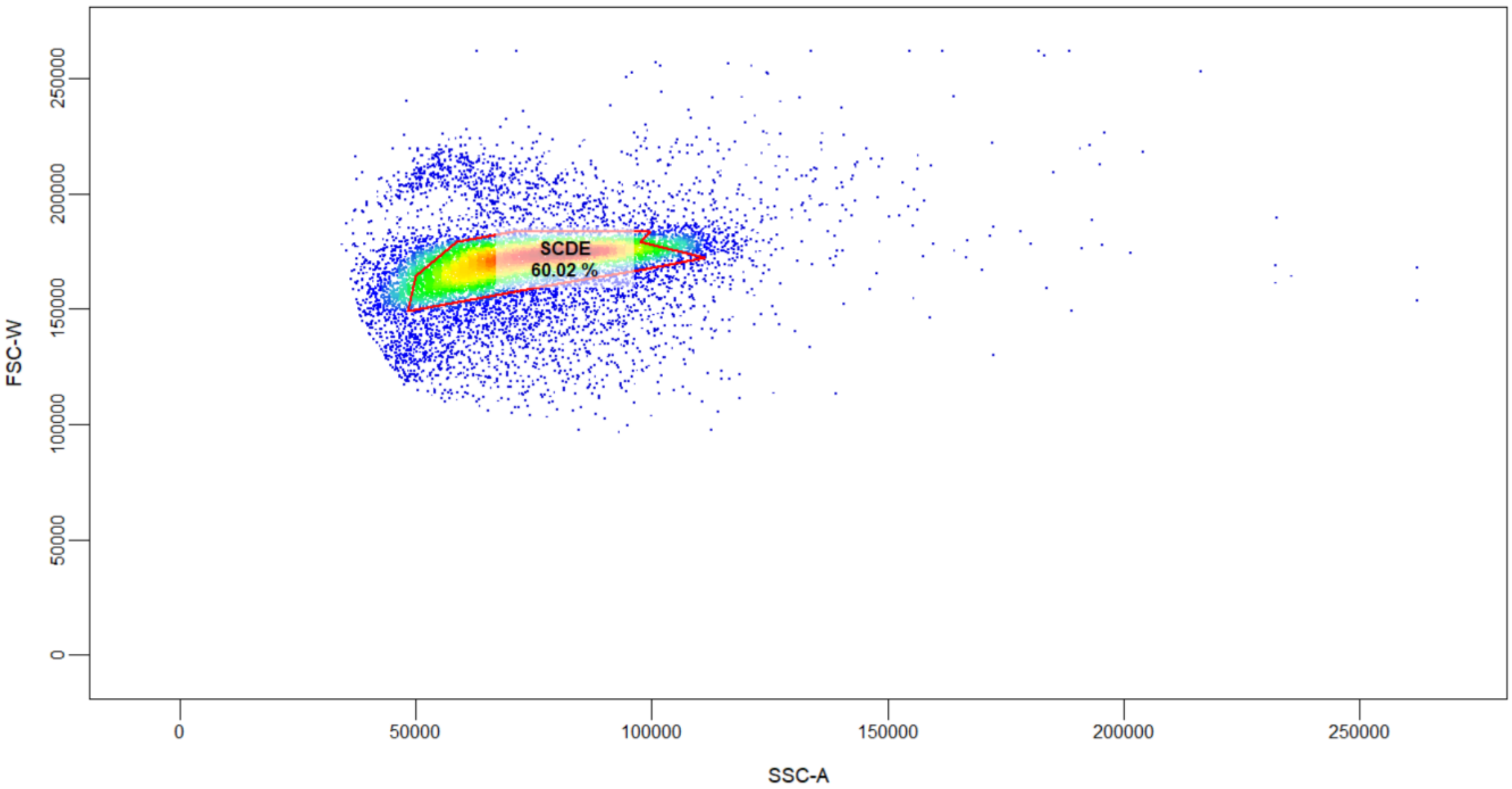

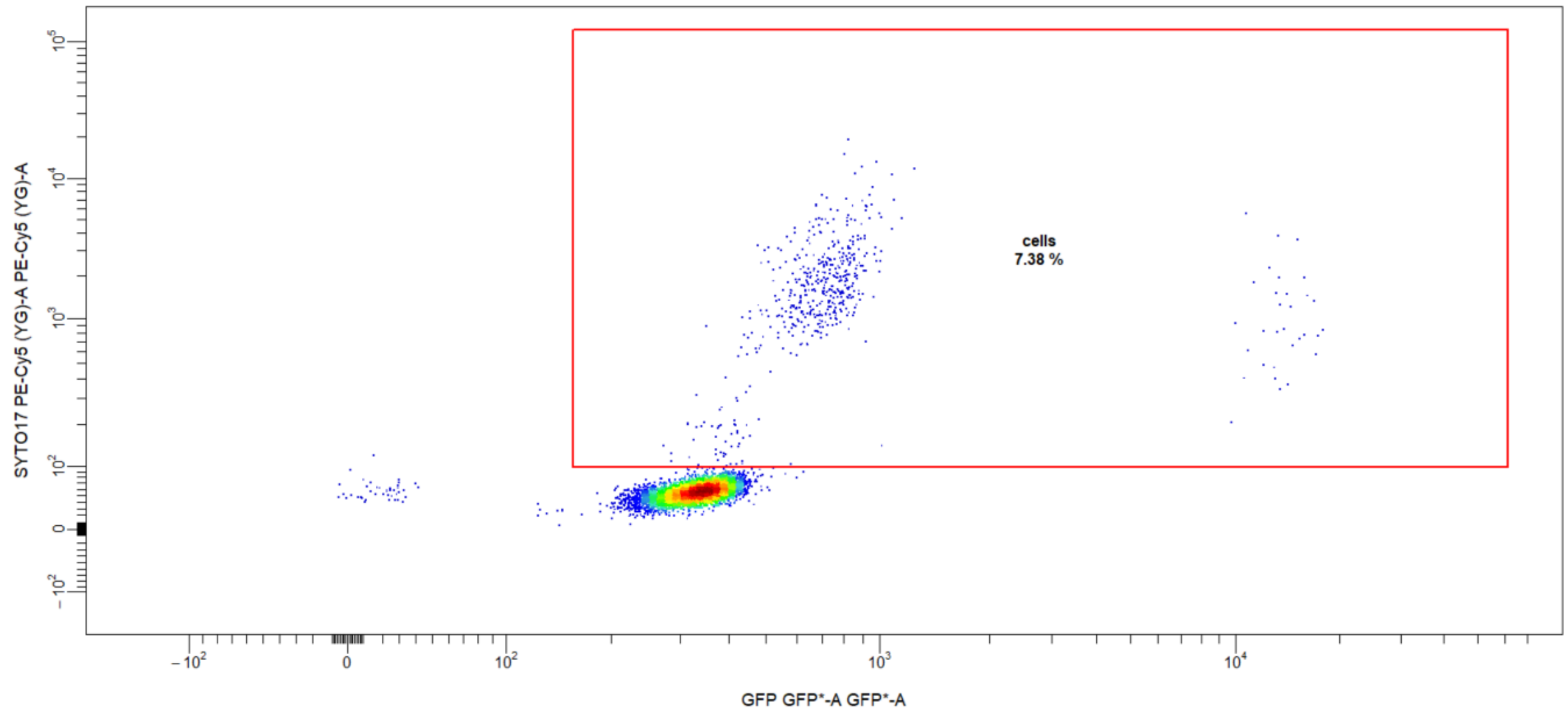

Fig. NN The upper plot illustrates the gating of single core double emulsions (SCDE) of Population 1 (results part 5). The gated SCDE are subsequently plotted in the bottom plot, which shows the gating for biomass-positive droplets.

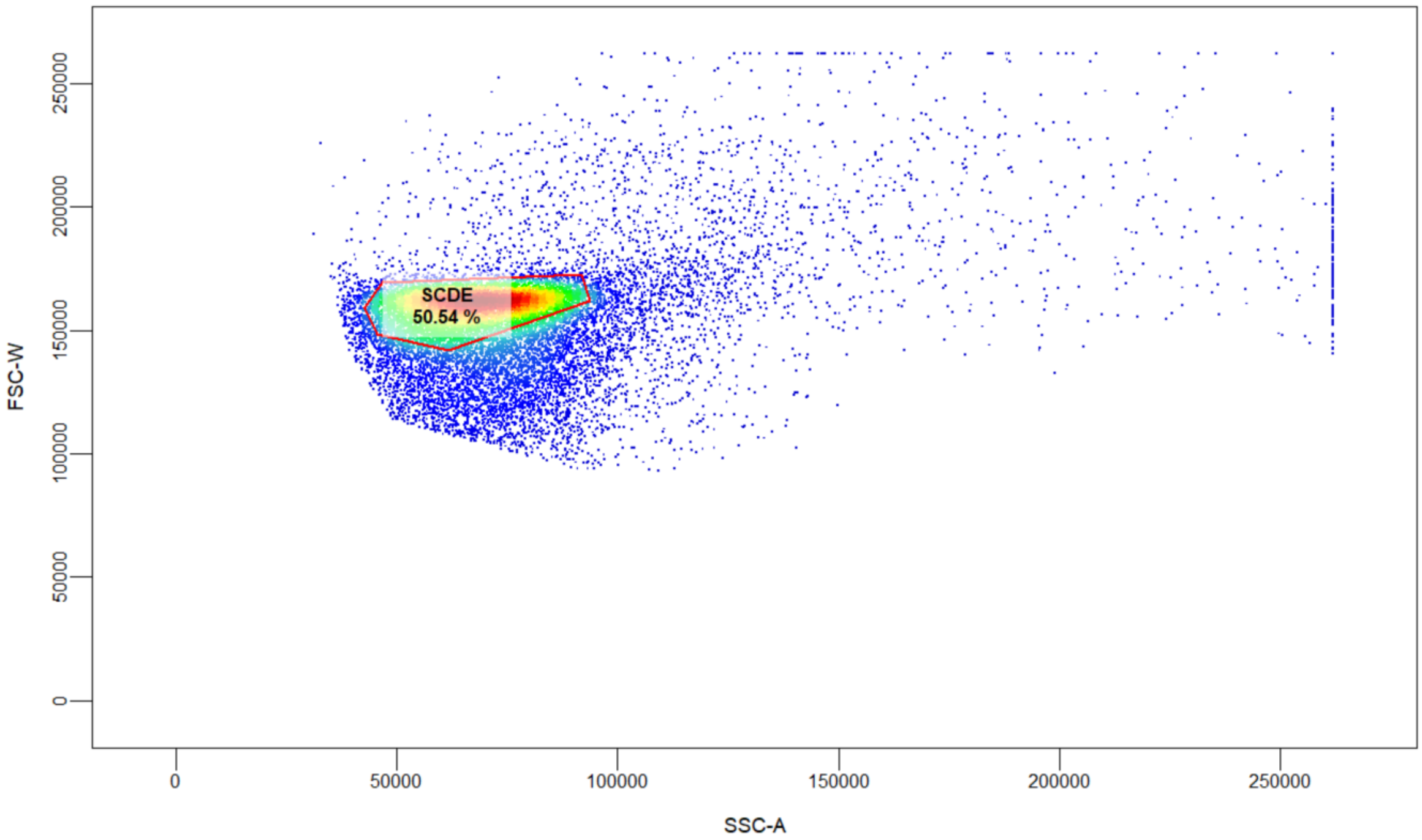

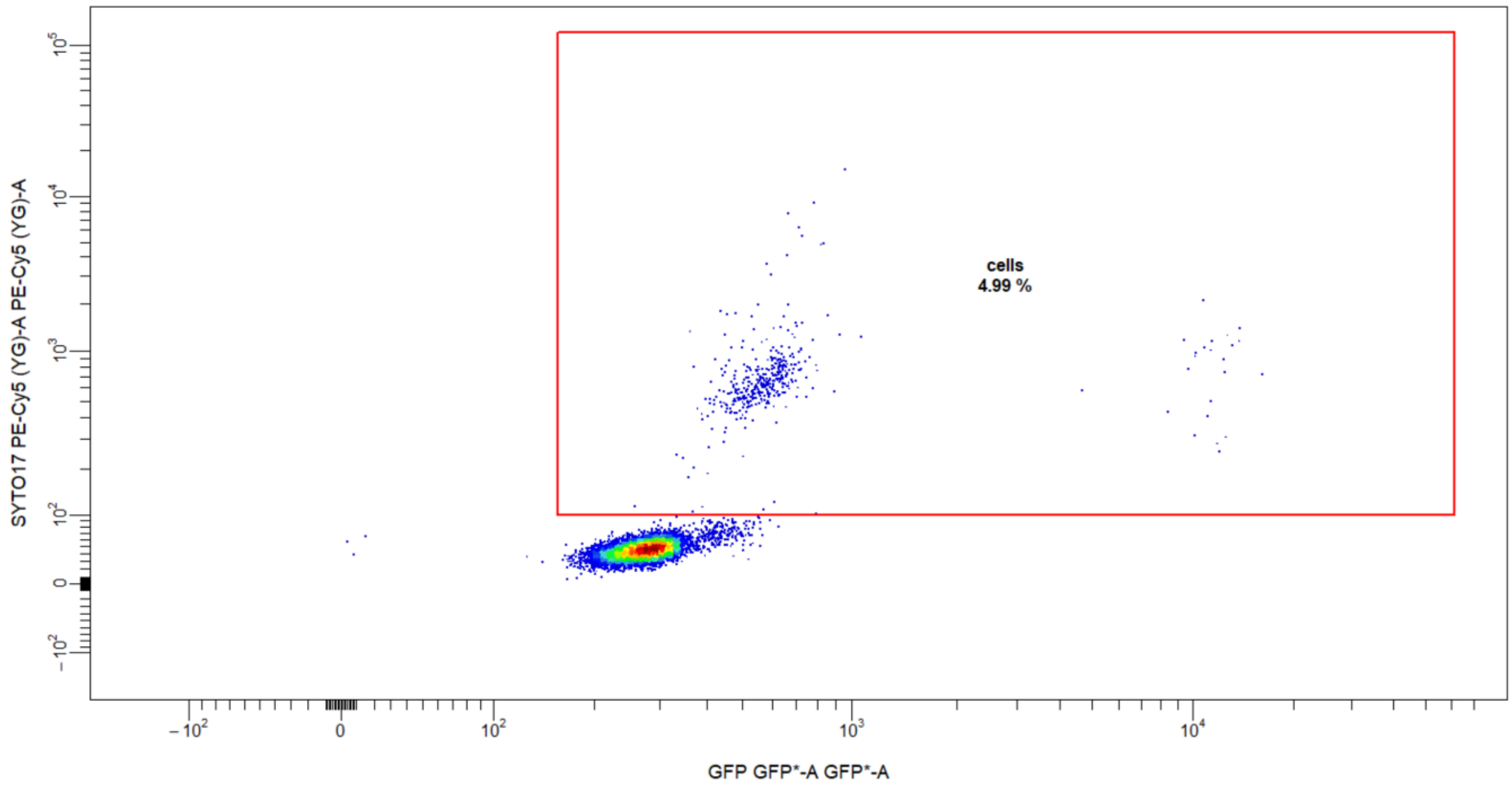

Fig. OO The upper plot illustrates the gating of single core double emulsions (SCDE) of Population 2 (results Part 5). The gated SCDE are subsequently plotted in the bottom plot, which shows the gating for biomass-positive droplets.

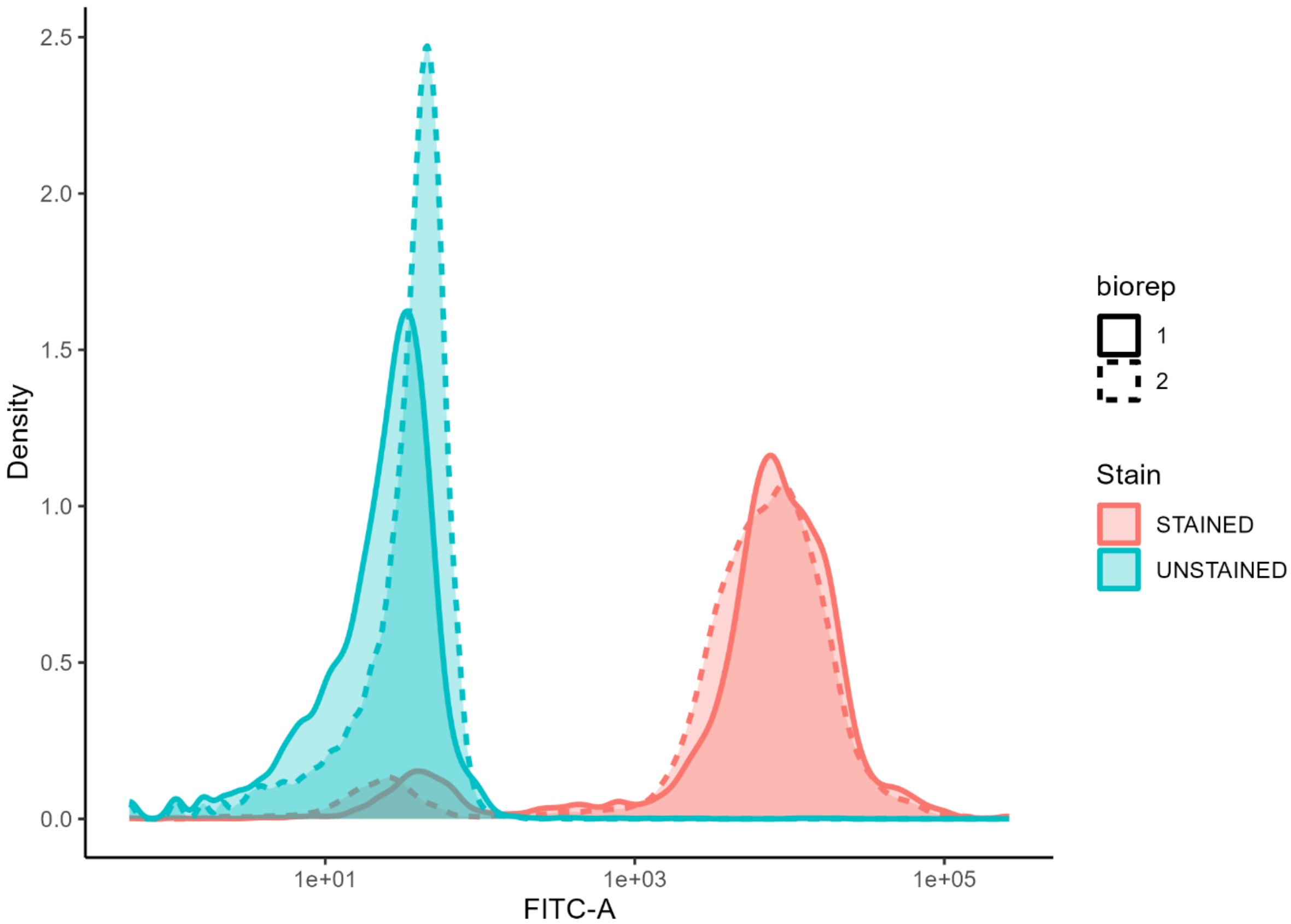

Fig. PP FITC-A signal distribution of stained versus unstained double emulsions. Density plots show the distribution of FITC-A fluorescence for gated single-core double emulsions. Stained and unstained populations are overlaid for hydrogen-oxidizing community coculture conditions, with fluorescence intensities displayed on a logarithmic scale. Data are shown separately for each biological replicate to assess reproducibility of staining and background signal.

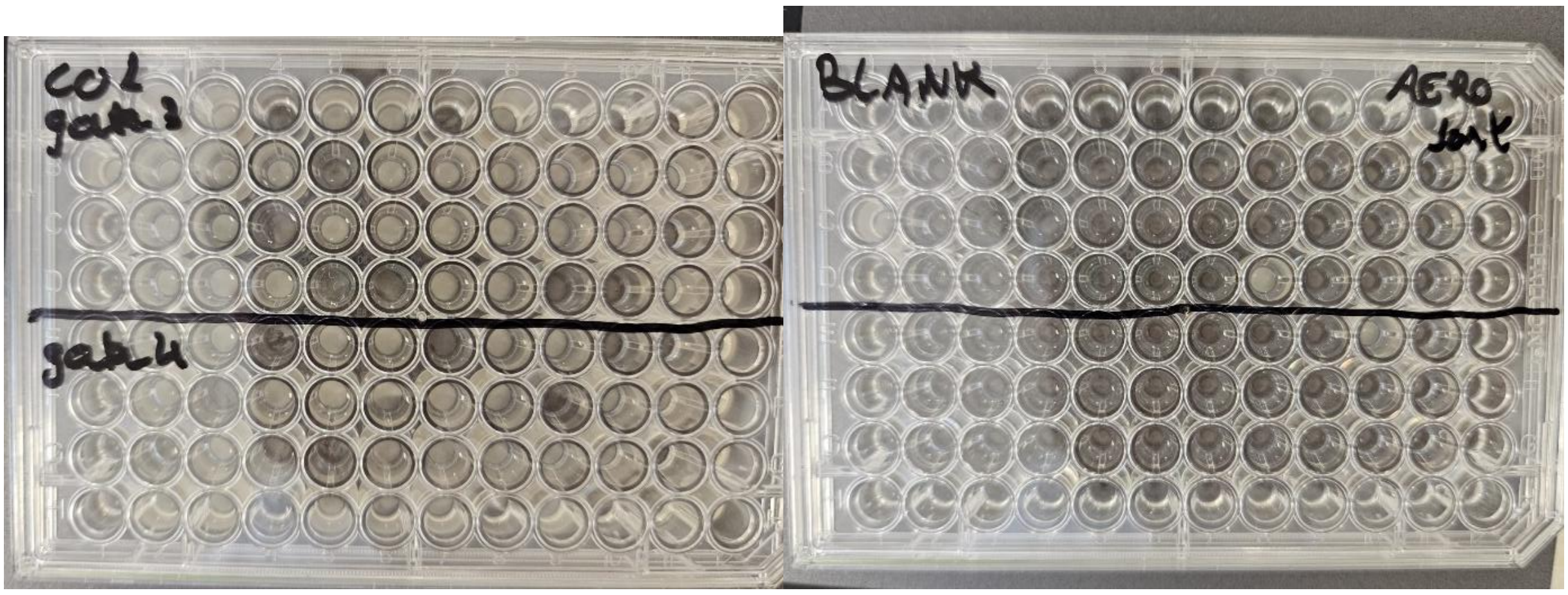

Fig. QQ Growth outcomes following sorting and plating of coculture droplets. In the coculture condition, 66 out of 96 wells showed growth. In the blank control, 3 out of 96 wells exhibited

growth in rich medium, likely reflecting low-level contamination associated with the non-sterile nature of the FACS sorting process.

Discussion

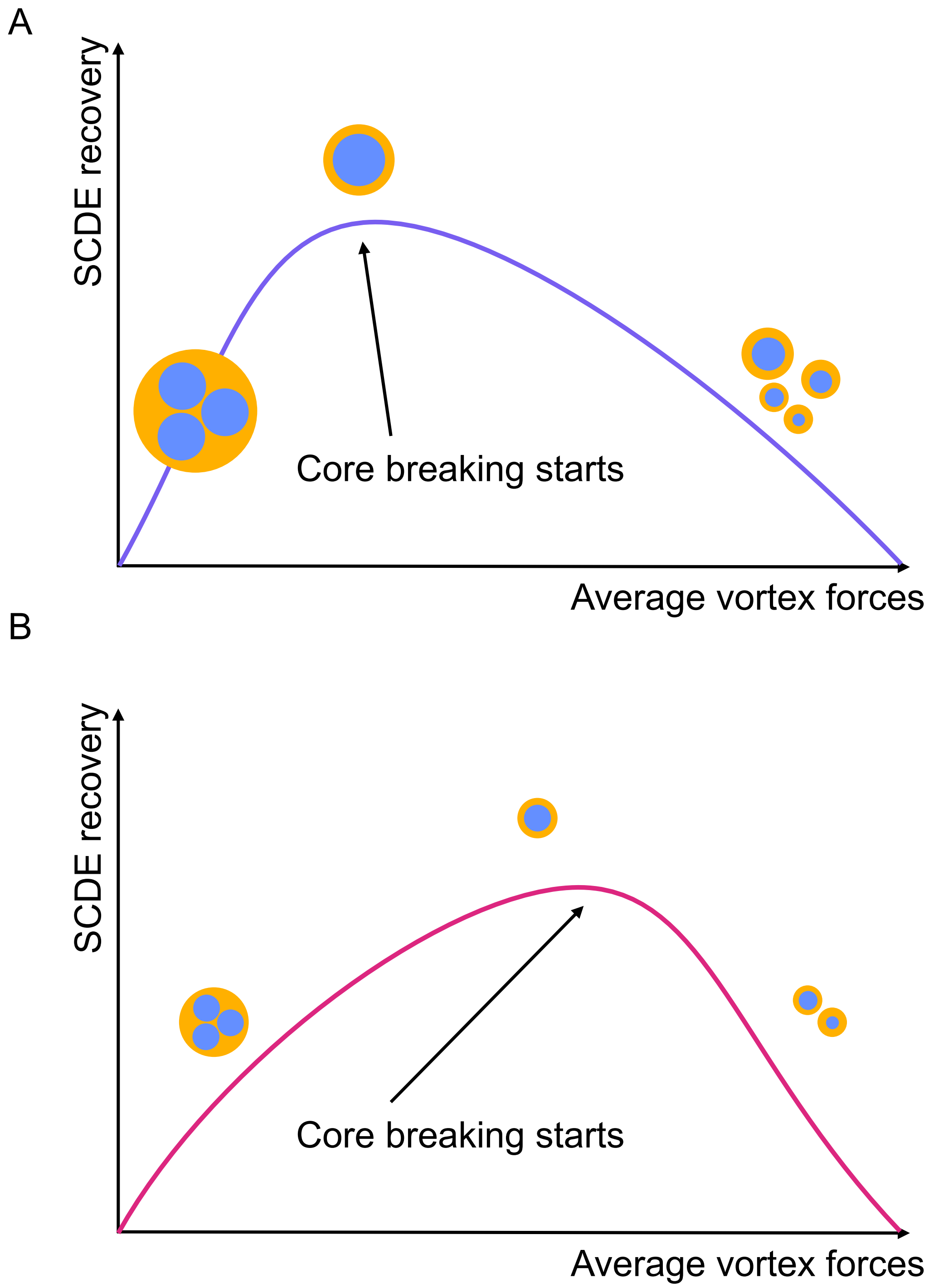

Fig. RR Qualitative representation of behaviour of conversion efficiency in function of average vortex forces (~vortex speed) for a large core droplet (A) and a small core droplet (B).

Substantial MCDE break-up into SCDE will happen at lower vortexing speeds for larger droplets than for smaller droplets. Increasing vortexing speed increases fraction of SCDE up until a point of core breaking dependent on droplet size, the larger the droplet, the earlier this happens.[9]

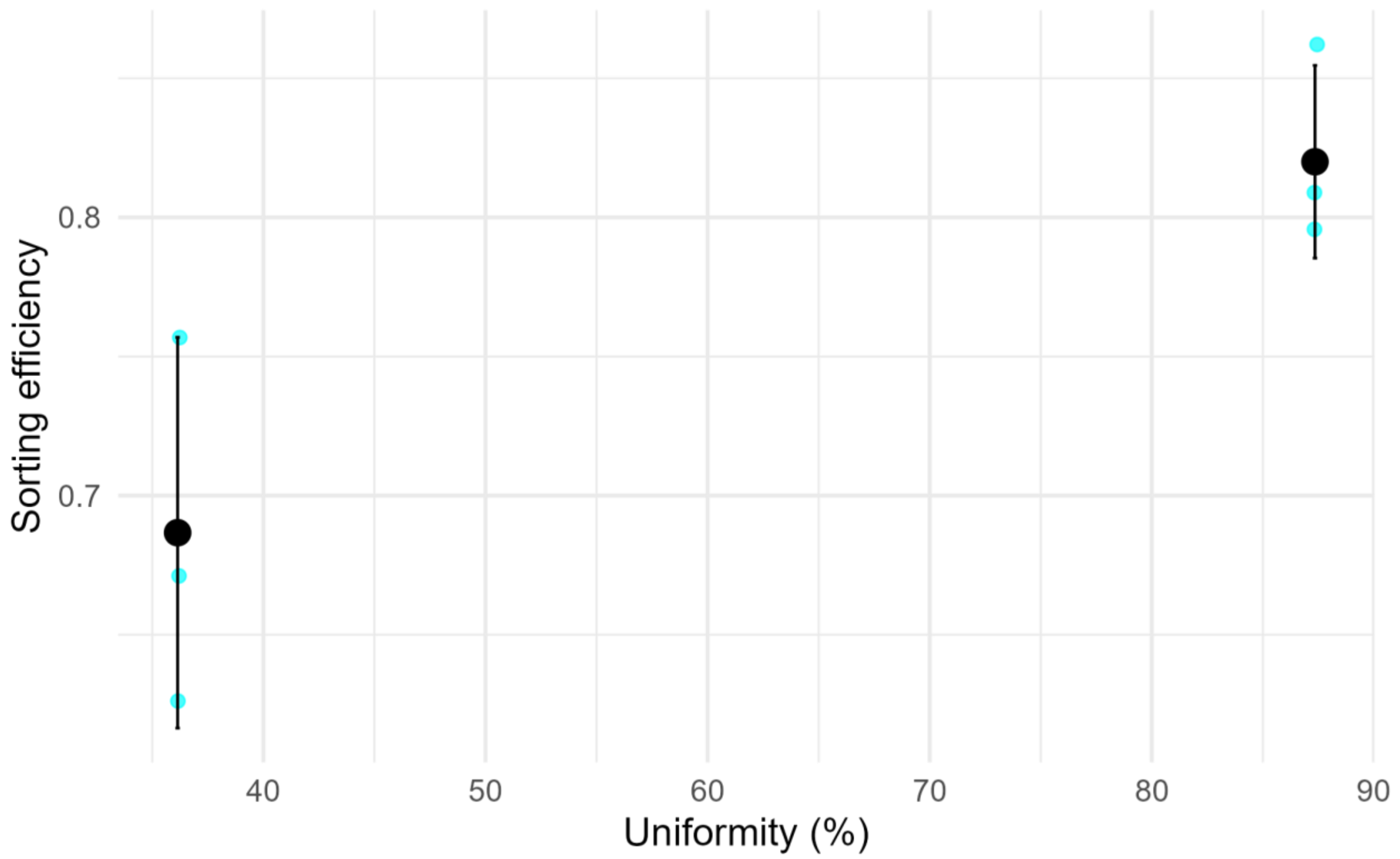

Fig. SS Sorting efficiency versus population uniformity of a 7 pL double emulsion population. Cyan dots are individual technical replicates (n = 3), black dots with error flags are average and standard deviation of these individual replicates.

Table E Fifty double emulsions were sorted into different sorting buffers. After sorting the remaining double emulsions were manually counted. Three technical replicates were sorted. In the end on average 82 % of the double emulsions were lysed when sorting in a medium without surfactant. 1x PBS + 1w% Tween 20 was the positive control, here it is assumed that no double emulsions break after sorting. Sorting in 1xPBS destabilizes the double emulsions and lyses them yielding a higher release of bacteria from microdroplets.

Sorting buffer	Replicate 1	Replicate 2	Replicate 3	Average
1x PBS + 1w% Tween 20	29	32	29	30
1x PBS	3	4	9	5.33
Average lysis %				82.22%

Supplementary information - Extended materials and methods

Single emulsion production

Water-in-oil emulsions were generated using two syringe pumps (TSE systems type 540060, Berlin, Germany). Syringes were connected to the PTFE tubing (1/16” OD, 0.3 mm ID, Deutsch & Neumann, Hennigsdorf, Germany) using Luer lock-HPLC fittings (IDEX Health and Science, Oak Harbor, USA). The aqueous phase varied depending on the population made. The standard flow rates used were 250 µL/hr aqueous phase and 300 µL/hr oil phase to decrease droplet size oil flow rate was increased. Microfluidic operations were monitored using an inverted microscope (Zeiss Axiovert 135) and footage was recorded using a high-speed camera (S-Motion Mono 2.6 GB High Speed Camera, AOS Technologies AG, Dätwill, Switzerland). All droplet populations around 4-10 pL in volume were made using channels of 10 µm height. For droplets around 20-35 pL in size, channels were used that are 20 µm in height.

For all droplet samples except the synthetic community sample, the oil phase consisted of 2.5 w% Ionic PEG-Krytox (Krytox 157 FSH, Chemours, Wilmington, USA) in HFE-7500 (3M, Zwijndrecht, Belgium). The oil phase of the caproic acid reactor sample consisted of 1 w% Pico-Surf (Sphere Fluidics, Cambridge, UK) in HFE-7500 to avoid biocompatibility issues that might arise with the ionic Krytox surfactant.[12,13]

Double emulsion production on chip

Double emulsions were generated by reinjecting single emulsions in a hydrophilic chip with channels with 10 µm height. Three syringe pumps were used to control the flow rates of the droplets, the outer aqueous phase and the spacer oil. To set up stable double emulsion production spacer oil consisting of 2.5 w% Krytox 157 FSH in HFE-7500 was flowed through the chip at 300 µL/hr together with droplets at 250 µL/hr to allow stabilisation of droplet reinjection. After stabilization, spacer oil is displaced by aqueous buffer (2 w% Tween 20 in 1x PBS (Sigma, Overijse, Belgium)) by rapidly swapping tubing and flowing aqueous buffer at 500

µL/hr through the chip to allow HFE vacation and initiation of double emulsion production. The aqueous buffer flow rate is then reduced until SCDE production is observed.

Microfluidic device fabrication

Microfluidic channels were designed in AutoCAD 2022 (Autodesk Inc., USA). Microfluidic silicon wafers were made using standard photolithography techniques with a 10 µm and 20 µm relief height. PDMS (Sylgard 184, Dow, Midland, USA) was mixed at 10:1 mass ratio with the supplied curing agent after which it was poured on top of the SU-8 mould. The PDMS mixture was degassed by repeatedly applying and releasing a vacuum. The PDMS was cured by baking at 60 °C overnight. The cured PDMS was carefully removed from its mould, after which channels were cut out and inlet and outlet holes were made with a 1.25 mm biopsy punch (WPI, Worcester, USA). The punched holes were cleaned with isopropanol (Carl Roth, Karlsruhe, Germany) and dried with pressurized air. Dust and small particles were removed from the chip surfaces and microscope glass slides (Carl Roth, Karlsruhe, Germany) using Scotch tape (3M, Zwijndrecht, Belgium). Chips and microscope slides were treated in a plasma oven (Femto Plasma System, Diener Electronic, Ebhausen, Germany) with oxygen plasma for 12 seconds at 100 W power, 20 sccm O_2 flow rate and 0.65 mbar pressure. PDMS chips and glass slides were gently brought together. Successful bonding was confirmed by attempting to pry away the PDMS from the slide until it resisted separation. To make hydrophobic chips Novec1720 (3M, Zwijndrecht, Belgium) was added to the channels using a micropipette. The carrier liquid was then left to evaporate at room temperature in a desiccator for 15 minutes or until no more liquid was visible in the channels after which the chips were baked at 135 °C for 15 minutes. To make hydrophilic chips microscope slides were spin coated with PDMS by adding 500 µL degassed PDMS mixture and spun 30 seconds at 500 rpm, 1 minute at 1500 rpm (SPIN150i, POLOS, Putten, Netherlands) after which they were baked overnight at 60 °C. Plasma treatment and bonding happened as previously described above. Immediately after successful bonding is determined, a 0.22 µm-filtered 20 g/L polyvinyl alcohol (PVA, 87-90% hydrolysed, average molecular weight 30 kDa-70kDa, Sigma, Overijse, Belgium) solution was

added to the channels. The PVA solution was left for at least 3 minutes after which it was removed by applying a vacuum to the outlet channel. Excess PVA solution was removed from the channels by flushing with 0.22 µm-filtered ultrapure water after which the chips were baked at 135 °C for 15 minutes. Scotch tape was used to close off the inlet of treated chips, after which they were stored at room temperature. For microscopic observation of water-in-oil emulsions, a droplet observation chamber was manufactured by pouring PDMS over a cover slip.[14] This PDMS chamber was then degassed, baked, cut out, punched, plasma treated and hydrophobized as mentioned above. However, rather than a normal microscope slide, a large cover slip (50*22 mm, Fisher Scientific) was used as a bonding surface for the PDMS.

Construction of the two-species synthetic community

C. nitrativorans 23310 and *E. coli* MG1655 Y3 were streaked from a glycerol stock on an LB agar plate (Luria/Miller, Carl Roth, Karlsruhe, Germany). They were incubated at 37 °C and regularly checked until colonies were visible. Single colonies were picked from both species and 1 mL cultures were incubated in biological duplicate from the morning in LB (Luria/Miller) to the evening at 37 °C. Dilutions from 10^{-1} to 10^{-5} were made. *C. nitrativorans* was incubated at 28 °C and *E. coli* was incubated at room temperature overnight. The overnight dilutions were judged by eye on their growth phase and exponentially growing cultures were selected for both duplicates of both species. Cell numbers were determined in technical triplicate using an Accuri C6 Plus flow cytometer (BD, 2 laser). Cell suspensions were diluted 1000-fold in 0.2 µm filtered 1x PBS and stained using SYBR® Green I nucleic acid stain (1X final concentration, Fisher Scientific, Merelbeke, Belgium). After dilution, 25 µL sample was analysed with the fast flow rate settings. Thresholding was done on the FITC channel, disregarding events smaller than 500 relative fluorescent units (RFU). The original cell suspensions were kept on ice during the analysis and after obtaining cell counts the synthetic community was assembled. The final composition of the synthetic community was $9*10^6$/mL *C. nitrativorans* 23310 and $1*10^6$/mL *E. coli* MG1665 Y3.

Imaging flow cytometry on Vulcan (BD)

Double emulsions were loaded and analysed on a BD Vulcan™ flow cytometer (flow cytometer with BD Cellview™ and a 3 laser, 10 colour configuration). Voltages were initially adjusted so that the majority of the analysed population was visible on the scatterplots and were kept the same between experiments. Next to standard flow cytometric parameters (scatter, fluorescence) images were also obtained for each event using BD Cellview. For each double emulsion population data of 3000 events were collected. Linearity of object size on imaging FACS pictures was confirmed by running calibration beads and emulsions of known size (Fig. JJ).

Microscopy

All single emulsion populations were imaged in a droplet chamber. First, the droplet chamber was filled with HFE-7500, after this, about 2 µL single emulsions were added to the droplet chamber. PDMS plugs were used to plug the two chamber outlets. The chamber was incubated on its side for 2 minutes to allow a droplet monolayer to form. The chamber is then inserted on the platform with the coverslip facing up. To image double emulsions, 10 µL 1x PBS + 2 w% Tween20 buffer was added on a microscope slide. After this, the double emulsions were aspirated and submersed in the liquid phase on top of the microscope slide. Pictures were taken with an Axioskop 2 plus (Zeiss) equiped with a QImaging Retiga R3 CCD Camera (Cairn Research, Faversham, USA).

To calculate conversion efficiency, samples were imaged in a 96-well plate filled with 100 µL 1x PBS + 2 w% Tween 20 using an Primo Vert inverted microscope (Zeiss) fitted with an infinity 1 P95-C camera (Lumenera, Ottawa, Canada). The plate was incubated on its edge for 5 minutes to let the double emulsions migrate to one side of the well after which pictures were taken. The images exhibited insufficient quality for automated image analysis, so double emulsions were manually counted using Fiji software. For samples with broken core double

emulsions, both SCDE and smaller double emulsions were counted. Imaging flow cytometry data of the same samples was utilized to calculate counts specifically for SCDE (1)

$$C_{SCDE} = \frac{C_{SCDEandSmaller,BF}}{f_{SCDEandSmaller,FCM}} * f_{SCDE,FCM} \ (1)$$

(1) C_{SCDE} stands for SCDE concentration (counts/mL), $C_{SCDEandSmaller,BF}$ stands for the concentration as counted on the brightfield microscope (counts/mL). $f_{SCDEandSmaller,BF}$ stands for the relative percentage of SCDE and broken core double emulsions that were smaller than modal SCDE, the percentage is relative to all objects measured during imaging flow cytometry. $f_{SCDE,FCM}$ stands for the relative percentage of SCDE and broken core double emulsions that were smaller than modal SCDE.

The imaging flow cytometry showed proportions of MCDE and SCDE consistent with the microscopy data (Table D), providing internal validation for this method. DTE conversion efficiency was calculated as follows:

$$Conversion\ efficiency = R = \frac{C_{SCDE,measured}}{C_{SCDE,theoretical\ max}} \ (2)$$

(2) C_{SCDE} stands for the empirically determined SCDE concentration (counts/mL). $C_{SCDE,theoretical\ max}$ stands for the theoretical maximal core concentration (counts/mL).

DTE uniformity was calculated as follows

$$Uniformity = Y = \frac{Amount\ of\ SCDE}{Amount\ of\ SCDE+MCDE} \ (3)$$

(3) Equation used to calculate DTE uniformity.

With the formula for theoretical maximal core concentration being:

$$Theoretical\ maximal\ core\ concentration = \frac{V_{added} * \delta}{V_{single\ emulsion} * V_{OA}} \ (4)$$

(4) δ is the packing factor of the water-in-oil emulsions that are added to the DTE batch, considered to be 0.63,[15] and V_{added} is the volume that is added $V_{single\ emulsion}$ is the volume of a single emulsion. V_{OA} is the volume of outer aqueous phase in the DTE batch.

Image analysis of imaging flow cytometric data

Single emulsions of 4 pL were subjected to DTE and chip-based emulsification in technical triplicate and analysed on the Vulcan imaging flow cytometer (BD) as described before. Data was collected for 3000 events. One thousand of these images were manually categorized into 4 categories for each replicate according to their morphology: single core, double core, triple core double emulsions, and others (oil droplets and some rare higher order core double emulsions). Only SCDE were considered for the analysis. For each image two features were extracted: the double emulsion area and the core emulsion area (Fig. EEI).

Images were processed using Fiji software.[16] To extract the double emulsion area Huang automatic thresholding method was applied to each image to segment the emulsion droplets. After thresholding, the images were converted to binary masks with a white background. Holes within the droplets were filled to ensure complete segmentation. The "Analyze Particles" function was then used to measure the properties of droplets, specifying a size range of 20 pixels to infinity. Results were saved as CSV files.

To extract the core emulsion area the Huang automatic thresholding method was applied to each image for initial segmentation. The images were then converted to binary masks with a white background. The “Close-“ function was used to remove noise after which the images were inverted. The "Analyze Particles" function was utilized to measure the properties of droplets, with parameters set to a size range of 20 pixels to infinity and a circularity range of 0.50 to 1.00. Particles outside these criteria were excluded from the analysis. Results were saved as CSV files.

Finally, the measured areas allowed calculation of the radius of the double emulsion and the core emulsion. The width of the oil layer was then determined by subtracting the core emulsion radius from the double emulsion radius from which an average width was calculated (Fig. EE).

$$w_{oil\ layer} = r_{double\ emulsion} - r_{core\ emulsion} \quad (5)$$

(5) $w_{oil\ layer}$ is the width of the oil layer, $r_{double\ emulsion}$ is the radius of the double emulsion, $r_{core\ emulsion}$ is the radius of the core emulsion.

Analysis of imaging flow cytometry data to determine optimal parameter pairs for gating

Using the categorized imaging flow cytometry data, we compared combinations of flow cytometric parameters for their ability to cluster the SCDE separately from the artefacts. Because this gating strategy has to be applied in real-time during the sorting process and most FCM-software does not support dimension reduction techniques, i.e. only allows plots for two parameters, only parameter pairs were considered. An optimal parameter pair will segregate the SCDE and the artefacts in well-defined clusters in completely different positions in the parameter space, allowing easy, accurate gating, i.e. high-quality. A bad performing parameter pair will not be able to segregate SCDE and artefacts, placing the population of SCDE and artefacts in similar positions in the parameter space in non-distinct clusters, i.e. low-quality clustering.

From the classified events, SCDE were selected and an automated gating algorithm was applied. After the gate was drawn we counted how many non-SCDE particles (artefacts) were included in this gate to assess the false positivity rate. A high false positivity rate indicates bad clustering. This approach was done for all unique pairwise combinations of all measured parameters. Next to scatter-based parameters, we also included fluorescence-based parameters. This was done by including a fluorescent marker in the core emulsions (sulfoCy5-COOH, sCy5, Lumiprobe, Hannover, Germany).

The automated gating algorithm worked as follows. Because the scale of flow cytometric parameters differs on the scale of orders of magnitude a minmax normalisation was performed before calculating Euclidian distance so that each parameter was normalized to a range between 0 and 1:

$$x_{norm,i} = \frac{x_i - x_{min}}{x_{max} - x_{min}} \quad (6)$$

(6) x_i is a measured value in a FCM channel (AU). $x_{norm,i}$ is the normalized value with index i (/). x_{max} is the maximum measured value of all events in that FCM channel (AU) x_{min} is the minimum measured value of all events in that FCM channel (AU).

The Euclidean distance of all SCDE events was then calculated as follows:

$$d_i = \sqrt{\left(x_{norm,i} - median(x_{norm,SCDE})\right)^2 + \left(y_{norm,i} - median(y_{norm,SCDE})\right)^2} \quad (7)$$

(7) d_i is the Euclidean distance of event with index i. $x_{norm,i}$ is the normalized value with index i (/). x and y represent two values of different FCM channels (AU).

To avoid fitting the ellipsoid gate to outliers, the 5% most distant SCDE events were removed from the gating procedure. An ellipsoid gate was drawn around the remaining SCDE events using a minimum enclosing ellipse algorithm.[17] Finally, the false positivity rate was determined by checking how many non-SCDE particles fall within the drawn ellipsoid gate and applying following formula:

$$\%_{false\ positive} = \frac{n_{artefacts\ in\ gate}}{n_{artefacts\ in\ gate} + n_{SCDE\ in\ gate}} \quad (8)$$

(8) $n_{artefacts\ in\ gate}$ is the number of artefacts that fall within the drawn ellipsoid. $n_{SCDE\ in\ gate}$ is the number of SCDE particles that fall within the drawn ellipsoid.

Hydrogen-oxidizing communities DE-SWIRL screening

Post-aeration sludge from a municipal wastewater treatment plant (RWZI, Ghent, Belgium) was used for enrichment of aerobic hydrogen-oxidizing bacteria by vortexing (3 mL), centrifugation (5000×g, 5 min), washing once with 1× PBS, and dilution to an OD600 of 0.05

in mineral DSMZ-81 medium before incubation in Hungate tubes under a $H_2/CO_2/O_2$ (73/18/9%) atmosphere at 28 °C with shaking. Cultures were subcultured twice weekly (1:10) over four weeks during which growth was monitored by eye. After this enrichment phase enrichments were stored at 4 °C. Prior to droplet experiments, the enrichment was reactivated, harvested during exponential growth, washed three times with cold DSMZ-81 medium, and resuspended for inoculation. Droplets were inoculated at a starting cell density of 1×10^8 cells/mL, grown in Hungate tubes with the same custom atmosphere as before, and after 10 days they were harvested and converted to double emulsions using our protocol, sorted and regrown after sorting. The double emulsions were sorted into DSMZ-81 with 0.6 w% fructose, 0.6 w% pyruvic acid and 0.2 w% yeast extract. For staining, double emulsions were incubated for 30 min at 37 °C under agitation (100 rpm) with Sybr Green (Invitrogen, Thermo Fisher Scientific, Waltham, MA, USA), followed by three washes with 1× PBS (10 minutes, 100 rpm, 37 °C).

Mineral DSMZ-81 medium was prepared according to DSMZ specifications with several modifications. Three solutions were made: solution A was made by dissolving 2.3 g KH_2PO_4 and 2.9 g $Na_2HPO_4\cdot2H_2O$ in 50 mL distilled water. Solution B was made by dissolving the following in 920 mL distilled water: 4.0 g NH_4Cl (increased from 1.0 g), 0.5 g $MgSO_4\cdot7H2O$, 10 mg $CaCl_2\cdot2H_2O$, 5 mg $MnCl2\cdot4H_2O$, 5 mg $NaVO_3\cdot H_2O$, and 5 mL trace element solution SL-6. SL-6 is made of 0.1 g/L $ZnSO_4\cdot7H_2O$, 30 mg/L $MnCl_2\cdot4H_2O$, 0.3 g/L H_3BO_3, 0.2 g/L $CoCl_2\cdot6H_2O$, 10 mg/L $CuCl_2\cdot2H_2O$, 20 mg/L $NiCl_2\cdot6H_2O$, and 30 mg/L $Na_2MoO_4\cdot2H_2O$ in distilled water. Solution C was altered: 50 mg $FeSO_4\cdot7H_2O$ and 60 mg $Na_2EDTA\cdot2H_2O$ were dissolved in 20 mL distilled water. We altered this solution to avoid introducing bioavailable organic carbon from citrate which was used in the original recipe. Solutions A, B, and C were autoclaved separately for 15 min at 121 °C. All solutions were combined aseptically together with 10 mL/L 50 g/L $NaHCO_3$ solution. No vitamin solution was added. The final solution was filter sterilised and stored at room temperature. All aforementioned chemicals were bought from Sigma-Aldrich (Merck KGaA, Darmstadt, Germany).

For sorting medium, yeast extract (Carl Roth, Karlsruhe, Germany), pyruvic acid (Sigma-Aldrich, Merck KGaA, Darmstadt, Germany), and glucose (Sigma-Aldrich, Merck KGaA, Darmstadt, Germany), were prepared as 200 g/L stock solutions in distilled water and filter-sterilised. Filter sterilised mineral DSMZ-81 medium (prepared as described above) was supplemented with these stock solutions to final concentrations of 0.2% (w/v) yeast extract, 0.6% (w/v) pyruvic acid, and 0.6% (w/v) glucose. The final solution was stored at room temperature.

Calculating CV values from literature

Microscopy images were analyzed using Fiji (ImageJ). For analysis of the template single emulsions (for Sukovich et al. data [18]), the “Find Edges” function was applied, after which the images were converted to 8-bit grayscale and segmented using the automatic thresholding function. Particles were identified using “Analyze Particles”, with a minimum particle size of 300 pixels^2 and no upper size limit, and a circularity range of 0.60–1.00. The resulting particle measurements were used to determine the diameter distribution of the single emulsions.

Core diameters were measured manually (for Lin et al. (2022) and Sukovich et al. (2017) data [18,19]) in Fiji by drawing a diameter line over each double emulsion core and measuring the length of this line. At least 70 particles were measured for each condition. Oil droplets and small double emulsions were not included in these measurements. Each measurement set was obtained from one technical replicate. The reported CV therefore represents the within-sample particle-size variability and not variability between independent technical or biological replicates.

The coefficient of variation was calculated as:

$$\mathrm{CV}(\%) = \frac{\text{standard deviation}}{\text{mean}} \times 100$$

Supplementary information - References

1. Brower KK, Brower KK, Carswell-Crumpton C, Klemm S, Cruz B, Kim G, et al. Double emulsion flow cytometry with high-throughput single droplet isolation and nucleic acid recovery. Lab Chip. 2020;20: 2062–2074. doi:10.1039/d0lc00261e

2. Zhuang S, Semenec L, Nagy SS, Cain AK, Inglis DW. High-precision screening and sorting of double emulsion droplets. Cytometry Part A . 2024;105: 547–554. doi:10.1002/cyto.a.24842

3. Galbraith DW, Lucretti S. Large Particle Sorting. Flow Cytometry and Cell Sorting. 2000; 293–317. doi:10.1007/978-3-662-04129-1_25

4. Galbraith DW, Sun G. Flow Cytometry and Sorting in Arabidopsis. Methods in Molecular Biology. 2021;2200: 255–294. doi:10.1007/978-1-0716-0880-7_12/FIGURES/5

5. Basu S, Campbell HM, Dittel BN, Ray A. Purification of Specific Cell Population by Fluorescence Activated Cell Sorting (FACS). Journal of Visualized Experiments. 2010; 1546. doi:10.3791/1546

6. van Kesteren S, Diethelm P, Isa L. Fluorescence-activated cell sorting (FACS) for purifying colloidal clusters. Soft Matter. 2024;20: 2881–2886. doi:10.1039/d4sm00122b

7. Maes E, Cools N, Willems H, Baggerman G. FACS-based proteomics enables profiling of proteins in rare cell populations. Int J Mol Sci. 2020;21: 1–12. doi:10.3390/ijms21186557

8. Costa C, Medronho B, Filipe A, Mira I, Lindman B, Edlund H, et al. Emulsion formation and stabilization by biomolecules: The leading role of cellulose. Polymers. Multidisciplinary Digital Publishing Institute (MDPI); 2019. doi:10.3390/polym11101570

9. Wang J, Hahn S, Amstad E, Vogel N. Tailored Double Emulsions Made Simple. Advanced Materials. 2022;34: 2107338. doi:10.1002/adma.202107338

10. Zinchenko A, Devenish SRA, Kintses B, Colin PY, Fischlechner M, Hollfelder F. One in a million: Flow cytometric sorting of single cell-lysate assays in monodisperse picolitre double emulsion droplets for directed evolution. Anal Chem. 2014;86: 2526–2533. doi:10.1021/ac403585p

11. Métézeau Ph. Practical flow cytometry. Ann Inst Pasteur Immunol (1985). 1988;139: 593–594. doi:10.1016/0769-2625(88)90111-0

12. Holtze C, Rowat AC, Agresti JJ, Hutchison JB, Angilè FE, Schmitz CHJ, et al. Biocompatible surfactants for water-in-fluorocarbon emulsions. Lab Chip. 2008;8: 1632–1639. doi:10.1039/B806706F

13. Wagner O, Thiele J, Weinhart M, Mazutis L, Weitz DA, Huck WTS, et al. Biocompatible fluorinated polyglycerols for droplet microfluidics as an alternative to PEG-based copolymer surfactants. Lab Chip. 2015;16: 65–69. doi:10.1039/C5LC00823A

14. Mahler L, Niehs SP, Martin K, Weber T, Scherlach K, Hertweck C, et al. Highly parallelized droplet cultivation and prioritization on antibiotic producers from natural microbial communities. Elife. 2021;10: e64774. doi:10.7554/eLife.64774

15. Scott GD, Kilgour DM. The density of random close packing of spheres. J Phys D Appl Phys. 1969;2: 863. doi:10.1088/0022-3727/2/6/311

16. Schindelin J, Arganda-Carreras I, Frise E, Kaynig V, Longair M, Pietzsch T, et al. Fiji: An open-source platform for biological-image analysis. Nature Methods. Nature Publishing Group; 2012. pp. 676–682. doi:10.1038/nmeth.2019

17. Wollschlaeger D. shotGroups: Analyze Shot Group Data. R package version 0.8.2. In: 2022.

18. Sukovich DJ, Kim SC, Ahmed N, Abate AR. Bulk double emulsification for flow cytometric analysis of microfluidic droplets. Analyst. 2017;142: 4618–4622. doi:10.1039/c7an01695f

19. Lin S, Mao L, Ying J, Berthet N, Zhou J, Riaud A. Generation of double emulsions from commercial single-emulsion microfluidic chips: a quality-control study. Microfluid Nanofluidics. 2022;26: 1–13. doi:10.1007/s10404-022-02575-7
